# Switch from intravenous anti-CD20 therapy to subcutaneous ofatumumab in patients with relapsing MS: results from the OLIKOS study

**DOI:** 10.1007/s00415-025-13462-w

**Published:** 2025-10-24

**Authors:** Le H. Hua, Brandon Brown, Elizabeth Camacho, Angel R. Chinea, Benjamin M. Greenberg, Roland G. Henry, Erik Houtsma, Natalie Moreo, Enrique Alvarez

**Affiliations:** 1 Cleveland Clinic Lou Ruvo Center for Brain Health, 888 W. Bonneville Ave, Las Vegas, NV 89106 USA; 2 Novartis Pharmaceuticals Corporation, East Hanover, NJ USA; 3 San Juan MS Center, Guaynabo, Puerto Rico; 4 Department of Neurology, University of Texas Southwestern Medical Center, Dallas, TX USA; 5 UCSF Weill Institute for Neurosciences, University of California, San Francisco, CA USA; 6 Department of Neurology, University of South Florida, Tampa, FL USA; 7 Department of Neurology, Rocky Mountain MS Center at the University of Colorado, Aurora, CO USA

**Keywords:** Multiple sclerosis, Disease-modifying therapy, High-efficacy therapies, Monoclonal antibodies

## Abstract

**Background:**

Multiple sclerosis (MS) is a chronic condition, and as such, switching therapies is not uncommon. However, data on switching from intravenous (IV) to subcutaneous (SC) formulations of anti-CD20 therapies are lacking.

**Methods:**

OLIKOS, a phase 3b, prospective, single-arm, multicenter study conducted from 2020 to 2024, evaluated the efficacy and safety of switching to SC ofatumumab from IV ocrelizumab or rituximab in adults with relapsing MS. Participants were excluded if they had discontinued anti-CD20 therapy due to suboptimal response or safety concerns. Maintenance of efficacy was defined as either no change or a reduction from baseline in the number of gadolinium-enhancing (Gd +) T1 lesions observed by magnetic resonance imaging (MRI) after 12 months of ofatumumab.

**Results:**

The full analysis set included 102 participants. Most participants (99%) switched from IV ocrelizumab to SC ofatumumab. Zero Gd+ T1 lesions were observed at Month 12 in participants with evaluable MRI assessments (*n* = 84), satisfying the primary endpoint. New/enlarging T2 lesions were observed in 2.3% (2/86) of participants at Month 12. There was no change in median Expanded Disability Status Scale score between baseline and Month 12, and annualized relapse rate remained low (0.075). Treatment satisfaction improved from baseline to Month 12 across all domains with the largest increases in the Convenience domain. Treatment-emergent adverse events occurred at similar frequencies as in ofatumumab phase 3 trials, and no new safety signals were identified.

**Conclusion:**

The findings indicate efficacy and safety are maintained following a switch from IV anti-CD20 to SC ofatumumab with improved treatment satisfaction.

**Trial registration:**

ClinicalTrials.gov Identifier: NCT04486716 https://clinicaltrials.gov/study/NCT04486716

The online version of this article (10.1007/s00415-025-13462-w) contains supplementary material, which is available to authorized users.

## Introduction

Multiple sclerosis (MS) is a chronic inflammatory disorder of the central nervous system leading to neurologic disability [1, 2]. Because MS is a chronic condition, patients commonly switch therapies over the course of their disease; previous studies showed that 18–28% of patients changed treatments at least once within 2 years of initiating disease-modifying therapy (DMT), and nearly 50% of patients switched DMTs within a 3-year period [3–6]. Reasons for therapeutic changes primarily include efficacy or safety concerns; however, patients may also switch therapy due to route-of-administration preferences, family planning/pregnancy considerations, and changes in insurance [3, 7].

Once thought to be primarily driven by T cells, the pathophysiology of MS is now known to also be strongly influenced by B cells [8–10]. B-cell depletion with anti-CD20 therapies has been shown to reduce annualized relapse rate (ARR) and inflammatory lesion activity as well as delay disability worsening in relapsing multiple sclerosis (RMS) [11, 12]. While intravenous (IV) rituximab is commonly used off-label to treat patients with MS [13, 14], the first anti-CD20 therapy approved by the US Food and Drug Administration (FDA) for the treatment of RMS was IV ocrelizumab (March 2017) [15]. Typically, IV rituximab and IV ocrelizumab infusions are administered every 6 months in a medical setting. In August 2020, the FDA approved ofatumumab, a subcutaneous (SC) anti-CD20 therapy that patients can self-administer at home once monthly using an auto-injector pen [16].

There are currently no published studies examining switching from ocrelizumab for reasons unrelated to efficacy or safety. Furthermore, the phase 3 ASCLEPIOS trials (NCT02792218 and NCT02792231) with ofatumumab excluded participants with prior anti-CD20 exposure, further highlighting the need for data regarding participants with MS who have switched from IV anti-CD20 therapies to SC ofatumumab [17].

The aim of the OLIKOS study was to evaluate the efficacy and safety of switching to SC ofatumumab from either IV ocrelizumab or rituximab in participants with RMS that was stable without suboptimal response or safety concerns. Of note, 2 anti-CD20 therapies—IV ublituximab-xiiy [18] and SC ocrelizumab [19, 20]—received FDA approval after OLIKOS had commenced and therefore were not evaluated as part of this study.

## Materials and methods

### Study design

**Fig. 1:**
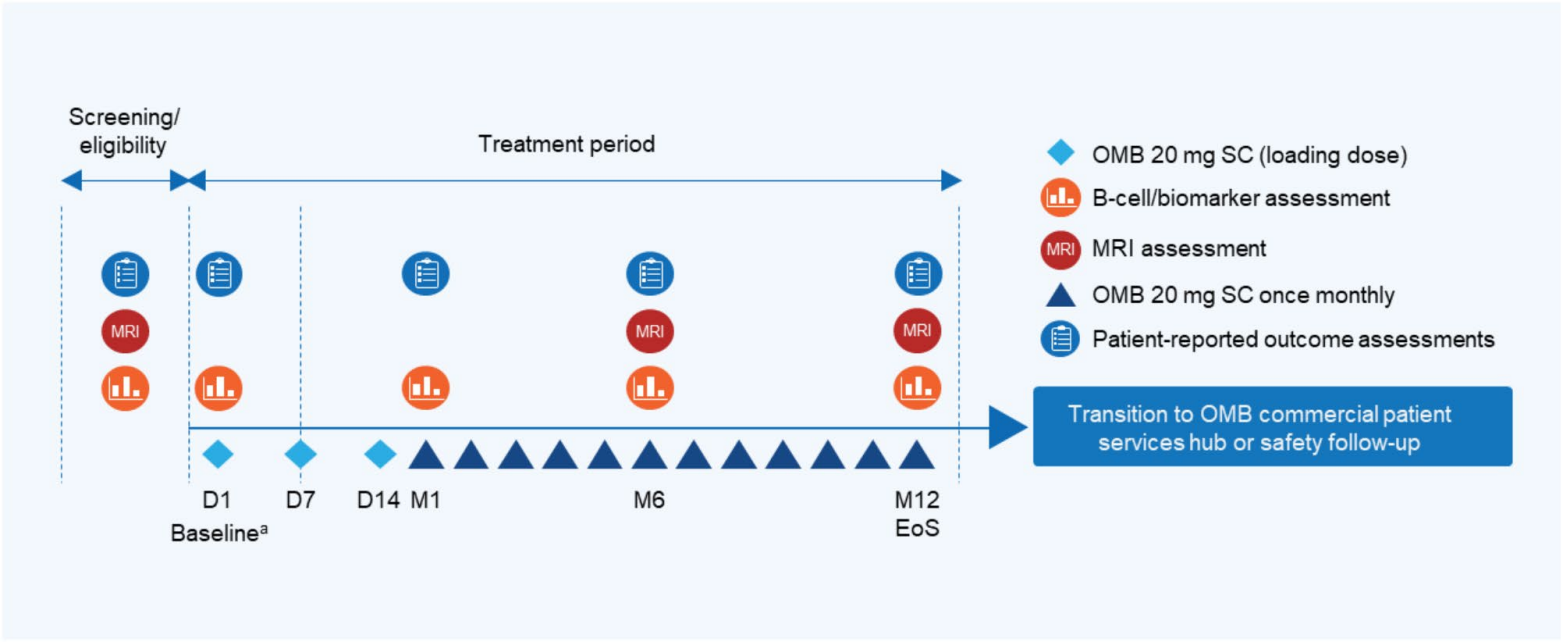
OLIKOS study design. ^a^All baseline assessments were performed prior to dispensing the study drug. *D* day, *EoS* end of study, *M* month, *MRI* magnetic resonance imaging, *OMB* ofatumumab, *SC* subcutaneous

OLIKOS (NCT04486716) was a single-arm, multicenter, phase 3b study conducted in the US among participants who transitioned from the IV anti-CD20 therapies ocrelizumab or rituximab to the SC anti-CD20 therapy ofatumumab. This study consisted of 3 parts: a 28-day screening period, a 12-month treatment period, and a 30-day telephone safety follow-up period (Fig. 1). If required, the posttreatment safety follow-up period was extended until either another therapy was initiated or B cells were repleted. Participants were also able to start on commercial ofatumumab after study completion. The minimum duration of the study was 14 months. The dose regimen for ofatumumab consisted of a loading dose of 20 mg on Days 1, 7, and 14, followed by a once-monthly maintenance dose of 20 mg starting at Month 1. Following Day 1, ofatumumab administration could be completed at home or at the study site, dependent on participant preference. The study protocol is available in Supplement 1.

OLIKOS was designed, implemented, executed, and reported in accordance with the International Conference on Harmonization (ICH) Harmonized Tripartite Guidelines for Good Clinical Practice, with applicable local regulations (including European Directive 2001/20/EC, US CFR 21), and with the ethical principles laid down in the Declaration of Helsinki.

### Study population

The trial enrolled neurologically stable participants aged 18–60 years who were diagnosed with RMS using the 2017 McDonald criteria [21], had an Expanded Disability Status Scale (EDSS) score of ≤ 5.5, and had received at least 2 courses of IV anti-CD20 therapy with either ocrelizumab or rituximab, with the last dose given within the last 4–9 months. Individuals who previously experienced a suboptimal response to anti-CD20 therapy (the occurrence of relapses, lesions, or disability within predefined timelines) or discontinued therapy due to a treatment-related adverse event (e.g., severe infusion-related reaction, recurrent infections, decreased immunoglobulin G [IgG] level requiring treatment) were excluded. Additional populations excluded from the trial were individuals with primary progressive MS, pregnant or nursing women, individuals with active chronic disease of the immune system (apart from MS), and those at risk of reactivation of syphilis, tuberculosis, or hepatitis.

### Study outcomes

The primary objective of the study was to assess maintenance of efficacy of ofatumumab in participants with RMS transitioning from an IV anti-CD20 therapy. Maintenance of efficacy was measured by evaluating whether participants experienced either no change or a reduction from baseline in the number of gadolinium-enhancing (Gd +) T1 lesions observed by magnetic resonance imaging (MRI) after 12 months of ofatumumab treatment (yes/no). This outcome was chosen based on the inclusion/exclusion criterion that allowed participants to have up to a single Gd+ T1 lesion on their baseline MRI.

Secondary objectives included characterizing safety and tolerability, treatment satisfaction, and participant retention. Safety and tolerability were assessed by the occurrence of treatment-emergent adverse events (TEAEs). Treatment satisfaction was determined by the change from baseline to Months 1, 6, and 12 in the abbreviated 9-item Treatment Satisfaction Questionnaire for Medication (TSQM-9), a validated tool with a scale that ranges from 0 (indicating poor satisfaction) to 100 (indicating perfect satisfaction) and includes the domains of Effectiveness, Convenience, and Global satisfaction [22]. Retention on ofatumumab treatment from baseline to Month 6, and to Month 12 (yes/no), was also measured.

Exploratory outcomes included the number of relapses during the study, number of new or enlarging T2 lesions, and change from baseline in EDSS score.

### Statistical analysis

The sample size calculation was based on the primary endpoint (proportion of participants with no change or a reduction from baseline in the number of Gd+ T1 lesions 12 months after initiating ofatumumab therapy). The sample size of 100 participants provided an 8.5% precision (half-width of 95% confidence interval), a 7.8% precision, or a 7.0% precision corresponding to estimated proportions of 75%, 80%, and 85% of participants achieving the primary endpoint.

The full analysis set (FAS) consisted of all participants who received at least 1 dose of study drug. The FAS was used for the summary of demographic and baseline characteristics as well as for all efficacy analyses. The safety analysis set was identical to the FAS in this study.

The number and percentage of participants with no change or a reduction in the number of Gd+ T1 lesions at Month 12 were reported. The 95% confidence interval for the proportion of participants with no change or a reduction was calculated using the normal approximation method. Both non-responder imputation for missing data and observed data approaches were applied. The number and percentage of participants with adverse events (AEs) were summarized by Medical Dictionary for Regulatory Activities (MedDRA) Preferred Term and severity. Changes from baseline in EDSS scores, TSQM-9 scores, CD19+ B cells, and IgG/IgM levels were summarized descriptively.

## Results

### Participant disposition

**Fig. 2:**
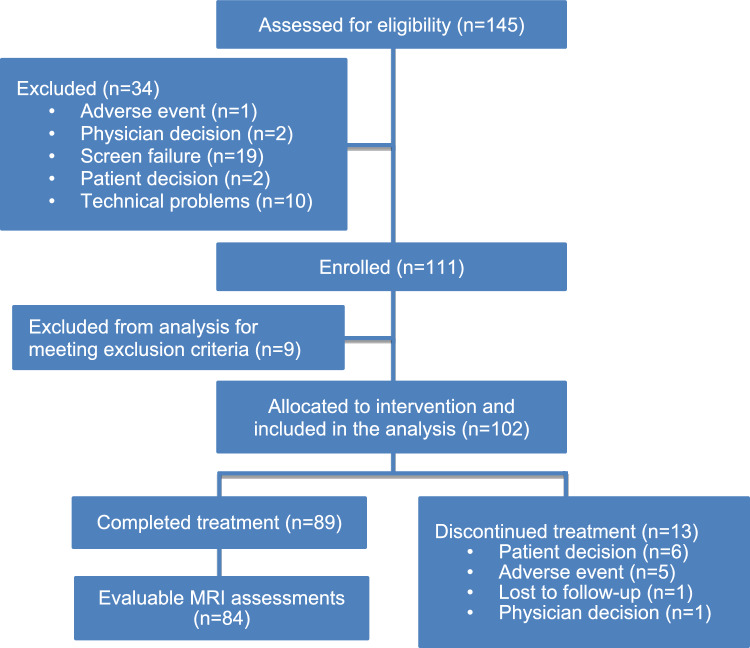
OLIKOS patient flow chart

A total of 145 participants were screened for inclusion in OLIKOS (Fig. 2). Following 34 screen failures, 111 participants were enrolled. A total of 102 participants received ofatumumab 20 mg and were included in the analysis. Of the 102 participants who received ofatumumab 20 mg (FAS), 89 (87.3%) completed treatment and 13 (12.7%) discontinued treatment.

Eighteen participants were not included in the observed case analysis of the primary endpoint due to lack of baseline MRI (1 of 18), protocol deviation (2 of 18), MRI assessment outside of the 12-month window (2 of 18; ≥ 30 days after last study treatment administration date), or lack of MRI at the Month 12 visit (13 of 18). Five participants were not included in the non-responder imputation due to the use of prohibited concomitant medication (2 of 5), MRI assessment outside of the 12-month window (2 of 5), or lack of baseline MRI (1 of 5).

### Demographics and baseline clinical characteristics

Most participants were female (67.6%) and White (76.5%), with a mean duration of 9.4 years since MS diagnosis (Table 1). Notably, 19.6% identified as Black or African American and 29.4% identified as Hispanic or Latino. One patient had a Gd+ T1 lesion at baseline. Nearly all (99%) participants were receiving IV ocrelizumab prior to switching to SC ofatumumab.

**Table 1:** Patient demographics and baseline clinical characteristics

Characteristic	OMB 20 mg SC (*N* = 102)
Age, mean (SD), years	43.5 (8.2)
Female, *n* (%)	69 (67.6)
Race, *n* (%)	
White	78 (76.5)
Black or African American	20 (19.6)
Asian	3 (2.9)
Unknown	1 (1.0)
Ethnicity, *n* (%)	
Not Hispanic or Latino	70 (68.6)
Hispanic or Latino	30 (29.4)
Not reported	2 (2.0)
BMI, mean (SD), kg/m^2^	29.3 (7.3)
Baseline EDSS score	
Mean (SD)	2.9 (1.4)
Median (range)	2.75 (0.0–5.5)
Gd+ T1 lesions,^a^ *n*	
Mean (SD)	0.01 (0.1)
Median	0
Gd+ T1 lesions present at baseline (yes), *n* (%)	1 (1.0)
Duration of MS since diagnosis, mean (SD), years	9.4 (7.1)
Type of MS at study entry, *n* (%)	
RRMS	100 (98.0)
SPMS	2 (2.0)
Previous MS IV anti-CD20 therapy, *n* (%)	
Rituximab	1 (1.0)
Ocrelizumab	101 (99.0)
Duration of previous IV anti-CD20 therapy, mean (SD), months	
Rituximab	33.97 (NA)
Ocrelizumab	26.70 (15.25)
Time between last infusion and baseline visit, months	
Rituximab	
Mean (SD)	6.67 (NA)
Ocrelizumab	
Mean (SD)	6.36 (1.55)
Median (range)	6.18 (2.1–11.7)

EDSS score ranges from 0 (normal) to 10 (death due to MS) in 0.5-unit increments. Duration of MS since diagnosis (years) is derived ([first dose date–MS diagnosis start date+ 1]/365.25)

*n* number of patients with a measurement (for continuous variables); *N* number of patients in the FAS/SAF. Percentages are computed using *N* as the denominator

*BMI* body mass index, *EDSS* Expanded Disability Status Scale, *FAS* full analysis set, *Gd*+ gadolinium-enhancing, *IV* intravenous, *MS* multiple sclerosis, *NA* not applicable, *OMB* ofatumumab, *RRMS*, relapsing–remitting multiple sclerosis, *SAF* safety analysis set, *SC* subcutaneous, *SD* standard deviation, *SPMS* secondary progressive multiple sclerosis

^a^*n* = 101

### Efficacy

#### Primary efficacy outcome

Among participants with evaluable MRI assessments (*n* = 84), 100% had no change or a reduction from baseline in the number of Gd+ T1 lesions at Month 12 (Table 2). For the MRIs that were performed outside of the 12-month window, zero Gd+ T1 lesions were observed as well. Using non-responder imputation for missing data in the FAS, 86.6% (*n* = 84 of 97) of participants had no change or a reduction from baseline in the number of Gd+ T1 lesions at Month 12.

**Table 2:** Efficacy outcomes at Month 6 and/or Month 12

Primary efficacy outcome	Month 12
Gd+ T1 lesions	
Observed case analysis (no change or reduction from baseline), n/M (%)	84/84 (100)
Non-responder imputation analysis (no change or reduction from baseline), n/M (%)	84/97 (86.6)

Exploratory efficacy outcomes	Month 12
New or enlarging T2 lesions (yes), n/M (%)	
Brain	1/86 (1.2)
Upper cervical spinal cord	1/86 (1.2)
EDSS^a^	
Change in EDSS score from baseline, median (min–max)	0.0 (− 2.0 to 3.0)
Relapses	
ARR^b^	0.075
	Month 6

New or enlarging T2 lesions (yes), n/M (%)	Month 12
Brain	3/94 (3.2)
Upper cervical spinal cord	2/94 (2.1)

*n* number of patients with a measurement (for continuous variables); *M* number of patients with an evaluable assessment at the named time point

*ARR* annualized relapse rate, *EDSS* Expanded Disability Status Scale, *Gd*+ gadolinium-enhancing

^a^Median calculated for participants with evaluable assessments at baseline and Month 12 (*n* = 89)

^b^Annualized relapse rate (time-based) is calculated by taking the total number of relapses observed for all participants within a treatment group, divided by the total number of days in study of all participants within the treatment group and multiplied by 365.25 days

#### Exploratory efficacy outcomes

New or enlarging T2 lesions were observed in 5.3% (5 of 94) of participants at Month 6 and 2.3% (2 of 86) of participants at Month 12 (Table 2). Three of the T2 lesions observed at Month 6 were in the brain and two were in the upper cervical spinal cord; at Month 12, one T2 lesion was observed in the brain and one in the upper cervical spinal cord. One of the two participants with new or enlarging T2 lesions at Month 12 had multiple relapses before screening and should have been excluded from the study. None of the five participants with new or enlarging T2 lesions at Month 6 showed new or enlarging T2 lesions at Month 12.

The median EDSS score at baseline for all participants (*n* = 102) was 2.75. For participants with a result at baseline and Month 12 (*n* = 89), the median EDSS score at both timepoints was 2.50.

The mean ARR (time-based) was 0.075 (Table 2). Six participants had at least one MS relapse during the study. Within this group, 1 participant experienced 2 MS relapses (Days 64 and 252) that led to study drug discontinuation; this participant also had a new T2 lesion at Month 6. Another participant who experienced an on-study relapse (Day 213) also had a newly enlarging T2 lesion at Month 6.

### Safety

#### Adverse events

Overall, a total of 86 (84.3%) participants experienced at least 1 TEAE (Table 3), with 42 (41.2%) participants experiencing TEAEs that were suspected to be related to the study treatment. The most frequent TEAEs (incidence > 5%) were COVID-19 infection (30.4%), headache (13.7%), fatigue (11.8%), urinary tract infection (10.8%), pruritus (5.9%), and pyrexia (5.9%). The most common injection-site and injection-systemic reactions were injection-site pain (3.9%) and headache (4.9%), respectively. Serious AEs (SAEs) were reported in six (5.9%) participants and included COVID-19 infection, infective myositis, urosepsis, sciatic nerve injury, vascular graft complication, pain in extremity, cerebrovascular accident, and deep vein thrombosis. Four (3.9%) participants discontinued the study treatment due to TEAEs, including vertigo, nausea, migraine, infective myositis, deep vein thrombosis, MS relapse, and injection-related reaction. No deaths occurred during the study.

**Table 3:** Treatment-emergent adverse events

	OMB 20 mg SC (*N* = 102)
All TEAEs, *n* (%)	
Patients with ≥ 1 TEAE	86 (84.3)
Patients with TEAEs leading to permanent study drug discontinuation	4 (3.9)
Patients with any SAE	6 (5.9)
Most common TEAEs,^a^ *n* (%)	
COVID-19 infection	31 (30.4)
Headache	14 (13.7)
Fatigue	12 (11.8)
Urinary tract infection	11 (10.8)
Pruritus	6 (5.9)
Pyrexia	6 (5.9)
Arthralgia	5 (4.9)
Dizziness	5 (4.9)
Nasal congestion	5 (4.9)
Sinusitis	5 (4.9)
SAEs, *n* (%)	
COVID-19 infection	1 (1.0)
Infective myositis	1 (1.0)
Urosepsis	1 (1.0)
Sciatic nerve injury	1 (1.0)
Vascular graft complication	1 (1.0)
Pain in extremity	1 (1.0)
Cerebrovascular accident	1 (1.0)
Deep vein thrombosis	1 (1.0)
Injection-site reactions, *n* (%)	10 (9.8)
Injection-site pain	4 (3.9)
Erythema	2 (2.0)
Injection-site hemorrhage	2 (2.0)
Injection-site pruritus	2 (2.0)
Pruritus	2 (2.0)
Injection-systemic reactions, *n* (%)	17 (16.7)
Headache	5 (4.9)
Pruritus	4 (3.9)
Chills	3 (2.9)
Dizziness	3 (2.9)
Fatigue	3 (2.9)
Nausea	2 (2.0)
Pyrexia	2 (2.0)

TEAEs causing study drug discontinuations refer to those with “action taken with study treatment” answered with “drug withdrawn”

Injection-site and injection-systemic reactions reported in > 1 participant are presented

Sites queried patients regarding injection-site/injection-systemic reactions during monthly remote contact around the time of OMB administration

*n* number of patients with a measurement (for continuous variables); *N* number of patients in the FAS/SAF. Percentages are computed using N as the denominator

*FAS* full analysis set, *OMB* ofatumumab, *SAE* serious adverse event, *SAF* safety analysis set, *SC* subcutaneous, *TEAE* treatment-emergent adverse event

^a^TEAEs reported by ≥ 5 patients

#### Hematology parameters

Mean CD19+ B-cell concentrations were well below the normal reference range (107–698 cells/μL) at baseline, with 7 of 102 (6.9%) participants having repleted their CD19+ B-cell concentrations to within the normal reference range. The mean time since the last infusion for these seven patients was 7.8 months, with a maximum of 9.1 months. Mean (standard deviation [SD]) CD19+ B-cell concentration decreased from baseline (19.9 [50.49] cells/µL) to Month 12 (2.3 [12.91] cells/µL) (n = 86) after switching to ofatumumab, with zero participants repleting their CD19+ B cells to within the normal reference range between doses throughout the study.

Mean (SD) serum IgG and IgM concentrations were within the normal reference ranges (IgG: 7.00–16.00 g/L; IgM: 0.40–2.30 g/L) at baseline (IgG: 9.92 [2.86] g/L; IgM: 0.56 [0.35] g/L) and remained within the normal reference ranges at Month 12 (IgG: 9.66 [2.99] g/L; IgM: 0.51 [0.32] g/L) (n = 89). At baseline, 17 (16.7%) and 36 (35.3%) participants had IgG and IgM levels, respectively, below the lower limit of normal (LLN). At Month 12, 17 (16.7%) and 38 (37.3%) participants had IgG and IgM levels, respectively, below LLN; however, no values of clinical concern were reported.

### Retention and treatment satisfaction

Retention on SC ofatumumab was high, with 93.1% and 87.3% of participants continuing ofatumumab at Month 6 and Month 12, respectively.

**Fig. 3:**
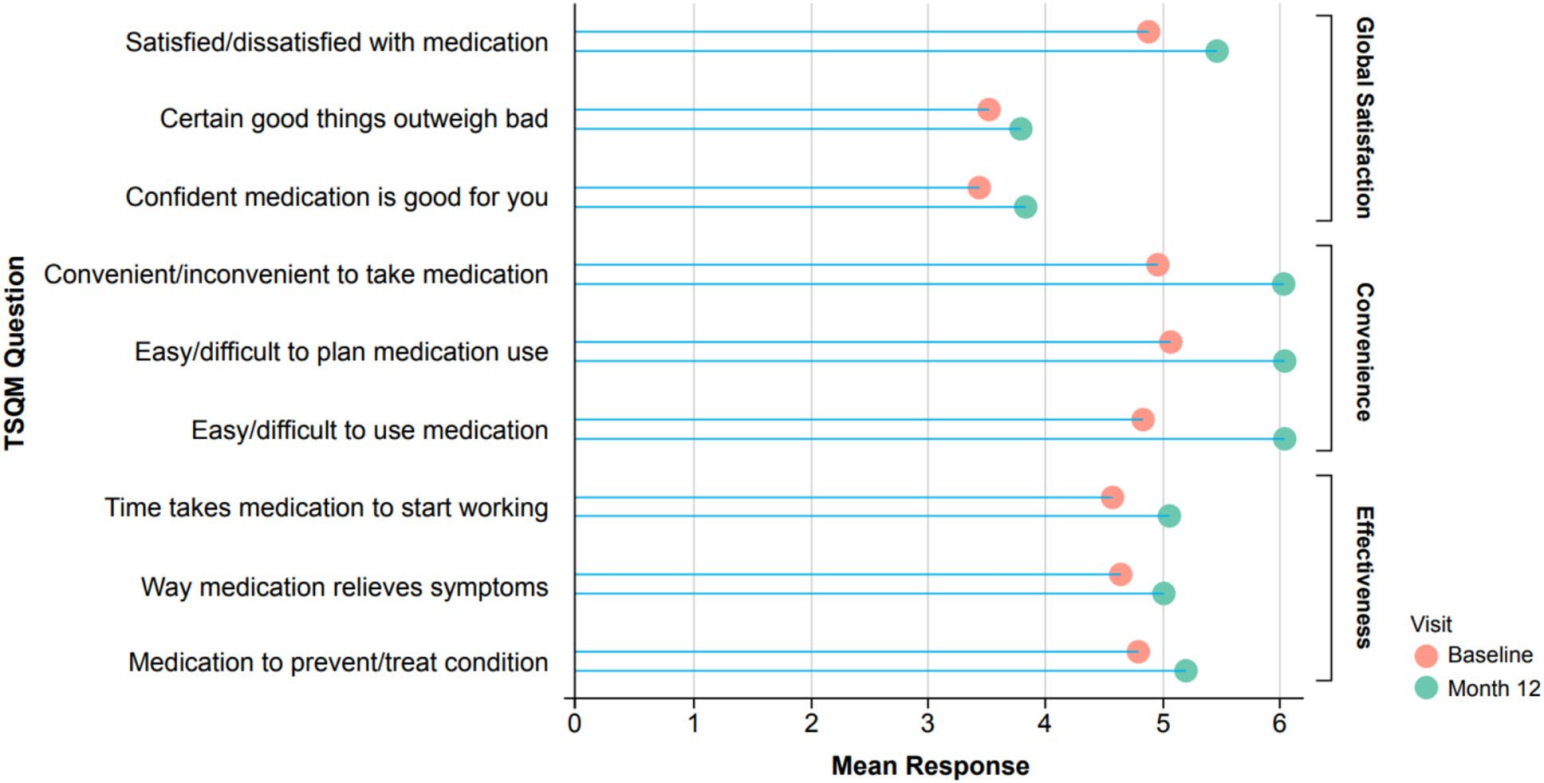
Treatment satisfaction at baseline and Month 12. *TSQM* Treatment Satisfaction Questionnaire for Medication

Treatment satisfaction, as measured by mean responses to the TSQM-9 (n = 42), improved from baseline to Month 12 for all sub-questions (Fig. 3). The largest increases were seen in the Convenience domain (mean change [SD], 16.40 [23.12]), followed by Global satisfaction (9.33 [30.66]), and Effectiveness (8.60 [22.90]).

## Discussion

As MS is a chronic condition, medication switches are common in response to suboptimal efficacy, undesirable AEs, inconvenience, pregnancy, and/or changes in insurance [3, 7]. OLIKOS was the first study to assess whether efficacy and safety are maintained in participants switching from IV anti-CD20 therapies to SC ofatumumab. In this phase 3b study, participants who were stable on IV anti-CD20 therapies maintained efficacy 12 months after transitioning from IV ocrelizumab or rituximab to the SC anti-CD20 therapy ofatumumab, as demonstrated by the lack of Gd+ T1 lesions during the study period. New or enlarging T2 lesions were also relatively rare, occurring in approximately 2% of participants at Month 12. Furthermore, there was no increase in patient disability, with median EDSS scores remaining unchanged from baseline to Month 12 in participants with evaluable assessments at both timepoints. ARR remained low at 0.075, consistent with the phase 3 ASCLEPIOS trials [17].

TEAEs occurred at similar frequencies as in the ASCLEPIOS trials, and no new safety signals were identified. Mean IgG and IgM concentrations remained within normal reference ranges throughout the study with minimal numerical changes, although longer periods of follow-up and more participants are needed to determine whether switching to ofatumumab from IV anti-CD20 therapies affects Ig levels. These safety results further support the conclusions of a recent systematic literature review showing that participants treated with ofatumumab demonstrated stable IgG levels over time [23]. CD19+ B-cell concentrations were maintained or reduced to below LLN from baseline to Month 12. However, mean/median CD19+ B-cell concentrations were lower once participants switched to ofatumumab, suggesting greater repletion of B cells between twice-yearly doses of ocrelizumab than with monthly ofatumumab doses. Furthermore, six of the seven individuals who had normal B-cell concentrations at baseline and recorded values post baseline showed sustained B-cell depletion throughout the study after switching to ofatumumab.

Treatment satisfaction increased among participants from baseline to Month 12, with higher scores across all domains. An observational, real-world study found that nearly two-thirds of patients with RMS taking ofatumumab described the therapy as having a “very positive” (44%) or “positive” (19%) impact on their quality of life [24]. Self-administration at home may be a preferred approach for some patients with MS, especially those for whom travel to IV infusion centers and/or health care provider offices is challenging (e.g., patients with advanced disability with limitations in transportation and/or patients in rural areas). Previous evidence has demonstrated that in people living with MS, adherence is higher among those with higher treatment satisfaction [25–27].

### Study limitations

The study has some limitations, including its open-label nature, lack of comparator group, and no long-term follow-up after switching to commercial ofatumumab. In addition, as participants chose to switch, there may be potential bias regarding the satisfaction scores, which limits the generalizability of the treatment satisfaction data.

Due to the 12-month duration of this study, the results may be impacted by prolonged B-cell depletion after treatment with IV anti-CD20s which has been observed in previous studies [28, 29]. However, we observed significant B-cell repletion at baseline in this group of participants with subsequent depletion throughout the study and maintenance of efficacy with monthly ofatumumab. Future studies are needed to investigate switching between anti-CD20 therapies based on B-cell repletion levels.

As this study was initiated prior to the approval of ublituximab, future studies to investigate the maintenance of efficacy and safety when switching from IV to SC anti-CD20 therapies should include participants switching from ublituximab. Similarly, future studies are needed to investigate switching between SC anti-CD20 therapies since the approval of ocrelizumab and hyaluronidase-ocsq, a formulation of ocrelizumab for SC administration by health care professionals via slow push into the abdomen every 6 months [19, 20].

## Conclusion

The findings of this study indicate that efficacy and safety are maintained following a switch from IV ocrelizumab (or rituximab) to SC ofatumumab among participants with RMS who are clinically stable on their IV anti-CD20 therapy.

## Electronic supplementary material

Below is the link to the electronic supplementary material.

Supplementary material 1 (PDF 3058 kb)

## Declarations

### Data availability

The trial data are available on reasonable request, provided that it is in line with current ethical and intellectual property requirements surrounding the use of data. Requests should be directed through ClinicalStudyDataRequest.com.
