# Supplementary material for: Switch from intravenous anti-CD20 therapy to subcutaneous ofatumumab in patients with relapsing MS: results from the OLIKOS study
Source: J Neurol. 2025 Oct 24;272(11):725. doi: 10.1007/s00415-025-13462-w (PMC12592291; doi:10.1007/s00415-025-13462-w)
Supplement: Supplementary file 1 — Supplementary material 1 (PDF 3058 kb) [file 415_2025_13462_MOESM1_ESM.pdf]

NOVARTIS

Novartis Research and Development

OMB157

Clinical Trial Protocol COMB157GUS07 / NCT04486716

A single-arm, prospective, multi-center, study to explore maintained efficacy with ofatumumab therapy in patients with relapsing Multiple Sclerosis who discontinue intravenously delivered anti-CD20 monoclonal antibody (aCD20 mAb) therapy (OLIKOS)

Document type:	Amended Protocol Version
EUDRACT number:	N/A
Version number:	v02 (clean)
Clinical Trial Phase:	IIIb
Release date:	28-Jun-2022

Property of Novartis/Sponsor
Confidential
May not be used, divulged, published, or otherwise disclosed
without the consent of Novartis/Sponsor
Clinical Trial Protocol Template Version 4.0 dated 15-Feb-2021

[redacted]

Table of contents

Table of contents 2
List of tables 5
List of figures 6
List of abbreviations 7
Glossary of terms 10
Protocol summary 17
1 Introduction 21
1.1 Background 21
1.2 Purpose 22
2 Objectives, endpoints and estimands 23
2.1 Primary estimands 24
2.2 Secondary estimands 24
3 Study design 25
3.1 Remote procedures 26
4 Rationale 27
4.1 Rationale for study design 27
4.2 Rationale for dose/regimen and duration of treatment 28
4.3 Rationale for choice of control drugs (comparator/placebo) or combination drugs 28
4.4 Purpose and timing of interim analyses/design adaptations 28
4.5 Risks and benefits 28
4.6 Rationale for Public Health Emergency mitigation procedures 29
5 Population 30
5.1 Inclusion criteria 30
5.2 Exclusion criteria 31
6 Treatment 35
6.1 Study treatment 35
6.1.1 Investigational drug 35
6.1.2 Additional study treatments 35
6.1.3 Treatment arms/group 35
6.1.4 Treatment duration 35
6.1.5 Post Trial Continuity 35
6.2 Other treatment(s) 36
6.2.1 Concomitant therapy 36
6.2.2 Prohibited medication 37
6.2.3 Recommended treatment of MS Relapse 38

[redacted]

6.3 Participant numbering, treatment assignment, randomization 38
6.3.1 Participant numbering 38
6.3.2 Treatment assignment, randomization 38
6.4 Treatment blinding....38
6.5 Dose escalation and dose modification....39
6.5.1 Dose modifications....39
6.5.2 Follow-up for toxicities....39
6.5.3 Drug Interruptions....39
6.6 Additional treatment guidance....39
6.6.1 Transition period from previous aCD20 DMT 39
6.6.2 Treatment compliance....40
6.6.3 Emergency breaking of assigned treatment code....40
6.7 Preparation and dispensation....40
6.7.2 Instruction for prescribing and taking study treatment....41
7 Informed consent procedures 42
8 Visit schedule and assessments 43
8.1 Screening 50
8.1.1 Eligibility screening 50
8.1.2 Site contact with participants during self/home drug administration....50
8.1.3 Information to be collected on screening failures....50
8.2 Participant demographics/other baseline characteristics 51
8.3 Efficacy....51
8.3.1 Magnetic Resonance Imaging (MRI)....51
[redacted] [redacted] 53
[redacted] [redacted] 54
[redacted] [redacted] 54
8.3.5 Treatment Satisfaction Questionnaire for Medication TSQM- 9....55
[redacted] [redacted] 55
[redacted] [redacted] 55
8.3.8 Appropriateness of efficacy assessments....55
[redacted] [redacted] 56
8.4 Safety 56
8.4.1 Physical Examination....56
8.4.2 Electrocardiogram (ECG) 57
8.4.3 Vital Signs....57
8.4.4 Height and Weight 58
8.4.5 Laboratory evaluations....58

[redacted]

8.4.6 Pregnancy and assessments of fertility 60
8.4.7 Appropriateness of safety measurements 60
9 Discontinuation and completion 61
9.1 Discontinuation from study treatment and study 61
9.1.1 Discontinuation of study treatment 61
9.1.2 Discontinuation from study 62
9.1.3 Lost to follow-up 62
9.2 Withdrawal of informed consent/Opposition to use 63
9.3 Study completion and post-study treatment 63
9.4 Early study termination by the sponsor 64
10 Safety monitoring and reporting 64
10.1 Definition of adverse events and reporting requirements 64
10.1.1 Adverse events 64
10.1.2 Serious adverse events 66
10.1.3 SAE reporting 67
10.1.4 Reporting MS relapse as a SAE 68
10.1.5 Reporting of Disability Worsening as a SAE 68
10.1.6 Pregnancy reporting 69
10.1.7 Pregnancies 69
10.1.8 Reporting of study treatment errors including misuse/abuse 70
10.2 Additional Safety Monitoring 70
10.2.1 Liver safety monitoring 70
10.2.2 Renal safety monitoring 71
10.2.3 Prospective suicidality assessment 72
10.2.4 Data Monitoring Committee 72
10.2.5 Steering Committee 72
11 Data Collection and Database management 72
11.1 Data collection 72
11.2 Database management and quality control 73
11.3 Site monitoring 73
11.4 Potential Remote Study Visits 74
12 Data analysis and statistical methods 74
12.1 Analysis sets 74
12.2 Participant demographics and other baseline characteristics 75
12.3 Treatments 75
12.4 Analysis of the primary endpoint(s) 75
12.4.1 Definition of primary endpoint(s) 75

[redacted]

12.4.2 Statistical model, hypothesis, and method of analysis 75
12.4.3 Handling of intercurrent events of primary estimand 75
12.4.4 Handling of missing values/censoring/discontinuations 75
12.4.5 Sensitivity and Supportive analyses 75
12.5 Analysis of secondary endpoints 76
12.5.1 Efficacy endpoint(s) 76
12.5.2 Safety endpoints 76
12.5.3 Biomarkers 77
[redacted] 77
12.7 Interim analyses 78
12.8 Sample size calculation 78
12.8.1 Primary endpoint(s) 78
13 Ethical considerations and administrative procedures 79
13.1 Regulatory and ethical compliance 79
13.2 Responsibilities of the Investigator and IRB/IEC 79
13.3 Publication of study protocol and results 79
13.4 Quality Control and Quality Assurance 79
14 Protocol adherence 80
14.1 Protocol amendments 80
15 References 81
16 Appendices 84
16.1 Appendix 1: Liver event and Laboratory trigger Definitions and Follow-up Requirements 84
16.2 Appendix 2: Specific Renal Alert Criteria and Actions and Event Follow-up 88
16.3 Appendix 3: Safety Monitoring Guidance 89
16.3.1 Guidance on monitoring of Participants with symptoms of neurological deterioration suggestive of PML 89
16.3.2 Guidance on monitoring Participants with infections 90
16.3.3 Guidance on immunization 91
16.3.4 Guidance on monitoring of Participants with low immunoglobulin levels 91

List of tables

Table 2-1 Objectives and related endpoints 23
Table 6-1 Prohibited medication 37
Table 8-1 Assessment Schedule 44
Table 8-2 Assessment Schedule, Safety Follow-up 49
[redacted] 54

[redacted]

Table 8-4 Local ECG collection plan....................57
Table 8-5 Laboratory Assessments....................58
Table 10-1 Criterion for disability worsening based on change in EDSS score......69
Table 10-2 Guidance for capturing the study treatment errors including misuse/abuse....................70
Table 16-1 Liver event and laboratory trigger definitions....................84
Table 16-2 Follow up requirements for liver events and laboratory triggers...........84
Table 16-3 Specific Renal Alert Criteria and Actions....................88
Table 16-4 Renal Event Follow up....................89

List of figures

Figure 3-1 Study design....................26

[redacted]

List of abbreviations

aCD20 mAb	anti-CD20 monoclonal antibody
ADCC	Antibody-Dependent Cell-mediated Cytotoxicity
AE	adverse event
AESI	adverse event of special interest
AI	Autoinjector
AIDS	Acquired Immunodeficiency Syndrome
ALP	alkaline phosphatase
ALT	alanine aminotransferase
ARR	Annual Relapse Rate
AST	aspartate aminotransferase
BSL	Baseline
BUN	blood urea nitrogen
CDC	Complement-Dependent Cytotoxicity
CIS	Clinically isolated syndrome
CMO&PS	Chief Medical Office & Patient Safety
CNS	Central Nervous System
CO	Country Organization
COA	Clinical Outcome Assessment
CRF	Case Report/Record Form (paper or electronic)
CSF	Cerebrospinal Fluid
C-SSRS	Columbia-Suicide Severity Rating Scale (paper)
CTC	Common Toxicity Criteria
CTT	Clinical Trial Team
DMC	Data Monitoring Committee
DMF	Dimethyl Fumarate
DMQ	Daily Mood Questions
DMT	Disease Modifying Therapy
DNA	Deoxyribonucleic Acid
DWI	Diffusion-weighted Imaging
EAE	Experimental Autoimmune Encephalomyelitis
EC	Ethics Committee
ECG	Electrocardiogram
EDC	Electronic Data Capture
EDD	Expected Delivery Date
EDSS	Expanded Disability Status Scale
EMA	European Medicines Agency
EOS	End of Study
FACS	Fluorescence-activated cell sorting
FAS	Full Analysis Set
FDA	Food and Drug Administration

[redacted]

FPFV	First patient first visit
FS	Functional Score
GCP	Good Clinical Practice
Gd	Gadolinium
[redacted]	[redacted]
h	Hour
HBV	Hepatitis B Virus
hCG	Human Chorionic Gonadotropin
HCV	Hepatitis C Virus
HDL	High Density Lipoprotein
HIV	human immunodeficiency virus
ICH	International Conference on Harmonization of Technical Requirements for Registration of Pharmaceuticals for Human Use
IEC	Independent Ethics Committee
Ig	Immunoglobulin
INR	International Normalized Ratio
IPS	Information Processing Speed
IRB	Institutional Review Board
IRT	Interactive Response Technology
IUD	Intrauterine Device
IUS	Intrauterine System
iv	Intravenous
KM	Kaplan-Meier
LDH	lactate dehydrogenase
LDL	Low Density Lipoprotein
LFT	Liver function test
LLN	lower limit of normal
mAb	Monoclonal Antibody
MedDRA	Medical dictionary for regulatory activities
mg	milligram(s)
ml	milliliter(s)
MRI	Magnetic Resonance Imaging
MS	Multiple Sclerosis
[redacted]	[redacted]
NIH	National Institute of Health
OHP	Off-site Healthcare Professional
OMB	Ofatumumab
PCR	Protein-Creatinine Ratio
PD	pharmacodynamic(s)
[redacted]	[redacted]
PK	pharmacokinetic(s)
PML	Progressive Multifocal Leukoencephalopathy

[redacted]

[redacted]	[redacted]
PT	Prothrombin Time
PTA	Post-Trial Access
q4	Every 4
QMS	Quality Management System
RBC	red blood cell(s)
RPR	Rapid Plasma Reagin
RRMS	Relapsing Remitting Multiple Sclerosis
RMS	Relapsing Multiple Sclerosis
SAE	serious adverse event
SC	Steering Committee
sCR	serum creatinine
[redacted]	[redacted]
SPMS	Secondary Progressive Multiple Sclerosis
SUSAR	Suspected Unexpected Serious Adverse Reactions
TBL	total bilirubin level
[redacted]	[redacted]
TSQM	Treatment Satisfaction Questionnaire for Medication
ULN	upper limit of normal
UPV	unplanned visit
UTI	Urinary Tract Infection
WBC	white blood cell(s)

[redacted]

Glossary of terms

Assessment	A procedure used to generate data required by the study.
Biologic Samples	Biological specimen including, for example, blood (plasma, serum), saliva, tissue, urine, stool, etc. taken from a study participant.
Clinical Outcome Assessment (COA)	A measure that describes or reflects how a participant feels, functions, or survives.
Clinical Trial Team	A group of people responsible for the planning, execution and reporting of all clinical trial activities. Examples of team members include the Study Lead Medical Monitor Trial Statistician etc.
Coded data	Personal Data which has been de-identified by the investigative center team by replacing.
Cohort	A specific group of participants fulfilling certain criteria.
Discontinuation from study	Point/time when the participant permanently stops receiving the study treatment and further protocol required assessments or follow-up, for any reason. No specific request is made to stop the use of their samples.
Discontinuation from study treatment	Point/time when the participant permanently stops receiving the study treatment for any reason (prior to the planned completion of study drug administration, if any). Participant agrees to the other protocol required assessments including follow-up. No specific request is made to stop the use of their samples or data.
Dosage	Dose of the study treatment given to the participant in a time unit (e.g. 100 mg once a day, 75 mg twice a day).
Electronic Data Capture (EDC)	Electronic data capture (EDC) is the electronic acquisition of clinical study data using data collection systems, such as Webbased applications, interactive voice response systems and clinical laboratory interfaces. EDC includes the use of Electronic Case Report Forms (eCRFs) which are used to capture data transcribed from paper source forms data/documents used at the point of care.
Enrollment	Point/time of participant entry into the study at which informed consent must be obtained (i.e. prior to starting any of the procedures described in the protocol). The action of enrolling one or more participants.
Estimand	As defined in the ICH E9 (R1) addendum, estimand is a precise description of the treatment effect reflecting the clinical question posed by the trial objective. It summarizes at a population-level what the outcomes would be in the same participants under different treatment conditions being compared. Attributes of an estimand include the population, variable (or endpoint) and treatment of interest, as well as the specification of how the remaining intercurrent events are addressed and a population level summary for the variable.
Intercurrent events	Events occurring after treatment initiation that affect either the interpretation or the existence of the measurements associated with the clinical question of interest.
Investigational drug	The study drug whose properties are being tested in the study; this definition is consistent with US CFR 21 Section 312.3 and is synonymous with “investigational new drug” or “investigational medicinal product”.

[redacted]

Investigational treatment	All investigational drug(s) whose properties are being tested in the study as well as their associated treatment controls. This includes any placebos, any active controls, as well as approved drugs used outside of their indication/approved dosage or tested in a fixed combination. Investigational treatment generally does not include other treatments administered as concomitant background therapy required or allowed by the protocol when used within approved indication/dosage.
Medication number	A unique identifier on the label of each study drug package in studies that dispense study drug using an IRT system.
Off-site	Describes trial activities that are performed at remote location by an off-site healthcare professional, such as procedures performed at the participant's home.
Off-site healthcare professional (OHP)	A qualified healthcare professional, such as include those used in the study e.g. Nurse, Phlebotomist, and/or Physician, who performs certain protocol procedures for the participant in an off-site location such as a participant's home.
Part	A single component of a study which contains different objectives or populations within that single study. Common parts within a study are: a single dose part and a multiple dose part, or a part in Participants with established disease and in those with newly-diagnosed disease.
Participant	An individual (trial participant) who has consented to participate in this study. The term Participant may be used to describe either a healthy volunteer or a Patient. “Participant” terminology is used in the protocol whereas term “Subject” is used in data collection.
Participant number	A number assigned to each Participant who enrolls in the study. When combined with the center number, a unique identifier is created for each Participant in the study and should be used to identify the participant throughout the study for all data collected, sample labels, etc.
Patient-Reported Outcome (PRO)	Measurement based on a report that comes directly from the patient about the status of a participant's health condition without amendment or interpretation of the patient's report by a clinician or anyone else.
Period	A minor subdivision of the study timeline; divides phases into smaller functional segments such as screening, baseline, titration, washout, etc.
Randomization	The method of assigning trial participants to investigational drug using a random process.
Re-screening	If a participant fails the initial screening and is considered as a Screen Failure, he/she can be invited once for a new Screening visit after medical judgment and as specified by the protocol.
Remote	Describes any trial activities performed at a location that is not the investigative site where the investigator will conduct the trial, but is for example a home or another appropriate location.
Screen Failure	A Participant who is screened but is not treated.
Study completion	Point/time at which the Participant came in for a final evaluation visit or when study drug was discontinued whichever is later.
Study drug/treatment	Any drug (or combination of drugs) administered to the Participant as part of the required study procedures; includes investigational drug, active drug run-ins or background therapy.
Tele-visit	Procedures or communications conducted using technology such as telephone or video-conference, whereby the participant is not at the investigative site where the investigator will conduct the trial.

[redacted]

Variable	A measured value or assessed response that is determined in specific assessments and used in data analysis to evaluate the drug being tested in the study.
Withdrawal of consent (WoC)/ Opposition to use of data/biological samples	Withdrawal of consent from the study occurs when the participant explicitly requests to stop use of their data and biological samples (opposition to use data and biological samples) AND no longer wishes to receive study treatment, AND does not agree to further protocol required assessments. This request should be in writing (depending on local regulations) and recorded in the source documentation. Opposition to use data/biological samples occurs in the countries where collection and processing of personal data is justified by a different legal reason than consent.

[redacted]

Amendment 02

Rationale for Amendment

Although IgM and IgG are assessed at baseline, this does not indicate if participants' values were declining, were stable or were inclining when entering the trial. Collection of historical values will allow us to observe trends prior to initiating treatment with ofatumumab.

The purpose of the Amendment 2 is to amend the Schedule of Assessments to include:

Table 8-1: Addition of "Historical IgM and IgG" collection to the screening visit

- Footnote #25 added: Historical IgM and IgG results if available from pre-baseline of IV anti-CD20 treatment (one result pre-infusion) and then any testing done following IV anti-CD20 initiation. This should be recorded once for all participants enrolled or completed and may be collected at any visit.
- Footnote #14: Addition of the word "safety" in front of assessments

Section 8.4.5: Added "If available, historical IgM and IgG values that have been collected prior to screening visit, include one pre-anti-CD20 infusion value, should be recorded including the collection date. This should be recorded for all new, existing, and completed participants, and may be entered at any time-point during the study.

Section 12.5.2 under Clinical laboratory evaluations: Added "In addition, historical change of IgM and IgG will be summarized."

Section 15: Added reference "Agarwal S and Cunningham-Rundles C (2007) Assessment and clinical interpretation of reduced IgG values. Annals of allergy, asthma & immunology. 99(3): 281-283."

All relevent section(s) of the protocol are updated to reflect all changes.

Additionally, other administrative changes include the following:

- Under Table 8-3 (Severity of MS Relapse) removed "Byrne 2003"

Amendment 01

Rationale for Amendment

The purpose of Amendment 1 is to amend the inclusion and exclusion criteria.

Changes to the Inclusion criteria in protocol summary and section 5.1

- Inclusion #2: Increased age limit to 60 years of age.
- Inclusion #5: Removed "no more than 5 courses" limit and updated to at least 1 fully infused ocrelizumab IV infusion every 6 months
- Inclusion #7: Removed "Participants with CD19 B cells that are depleted to < 1%, as measured by the central laboratory"

Changes to the Exclusion criteria in protocol summary and section 5.2

- Exclusion# 1: Updated to include:
    a. Signs of MRI activity, defined as ≥ 2 active Gd+ T1 lesions, or any new or newly enlarging T2 lesions, documented within the past 6 months

[redacted]

- If a prior MRI within the last 6 months is not available, then new or newly enlarging T2 lesions should be considered "not documented" and the patient may continue screening

b. Documented relapse while on stable, previous aCD20 treatment.

- Relapses during the first 3 months of intravenous aCD20 therapy are allowable if the participant is then relapse-free for the 12 months following the relapse while on intravenous aCD20 therapy

c. Any signs of clinical worsening as measured by EDSS or any clinical measure documented within the last 6 month

- Exclusion #2b: Updated to include: if the Investigator believes this is related to therapy.
- Exclusion# 12: Updated to include: There is presently no contraindication for the use of an inactivated, viral-vector-, or mRNA based Sars-CoV-2 vaccine in patients who are immunocompromised. However, different Sars-CoV-2 vaccines may have various mechanisms of action and different associated potential risks. Please review local prescribing information of any specific Sars-CoV-2 vaccine and comply with local prescribing information requirements for specific contra-indications and special warnings and precautions for use
- Exclusion #20: was changed to: Patients unable or unwilling to undergo MRI scans with gadolinium
- [redacted]
- Section 2.1 Primary estimands attributes was updated to change endpoint to variable, and to include: **Population**: relapsing MS Participants currently treated with an infused aCD20 mAb, specifically ocrelizumab or rituximab, that have received at least 2 courses of therapy, that are switching due to reasons other than safety or efficacy. Further details about the population are provided in Section 5. **Intercurrent event**: Death or treatment/study discontinuation and **Summary measure**: Proportion of subjects achieving no change or a reduction in gadolinium enhancing lesions on MRI at Month 12.
- Section 3: Figure3-1 Study Design was updated from every 4 weeks to once monthly and throughout protocol
- Added Section 3.1: Remote Procedures
- Section 6.7.2: removed "Participants should remain at study site under observation for approximately 1 hour following dosing at Visit 1/BSL, where post-dose vital signs will be collected. A longer observation period may be required if these vital signs are not within reasonable limits of the participant's baseline values."
- Table 8-1: Added ECG at unscheduled visit and added footnote #24 Additional unplanned ECGs may be performed at the Investigator's discretion if clinically indicated.
- [redacted]
- [redacted]

[redacted]

- Table 8-1: Updated to removed [redacted] TSQM-9 from the screening visit
- [redacted]
- Section 8.1.3 removed "Participants who are randomized and fail to start study treatment, e.g. participants randomized in error, will be considered an early terminator. The reason for early termination should be recorded on the appropriate Case Report Form"
- Section 8.2 removed date of birth
- Section 8.3.1: added cervical spine, and added in the footnote #9 for clarity
- [redacted]
- Section 8.4.2: Added updated language and added Table 8.4 Local ECG Collection plan
- Section 8.4.3 Updated to include: At the baseline visit only, systolic and diastolic blood pressure should be measured 3 times.
- [redacted]
- Section 12.4: Revised from six to twelve months
- Section 12.4.1: Revised from 6 to 12 months
- Section 12.4.3: update to include: Missing data will be handled using non-responsive imputation regardless of intercurrent event.
- [redacted]
- Section 15: Additional references and throughout protocol
- Section 16.3.3: Added language "There is presently no contraindication for the use of an inactivated, viral-vector-, or mRNA based Sars-CoV-2 vaccine in patients who are immunocompromised. However, different Sars-CoV-2 vaccines may have various mechanisms of action and different associated potential risks. Please review local prescribing information of any specific Sars-CoV-2 vaccine and comply with local prescribing information requirements for specific contraindications and special warnings and precautions for use. Vaccination against Sars-CoV-2 should be considered on a case-by-case basis at the discretion of the treating physician taking into account the individual benefit-risk assessment and local vaccination recommendations."

All relevant section(s) of the protocol are updated to reflect all changes.

Additionally, other administrative changes include the following:

[redacted]

- Under List of Abbreviations: [redacted] removed "electronic" from the CSSR-S
- Updated Glossary Terms to reflect all changes in the protocol.

[redacted]

Protocol summary

**Protocol number**	COMB157GUS07
**Full Title**	A single-arm, prospective, multi-center study to explore maintained efficacy with ofatumumab therapy in patients with relapsing multiple sclerosis who discontinue intravenously delivered anti-CD20 monoclonal antibody (aCD20 mAb) therapy (OLIKOS)
**Brief title**	A single arm study evaluating the continued efficacy, safety and tolerability of ofatumumab in patients with relapsing multiple sclerosis who are transitioning from aCD20 mAb therapy
**Sponsor and Clinical Phase**	Novartis<br>Clinical Phase 3b
**Investigation type**	Drug
**Study type**	Interventional, Single-arm
**Purpose and rationale**	The objective of this study is to evaluate continued clinical efficacy, retention and satisfaction, as well as safety and tolerability for ofatumumab 20mg subcutaneous administered once monthly in participants with relapsing forms of multiple sclerosis who transition from aCD20 mAb therapy.
**Primary Objective(s)**	To explore maintenance of efficacy evaluated by MRI lesions at twelve months after initiating ofatumumab therapy vs baseline in participants with relapsing multiple sclerosis who are transitioning to ofatumumab from aCD20 mAb therapy (for reasons other than suboptimal response or certain treatment-emergent adverse events).
**Secondary Objectives**	Characterize participant retention, immune biomarkers, treatment satisfaction, and safety and tolerability at 6 and 12 months after transitioning to ofatumumab.
**Study design**	This is a single-arm, multicenter, prospective study in approximately 100 participants who are transitioning from aCD20 therapy to ofatumumab. This study consists of three parts: The 28 day screening period, the 12 month treatment period, plus a 30-day telephone safety follow-up and if required every 3 month post treatment safety follow-up visits until either another therapy is initiated or B cells are repleted, defined as a B cell concentration greater than the individual participant's baseline value prior to starting the IV anti-CD20 mAb or greater than the lower limit of normal. The minimum duration of the study for participants that complete the study is 14 months.
**Population**	Adult (ages 18 to 60) relapsing multiple sclerosis participants
**Key Inclusion criteria**	Participants eligible for inclusion in this study must meet **all** of the following criteria:<br>1. Written informed consent must be obtained before any assessment is performed.<br>2. Male or female participants aged 18 to 60 years (inclusive) at screening.<br>3. Diagnosis of relapsing MS (RMS) according to the 2017 Revised McDonald criteria including CIS, RRMS or SPMS with disease activity as defined by Lublin et al. 2014.<br>4. Disability status at Screening with an EDSS score of 0 to 5.5 (inclusive).

[redacted]

	5. Received at least 2 courses of intravenous aCD20 mAb (loading doses are considered 1 course):<br>• participants currently treated with ocrelizumab must have received (meet all three criteria below):<br>1. 2 fully infused initial 300 mg ocrelizumab iv infusions<br>2. At least 1 fully infused 600 mg ocrelizumab iv infusions every 6 months (+/- one month)<br>3. Last fully infused ocrelizumab dose must have occurred within 4-9 months prior to baseline.<br>• participants currently treated with rituximab must have received (meet both criteria below):<br>1. At least 2 fully infused courses of rituximab 500 mg – 1000 mg iv every 6 months (+/- one month).<br>a. Initial loading regimens of rituximab i.e. 500 mg – 1000 mg on day 1 and on day 15, are allowed but this is considered a single course and must be followed by additional infusion(s) every 6 months (+/- one month)<br>2. Last fully infused rituximab dose must have occurred within 4-9 months prior to baseline.<br>6. Participants currently stable on an intravenous aCD20 mAb therapy but are switching for reasons including physician/participant preference, access to commercial drug (e.g. insurance coverage issues) or for other logistical reasons (such as geographical relocation, travel, etc.) are eligible for this study.<br>7. Neurologically stable within 1 month prior to first study drug administration.<br>8. Must be able to use a smart device or have a care giver that can assist.
**Key Exclusion criteria**	Participants meeting any of the following criteria are not eligible for the inclusion of the study:<br>1. Participants that have demonstrated suboptimal response to aCD20 therapy to include:<br>a) Signs of MRI activity, defined as ≥ 2 active Gd+ T1 lesions, or any new or newly enlarging T2 lesions, documented within the last 6 months.<br>• If a prior MRI within the last 6 months is not available, then new or newly enlarging T2 lesions should be considered “not documented” and the patient may continue screening.<br>b) Documented relapse while on stable, previous aCD20 treatment<br>• Relapses during the first 3 months of intravenous aCD20 therapy are allowable if the participant is then relapse-free for 12 months following the relapse while on intravenous aCD20 therapy<br>c) Any signs of clinical worsening as measured by EDSS or any clinical measure, documented within the last 6 months.<br>2. Discontinuing aCD20 mAb therapy due to the following treatment-emergent adverse events:<br>a) Severe infusion-related reactions (Grade 3 or above).

[redacted]

	b) Recurrent infections defined as ≥ 2 severe infections or ≥ 3 respiratory infections or the need for ≥ 2 courses of antibiotics since starting aCD20 therapy, if the Investigator believes this is related to therapy.<br>c) Decreased IgG requiring treatment with Intravenous Immunoglobulin<br>3. Participants with primary progressive MS or SPMS without disease activity.<br>4. Participants meeting criteria for neuromyelitis optica.<br>5. Pregnant or nursing (lactating) women.<br>6. Women of child-bearing potential unless they are using highly effective forms of contraception during dosing and for at least 6 months after stopping study medication.<br>7. Participants with active chronic disease of the immune system other than MS or with immunodeficiency syndrome.<br>8. Participants with active systemic bacterial, viral or fungal infections, or known to have AIDS.<br>9. Participants with neurological symptoms consistent with PML or with confirmed PML.<br>10. Participants at risk of developing or having reactivation of syphilis or tuberculosis.<br>11. Participants at risk of developing or having reactivation of hepatitis.
**Study treatment**	Following initial loading regimen of 20 mg subcutaneous dosed on Days 1, 7 and 14, ofatumumab 20 mg subcutaneous injections once monthly.
**Treatment of interest**	The treatment of interest is ofatumumab and is the same as the study treatment. Following a loading dose regimen consisting of once daily doses on days 1, 7 and 14, ofatumumab will be given to participants once monthly starting at Month 1. All doses of ofatumumab will be given subcutaneously via an autoinjector at a dose of 20mg.
**Efficacy assessment**	• Magnetic Resonance Imaging (MRI)<br>▪ Gadolinium enhancing T1 lesions<br>▪ Gadolinium enhancing T1 upper cervical cord lesions<br>[redacted]
**Key safety assessments**	• Adverse events<br>• Laboratory evaluations (blood and urine)<br>• Columbia Suicide Severity Rating Scale (C-SSRS)<br>• Electrocardiogram (ECG)
**Other assessments**	• Treatment Retention<br>• Patient reported outcomes (PROs)<br>▪ Treatment Satisfaction Questionnaire for Medication (TSQM-9)<br>[redacted]
**Data analysis**	The primary aim of this study is to explore maintenance of efficacy evaluated by MRI lesions at Month 12 in participants with RMS who are transitioning to ofatumumab from aCD20 therapy.

[redacted]

	The primary endpoint will be no change or a reduction from Baseline (assessed at Screening Visit) in the number of gadolinium enhancing lesions observed by MRI at Month 12, a binary outcome (yes, no).<br>The number (and percentage) of participants with no change or a reduction will be presented. The 95% confidence interval for the proportion of participants with no change or a reduction will be calculated by using normal approximation method.<br>The above analyses will be conducted using the Full-Analysis-Set (FAS).<br>Sample size calculations were based on the proportion of participants with no change or a reduction from Baseline in the number of gadolinium-enhancing (GdE) MRI lesions 12 months after initiating ofatumumab therapy. Three precision estimates were considered for this study:<br>A sample size of 100 participants will provide an 8.5% precision (half-width of 95% confidence interval), a 7.8% precision, or a 7.0% precision corresponding to estimated proportions of 75%, 80%, and 85%, respectively, of participants achieving the primary endpoint.<br>For the purpose of earlier dissemination of data, an interim analysis will be performed on all enrolled participants who either meet the criteria of at least 6 months treatment or discontinued treatment prematurely. For this analysis, only primary variables, demographics, baseline characteristics, adverse events and serious adverse events will be summarized.<br>Since there will be no hypothesis testing involved, statistical adjustment for the interim analysis will not be made at the stage of final analyses of efficacy and safety.
**Key words**	Interventional, transitioning, clinical trial, effectiveness, safety, open-label, ofatumumab, relapsing multiple sclerosis, treatment satisfaction, patient reported outcomes

[redacted]

1 Introduction

1.1 Background

Multiple sclerosis (MS), one of the most common causes of neurological disability in young adults, is the prototypic acquired inflammatory demyelinating condition of the central nervous system (CNS), characterized by inflammation, demyelination and axonal/neuronal destruction, ultimately leading to severe disability in the majority of patients.

While traditional research has focused on the role of T cells for MS, pathogenic B cells are known to play an early role in the immune-mediated pathogenesis of MS (Archelos et al 2000, Frohman et al 2006, McFarland 2008). B cells, acting in concert with T cells have been found to be required for full disease expression (Genain and Hauser 1996), with essential functions in regulating immune response. B cells may contribute to disease pathogenesis by self-antigen presentation, serving as cellular adjuvants for cluster of differentiation (CD) 4+ T-cell activation (Bouaziz et al 2007) and by regulating T-cell function and inflammation via cytokine production (Lund 2008), in addition to producing autoantibodies. B-cells are present in chronic plaques, areas of demyelination, and in the cerebrospinal fluid of MS patients (Lehmann-Horn et al 2013). These observations have provided a new theoretical framework for the use of B cell based therapeutics in MS.

Selectively targeting B cells with anti-CD20 monoclonal antibodies has been proven highly effective at limiting disease activity in patients with relapsing forms of MS[1]. Rituximab and ocrelizumab are anti-CD20-directed cytolytic antibodies that have been investigated in MS. Clinical evidence from randomized, placebo-controlled Phase 2 studies with the chimeric mouse/human anti-CD20 monoclonal antibody (mAb) rituximab (Hauser et al 2008) and the humanized anti-CD20 mAb ocrelizumab (Kappos et al 2011) showed B-cell depletion by these agents lead to marked reductions in MRI-measured inflammatory activity in RMS participants. Recently, the efficacy of ocrelizumab was confirmed in two Phase 3 trials in participants with RMS (Hauser et al 2017). These studies showed that ocrelizumab significantly reduced relapse rates, reduced MRI disease activity and delayed the time to disability worsening versus interferon beta 1a over 2 years. Ocrelizumab was well tolerated with no major safety findings reported in these clinical trials.

While ocrelizumab has received FDA approval in 2017 (Ocrevus prescribing information; Hauser et al 2017) for the treatment of adult patients with relapsing or primary progressive forms of multiple sclerosis, rituximab has not. Nonetheless, rituximab has been utilized in the real-world setting as off-label treatment for MS patients in the US. Both rituximab and ocrelizumab are delivered in a clinical setting by intravenous (iv) infusion. Rituximab is given off-label in a range of doses. A typical rituximab treatment for RMS may consist of 500-1,000 mg infusions on Day 1 and Day 15 followed by a 1,000 mg infusion every six months. Ocrelizumab is initiated with 300 mg infusions on Day 1 and Day 15, and maintained with a 600 mg infusion every six months. The real-world utilization of both rituximab and ocrelizumab in the US is common and ocrelizumab use has continued to increase since its 2017 approval for relapsing and primary progressive forms of MS. However, the real world safety, tolerability and continued access to these anti-CD20 (aCD20) therapies for MS patients in the clinical setting is not fully elucidated. Furthermore, clinical evidence demonstrating whether MS patients can be transitioned from IV aCD20 antibody therapy to ofatumumab is not yet available.

[redacted]

Ofatumumab is a fully human aCD20 mAb that induces B cell lysis via complement-dependent cytotoxicity (CDC) and antibody-dependent cell-mediated cytotoxicity (ADCC) (Teeling et al 2006). Ofatumumab recognizes an epitope localized close to the cell membrane on the 2 extracellular domains of the CD20 molecule, N-proximal of the epitope for the anti–CD20mAb rituximab. Preclinical differentiation between aCD20 mAb suggests a favorable profile of ofatumumab including higher CDC (Pacheco-Fernandez et al 2018), direct lymph node targeting (Torres et al 2019) and potential sparing of marginal zone B cells, which are relevant for host defense (Theil et al 2019). The clinical correlates of these preclinical findings remain to be explored in Ph3b/4 studies. Ofatumumab has been evaluated in two Phase 2 studies in participants with RMS, one of which proved the positive effect on inflammatory lesions when given subcutaneously (Sorensen et al 2014, Bar-Or et al 2018). In the head-to-head phase III clinical trials, ASCLEPIOS I and II (COMB157G2301 and COMB157G2302, respectively), monthly subcutaneous ofatumumab 20mg demonstrated superiority over once daily oral teriflunomide 14mg in participants with relapsing forms of multiple sclerosis. Both studies met the primary endpoints where ofatumumab showed a highly significant and clinically meaningful reduction in the number of confirmed relapses, evaluated as the annualized relapse rate (ARR). Specifically, RMS participants on ofatumumab had a reduction in annualized relapse rate (ARR) by 50.5% (0.11 vs. 0.22) and 58.5% (0.10 vs. 0.25) compared to teriflunomide (both studies $p<0.001$) in ASCLEPIOS I and II studies respectively (Hauser et al 2019). Key secondary endpoints were also met, where ofatumumab showed a relative risk reduction of 34.4% in 3-month confirmed disability (CDW) ($p=0.002$) and 32.5% in 6-month CDW ($p=0.012$) versus teriflunomide in a pre-specified pooled analyses.

In the ASCLEPIOS studies, ofatumumab was subcutaneously administered via pre-filled syringe (PFS). In the APLIOS Phase II study (COMB157G2102), the autoinjector (AI) pen was compared with the pre-filled syringe (PFS) in a 12 week randomized open label multicenter study to demonstrate bioequivalence (Bar-Or et al 2020). The current study will utilize the ofatumumab AI pen to facilitate improved self- and home-administration and hence reduce the participant burden of treatment.

Notably, the ASCLEPIOS trials enrolled participants that were either treatment exposed or naive (~60% and 40%, respectively), but excluded participants with prior aCD20 mAb exposure (including rituximab and ocrelizumab) from study participation. Therefore, an evidence gap remains whether RMS participants discontinuing aCD20 mAb therapy might experience therapeutic benefit from treatment transition to ofatumumab. This study aims to explore whether ofatumumab is effective, safe, tolerable, and has better Participant satisfaction in these RMS participants.

1.2 Purpose

The purpose of the study is to complement the ofatumumab Phase 3 program by evaluating continued clinical efficacy, participant retention and satisfaction, as well as safety and tolerability for ofatumumab 20mg subcutaneous administered via the AI pen once monthly in participants with relapsing forms of MS who transition from aCD20 mAb therapy.

The primary outcome will be to explore maintenance of efficacy evaluated by MRI lesions (gadolinium enhancing T1 lesions) at 12 months after initiating ofatumumab therapy vs baseline in RMS participants transitioning to ofatumumab from aCD20 therapy (for reasons other than

[redacted]

suboptimal response or certain treatment-emergent adverse events). This phase 3b trial will also characterize participant retention, immune biomarkers, treatment satisfaction, and safety and tolerability at 6 and 12 months in participants transitioning to ofatumumab from an aCD20 mAb.

[redacted]

2 Objectives, endpoints and estimands

Table 2-1 Objectives and related endpoints

Objective(s)	Endpoint(s)
**Primary objective(s)**	**Endpoint(s) for primary objective(s)**
• Explore maintenance of efficacy of ofatumumab in participants with RMS transitioning from aCD20 therapy	• No change or a reduction from baseline in the number of gadolinium enhancing lesions observed by MRI at 12 months of ofatumumab treatment (yes, no)
**Secondary objective(s)**	**Endpoint(s) for secondary objective(s)**
• Characterize participants retention, immune biomarkers, treatment satisfaction, and safety and tolerability at 6 and 12 months after transitioning to ofatumumab	• Retention on ofatumumab treatment from baseline to Month 6, and to Month 12 (yes, no)<br>• Change from baseline in lymphocytes, including total CD19+ B cell counts and CD3+CD20+ T cell counts, obtained by fluorescence activated cell sorting (FACS) at Months 6 and 12<br>• C-SSRS at Months 6 and 12<br>• Treatment Satisfaction Questionnaire for Medication (TSQM-9) scores (including domains) at baseline vs Month 6 and vs Month 12<br>• Treatment-emergent adverse events
[redacted]	

[redacted]

2.1 Primary estimands

The primary clinical question of interest is whether ofatumumab can maintain efficacy in relapsing MS participants as measured by the presence of gadolinium enhancing lesions on MRI at 12 months after switching from an intravenous aCD20 mAb for reasons other than efficacy or safety.

The justification for the primary estimand is that it will capture whether or not participants can be maintained on subcutaneous ofatumumab after documented stable, effective treatment with an intravenous aCD20 mAb, a scenario that is a real life question in the MS community where continued access to an infusion suite may not be possible or desired.

The primary estimand is described by the following attributes:

- **Population**: relapsing MS Participants currently treated with an infused aCD20 mAb, specifically ocrelizumab or rituximab, that have received at least 2 courses of therapy, that are switching due to reasons other than safety or efficacy. Further details about the population are provided in Section 5.
- **Variable:** no change or a reduction in gadolinium enhancing lesions on MRI at Month 12 (yes/no).
- **Treatment of interest**: following a loading dose regimen consisting of once daily doses on days 1, 7 and 14, ofatumumab is given once monthly starting at Month 1. All doses of ofatumumab will be given subcutaneously via an autoinjector at a dose of 20mg. Further details about the investigational treatment are provided in Section 6.
- **Intercurrent event**: Death or treatment/study discontinuation.
- **Summary measure**: Proportion of subjects achieving no change or a reduction in gadolinium enhancing lesions on MRI at Month 12.

2.2 Secondary estimands

Not applicable

[redacted]

3 Study design

This is a single-arm, multi-center, prospective, study in approximately 100 participants with relapsing multiple sclerosis who have been previously treated with aCD20 mAb therapy and have received at least 2 consecutive courses of intravenously administered ocrelizumab or rituximab every 6 months and the last dose is within 4 to 9 months before Baseline/Day 1. In this study, participants may enroll only if discontinuing aCD20 therapy for reasons other than lack of efficacy or due to certain treatment-emergent adverse events. Reasons for switching may include but are not limited to physician/participant preference, access to commercial drug (e.g. insurance coverage issues) or for other logistical reasons (such as geographical relocation, travel, etc.) A total of approximately 100 eligible participants will receive open label ofatumumab 20 mg subcutaneous once monthly for 12 months following initial loading regimen of 20 mg subcutaneous doses on Days 1, 7 and 14.

Assessments will be done per the assessment schedules (Table 8-1 and Table 8-2).

For participants who discontinue study treatment prematurely for any reason before the end of the treatment phase, an End of Study visit must be performed within 7 days of study discontinuation.

Upon completing the study, participants may continue ofatumumab therapy on commercial drug.

Safety follow-up:

- The Safety follow-up (FU) period will be applicable for the following participants:
    - participants who complete the Treatment period on the study drug and do not continue on any MS DMT.
    - participants who prematurely discontinue study drug and do not continue on any MS DMT.
- All Safety FU visits must be scheduled relative to the End of Study (EOS) Visit.

Participants that do not continue into the ofatumumab commercial patient services hub within one month of the EOS Visit or that do not switch to another therapy, must continue into the safety FU phase, consisting of every 3 month visits including B cell monitoring until they are able to start on commercial ofatumumab or until they switch to another therapy or until their B cells are repleted defined as a B cell concentration greater than the individual participant's baseline value prior to starting the IV anti-CD20 mAb or greater than the lower limit of normal. All participants will have a safety follow-up phone call at 30 days post study.

A shortened follow-up time is acceptable if participants select different treatment during this period or show earlier repletion. If participants are not fully repleted by the 6 month post-trial safety follow-up visit, further follow up visits at a 3 monthly frequency are recommended until full repletion has occurred. (Table 8-2)

For participants completing the trial, the total duration of the trial will be a minimum of 14 months. This accounts for the 28 day screening period, the 12 month treatment period, and a 30 day telephone safety follow-up. Safety follow-up may be longer if the participant has not switched to another therapy post-trial and B cell repletion has not occurred.

[redacted]

Figure 3-1 Study design

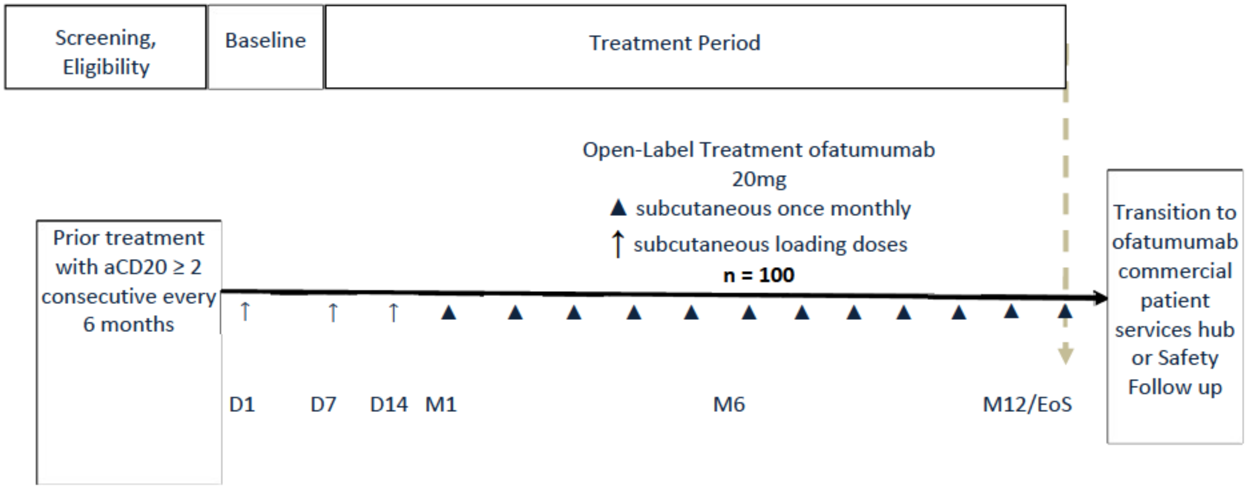

3.1 Remote procedures

At the investigator's direction and based on benefit-risk considerations of the participant's clinical condition, qualifying participants may be offered the option to have certain clinical trial procedures according to Table 8-1 Assessment schedule performed at the participants home. This is optional for all participants.

Procedures will be performed remotely under the oversight of the Investigator and study staff, who retains accountability for the oversight and all efficacy and safety decisions with delegation of tasks to the home nurse (OHP) provided by a third-party vendor sourced by Novartis. If the site wishes to use the home nurse (OHP) provided by Novartis this must be agreed and approved with Novartis before use.

In addition to procedures performed by the home nurse (OHP), the Investigator and study staff will perform certain procedures remotely using the sites own tele-visit platform. Novartis has the right to decide at any time what can and cannot be performed remotely.

On Day 7, 14 and Month 2, 3, 4, 5, 7, 8, 9, 10, and 11, the participant, caregiver and/or home nurse will administer 20mg of ofatumumab subcutaneous using an autoinjector pen. For females of childbearing potential, the participants, caregiver and/or home nurse will obtain a urine pregnancy test prior to administering study medication. Investigator and/or study staff will contact the participant remotely to access any adverse events, concomitant medications and answer any questions the participant may have. If needed due to the pandemic, epidemic or natural disaster participants can opt into having Month 1 and Month 6 completed remotely by the home nurse (with the investigator or sub investigator via tele visit). The home nurse will obtain a blood draw, vital signs, weight, urinalysis, [redacted], physical exam [redacted]. All participants will need to go on site for the month 6 MRI scan.

[redacted]

4 Rationale

4.1 Rationale for study design

The single arm non-comparative design has been selected due to feasibility of recruitment and time to study completion. The real-world varied dosing schedules associated with ocrelizumab and rituximab would not allow for standardization of a parallel arm. It is also designed to more closely reflect routine clinical setting and care, especially for RMS participants transitioning from intravenous aCD20 mAbs to ofatumumab. To further elucidate the effectiveness of transitioning to ofatumumab, additional data from participant registries or other data sources may be utilized.

Rationale of study population:

The study includes RMS participants who are transitioning from rituximab or ocrelizumab for reasons other than lack of efficacy (defined as documented relapse while on previous aCD20 treatment, or signs of MRI activity, defined as ≥ 2 active Gd+ T1 lesions, or any new or newly enlarging T2 lesions or change in EDSS) or due to certain treatment-emergent adverse events. These criteria have been implemented to further characterize the benefits of treatment with ofatumumab in a RMS population who already have achieved stable disease activity with other anti-CD20 mAbs which have demonstrated strong reductions in relapses and Gd T1 lesions and new and/or enlarging T2 lesions on MRI.

Exclusion of participants who have breakthrough disease with rituximab or ocrelizumab reduces the potential for enrolling participants who may not respond to aCD20 mAb therapy. Additional exclusion criteria, relating to certain treatment-emergent adverse events on rituximab or ocrelizumab, concomitant diseases, laboratory parameters, and previous medications help to ensure participants safety in the study.

Rationale of chosen endpoints:

The proposed study endpoints are widely accepted as clinically relevant and have been used in numerous pivotal clinical trials in relapsing MS. The primary endpoint for the study will be no change or a reduction in the number of gadolinium enhancing lesions observed by MRI at 12 months of ofatumumab treatment vs baseline. Key secondary endpoints will include participant retention, immune biomarkers, treatment satisfaction, and safety and tolerability at 6 and 12 months after transitioning to ofatumumab. In two large phase 3 studies, ofatumumab demonstrated a significant reduction in disease activity (reductions in relapses, Gd T1 lesions and new and/or enlarging T2 lesions) versus an active comparator.

This study aims to provide clinical evidence supplementing the phase 3 program by providing effectiveness and safety data of ofatumumab in a RMS participant population that is switching aCD20 treatment. This evidence is highly relevant for neurologists and treating healthcare practitioners in the real world setting. A planned analysis for the primary endpoint at six months will provide early and important data to the health care community about the effectiveness and safety of ofatumumab in participants transitioning from other aCD20 therapies.

[redacted]

4.2 Rationale for dose/regimen and duration of treatment

Ofatumumab dose

The dose regimen for ofatumumab for this study is a loading dose regimen of 20 mg at Day 1, Day 7 and Day 14, followed by a monthly maintenance dose regimen of 20 mg administered once monthly starting at Month 1.

This dose regimen is consistent with the USPI and the dose and regimen in the Phase 3 development program of ofatumumab in RMS.

Duration of treatment

Treatment duration within the study will be 12 months.

4.3 Rationale for choice of control drugs (comparator/placebo) or combination drugs

No comparator arm will be implemented.

4.4 Purpose and timing of interim analyses/design adaptations

The sponsor will conduct an interim analysis once 100% of enrolled participants have reached approximately 6 months of ofatumumab treatment, or discontinued within 6 months of enrollment, to provide early effectiveness data for transitioning to ofatumumab from previous aCD20 therapy. For this analysis, only primary variables, demographics, baseline characteristics, adverse events and serious adverse events will be summarized.

4.5 Risks and benefits

The risk to participants in this trial will be minimized by compliance with eligibility criteria, close clinical monitoring, avoidance of prohibited treatments, adherence to protocol requirements and Investigator guidance regarding specific safety areas. In addition, participants will receive the benefit of being systematically monitored over a 12 month period at regular follow-ups for efficacy and safety outcomes.

Clinical experience with ofatumumab in RMS participants has accumulated from four Phase 2 studies and the two Phase 3 clinical trials. Approximately 2000 RMS participants have been exposed to ofatumumab in total. No unexpected safety findings were observed in MS participants who received ofatumumab in the completed studies.

In the 48-week, placebo-controlled, cross-over study (cross-over at 24 weeks) of intravenous doses of ofatumumab up to 700 mg, adverse events reported more frequently on ofatumumab vs placebo included: rash, throat irritation, erythema, fatigue, viral infection and flushing (Sorensen et al 2014). In the placebo-controlled, dose-ranging study of ofatumumab administered at subcutaneous doses up to 60 mg every 4 weeks for up to 24 weeks, injection related reactions were observed more frequently in the overall ofatumumab group (Bar-Or et al 2018). A 12-week study performed to demonstrate bioequivalence between ofatumumab 20 mg administered subcutaneous via either pre-filled syringe or autoinjector pen exposed 284 RMS participants to study drug. This study also showed that adverse events and serious adverse events were overall low in ofatumumab-treated participants and there were no unexpected safety signals (Bar-Or 2020). In the ASCLEPIOS I and II studies, ofatumumab

[redacted]

demonstrated a favorable safety profile with no unexpected safety signals, and no imbalance in adverse events or serious adverse events, rates of infection (including serious infection) or malignancy when compared to teriflunomide. In the studies, systemic injection-related reactions were reported in 20.6% of participants on ofatumumab and in 15.3% of participants on teriflunomide. Nearly all (99%) reported systemic injection reactions were mild to moderate (Grade 1-2) and were generally limited to the first injection.

Ofatumumab subcutaneous has demonstrated profound suppression of new MRI lesion activity (≥ 90% versus placebo over Weeks 4-12) in relapsing MS participants in a Phase 2 study. In the Phase 3 studies, ofatumumab showed a highly significant and clinically meaningful reduction in the number of confirmed relapses, evaluated as the annualized relapse rate (ARR). Participants treated with ofatumumab had an ARR of 0.11 and 0.10 compared to teriflunomide (ARR of 0.22 and 0.25) in ASCLEPIOS I and II respectively, corresponding to a reduction in ARR by 50.5% and 58.8% with ofatumumab (p<0.001 in both studies). Ofatumumab showed highly significant suppression of both Gd+ T1 lesions and new or enlarging T2 lesions compared to teriflunomide, demonstrating a profound suppression of new inflammatory activity. Additionally, ofatumumab showed a relative risk reduction of 34.4% (p=0.002) in 3-month confirmed disability worsening (CDW[†]) and 32.5% (p=0.012) in 6-month CDW versus teriflunomide in pre-specified pooled analyses (Hauser et al 2019). Taken together, available information of relevant clinical and MRI outcomes supports the potential efficacy of ofatumumab in participants with relapsing MS.

In the study described here, RMS participants transitioning to ofatumumab, will have been previously exposed to a minimum of 2 of rituximab or ocrelizumab. While rituximab is not a currently an FDA-approved disease-modifying therapy for RMS, large retrospective observational studies suggest that rituximab safety and efficacy in a heterogeneous RMS population are consistent with randomized controlled trials (Salzer et al 2016; Hauser et al 2008). The most common types of AEs and SAEs were infections; infusion-related reactions occurred in 7.8% of infusions and most commonly during the first 3 infusions (Salzer et al 2016). In RRMS, rituxumab also demonstrated clinical efficacy in the form of a low ARR of (0.044), a low incidence of contrast-enhancing lesions (4.6%) and the median EDSS remained stable from baseline.

Ocrelizumab safety and efficacy has been previously published (Hauser et al 2017) demonstrating that infusion-related reactions occur in 34.3% of participants, serious infections occurred in 1.3% of participants and neoplasms occurred 0.5% of participants. Efficacy outcomes from the ocrelizumab pivotal studies demonstrated that the annualized relapse rate, risk of confirmed disability progression, and MRI lesions were significantly decreased compared to interferon beta-1a in the pivotal trials.

While RMS participants in this study must be discontinuing rituximab or ocrelizumab treatment for reasons other than safety, the potential safety risks associated with those therapies still exist. There is currently no information about whether the transition from aCD20 mAbs to ofatumumab can be performed safely with acceptable efficacy outcomes for RMS participants.

4.6 Rationale for Public Health Emergency mitigation procedures

During a Public Health emergency as declared by Local or Regional authorities i.e. pandemic, epidemic or natural disaster, mitigation procedures to ensure participant safety

[redacted]

and trial integrity are listed in relevant sections. Notification of the Public Health emergency should be discussed with Novartis prior to implementation of mitigation procedures, and permitted/approved by Local or Regional Health Authorities and Ethics Committees as appropriate.

5 Population

The study population will consist of adult participants with RMS fulfilling all the eligibility criteria listed below. The study is planned to be conducted in approximately 10-20 centers within the United States. It is aimed to enroll approximately 100 participants. Participants who enroll and prematurely discontinue study will not be replaced.

5.1 Inclusion criteria

Participants eligible for inclusion in this study must meet **all** of the following criteria:

1. Written informed consent must be obtained before any assessment is performed.
2. Male or female participants aged 18 to 60 years (inclusive) at screening.
3. Diagnosis of relapsing MS (RMS) according to the 2017 Revised McDonald criteria (Thompson et al. 2018), including CIS, RRMS or SPMS with disease activity as defined by (Lublin et al. 2014).
4. Disability status at Screening with an EDSS score of 0 to 5.5 (inclusive).
5. Received at least 2 courses of intravenous aCD20 mAb (loading doses are considered 1 course):
    - Participants currently treated with ocrelizumab must have received (meet all three criteria below):
        1. 2 fully infused initial 300 mg ocrelizumab iv infusions
        2. At least 1 fully infused 600 mg ocrelizumab iv infusions every 6 months (+/- one month)
        3. Last fully infused ocrelizumab dose must have occurred within 4-9 months prior to baseline
    - Participants currently treated with rituximab must have received (meet both criteria below):
        1. At least 2, fully infused courses of rituximab 500 mg – 1000 mg iv every 6 months (+/- one month).
            a. Initial loading regimens of rituximab i.e. 500 mg – 1000 mg on day 1 and on day 15, are allowed but this is consider a single course and must be followed by additional infusion(s) every 6 months (+/- one month)
        2. Last fully infused rituximab dose must have occurred within 4-9 months prior to baseline.

[redacted]

6. Participants discontinuing aCD20 therapy for reasons including, but not limited to: physician/participant preference, access to commercial drug (e.g. insurance coverage issues) or for other logistical reasons (such as geographical relocation, travel, etc.) are eligible for this study.
7. Neurologically stable within 1 month prior to first study drug administration.
8. Must be able to use a smart device or have a caregiver that can assist.

5.2 Exclusion criteria

Participants meeting any of the following criteria are not eligible for inclusion in this study:

1. Participants that have demonstrated suboptimal response to aCD20 therapy to include:
    a. Signs of MRI activity, defined as ≥ 2 active Gd+ T1 lesions, or any new or newly enlarging T2 lesions, documented within the past 6 months
        - If a prior MRI within the last 6 months is not available, then new or newly enlarging T2 lesions should be considered "not documented" and the patient may continue screening
    b. Documented relapse while on stable, previous aCD20 treatment.
        - Relapses during the first 3 months of intravenous aCD20 therapy are allowable if the participant is then relapse-free for the 12 months following the relapse while on intravenous aCD20 therapy
    c. Any signs of clinical worsening as measured by EDSS or any clinical measure documented within the last 6 months
2. Discontinuing aCD20 mAb therapy due to the following treatment-emergent adverse events:
    a. Severe infusion-related reactions (Grade 3 or above)
    b. Recurrent infections defined as ≥ 2 severe infections or ≥ 3 respiratory infections or the need for ≥ 2 courses of antibiotics since starting aCD20 therapy, if the Investigator believes this is related to therapy.
    c. Decreased IgG requiring treatment with Intravenous Immunoglobulin
3. Participants with primary progressive MS (Polman et al 2011) or SPMS without disease activity (Lublin et al 2014).
4. Participants meeting criteria for neuromyelitis optica (Wingerchuk et al 2015).
5. Pregnant or nursing (lactating) women, where pregnancy is defined as the state of a female after conception and until the termination of gestation, confirmed by a positive hCG laboratory test.
6. Women of child-bearing potential, defined as all women physiologically capable of becoming pregnant, unless they are using highly effective methods of contraception during dosing and for at least 6 months after stopping study medication. Highly effective contraception methods include:
    - Total abstinence (when this is in line with the preferred and usual lifestyle of the participant, if accepted by the local regulation). **NOTE:** Periodic abstinence (e.g., calendar, ovulation, symptothermal, post-ovulation methods) and withdrawal **ARE NOT** acceptable methods of contraception

[redacted]

- Female sterilization (have had surgical bilateral oophorectomy with or without hysterectomy), total hysterectomy or tubal ligation at least six weeks before taking investigational drug. In case of oophorectomy alone, only when the reproductive status of the woman has been confirmed by follow up hormone level assessment
- For female participants on the study, the vasectomized male partner should be the sole partner
- Use of oral, (estrogen and progesterone), injected or implanted hormonal methods of contraception or placement of an intrauterine device (IUD) or intrauterine system (IUS) or other forms of hormonal contraception that have comparable efficacy (failure rate <1%), for example hormone vaginal ring or transdermal hormone contraception. In case of use of oral contraception, women should have been stable on the same pill for a minimum of 3 months before taking investigational drug.

In case local regulations deviate from the contraception methods listed above, local regulations apply and will be described in the ICF.

Note: Women are considered post-menopausal and not of child bearing potential if they have had 12 months of natural (spontaneous) amenorrhea with an appropriate clinical profile (e.g. age appropriate, history of vasomotor symptoms) or have had surgical bilateral oophorectomy (with or without hysterectomy), total hysterectomy or tubal ligation at least six weeks. In the case of oophorectomy alone, only when the reproductive status of the woman has been confirmed by follow up hormone level assessment is she considered not of child-bearing potential.

7. Participants with active chronic disease (or stable but treated with immune therapy) of the immune system other than MS (e.g. rheumatoid arthritis, scleroderma, Sjögren's syndrome, Crohn's disease, ulcerative colitis, etc.) or with immunodeficiency syndrome (hereditary immune deficiency, drug-induced immune deficiency).
8. Participants with active systemic bacterial, viral or fungal infections, or known to have acquired immunodeficiency syndrome (AIDS).
9. Participants with neurological symptoms consistent with PML or with confirmed PML.
10. Participants at risk of developing or having reactivation of syphilis or tuberculosis (eg. Participants with known exposure to, or history of syphilis, or active or latent tuberculosis, even if previously treated). Testing for syphilis and tuberculosis will be done at Screening.

    Testing must be done by the central lab (for syphilis e.g. by positive rapid plasma reagin (RPR); for tuberculosis testing, QuantiFERON®-TB Gold test to assess participant's eligibility at Screening).

    NOTE:

    - participants with an indeterminate QuantiFERON®-TB Gold test may be enrolled if the repeat QuantiFERON®-TB Gold test is negative prior to baseline visit.
    - participants with positive QuantiFERON®-TB Gold test are not eligible.
11. Participants at risk of developing or having reactivation of hepatitis: Positive results at Screening for serological markers for hepatitis (H) A, B, C, and E indicating acute orpchronic infection:
    - anti-HA Immunoglobulin M (IgM)

[redacted]

- HBs Ag and/or anti-HBc IgM and/or HB virus deoxyribonucleic acid (DNA)
- anti-HBs negative and Anti-HBc positive
- anti-HC IgG (if positive IgG, HCV-RNA PCR will be performed and if negative, participant can be enrolled)
- anti-HE IgM (if positive IgG and/or IgM, perform HE-RNA PCR and if negative, participant can be enrolled)

NOTE: If the Investigator suspects false positive hepatitis serology results, such as an antibody pattern indicating acute hepatitis infection but no corresponding elevated liver enzymes and no signs or symptoms of liver disease, an infectious disease expert may be consulted. If the infectious disease expert finds no evidence of acute or chronic hepatitis infection and considers the serology results false positive and not clinically relevant, the Investigator must document (in source data and as a comment in the eCRF) that the serology results are considered false positive and may then enroll the participant.

12. Have received any live or live-attenuated vaccines (including for varicella-zoster virus or measles) within 4 weeks prior to first study drug administration.
    a. There is presently no contraindication for the use of an inactivated, viral-vector-, or mRNA based Sars-CoV-2 vaccine in patients who are immunocompromised. However, different Sars-CoV-2 vaccines may have various mechanisms of action and different associated potential risks. Please review local prescribing information of any specific Sars-CoV-2 vaccine and comply with local prescribing information requirements for specific contra-indications and special warnings and precautions for use
13. Have been treated with any of the medications listed below within the time specified

**Medication**	**Exclusionary if used within the timeframe specified below**
Intravenous, oral, intra-articular or intramuscular corticosteroids, adrenocorticotropic hormone	30 days prior to Screening MRI scan
Immunosuppressive, chemotherapeutic medications (e.g. natalizumab, mitoxantrone, cyclophosphamide, cladribine, S1P modulators)	2 years prior to first study drug administration
Mitoxantrone (with evidence of cardiotoxicity following treatment or cumulative life-time dose > 60mg/m^2)<br>Alemtuzumab<br>Lymphoid irradiation; bone marrow transplantation	Anytime

[redacted]

Other strongly immunosuppressive treatments (with effects potentially lasting over 6 months)<br>Ofatumumab<br>aCD20+ monoclonal antibodies in development (e.g. ublituximab or obinutuzumab)<br>Daclizumab	

14. Use of other investigational drugs at the time of enrollment (Screening) or within the prior 30 days, or five elimination half-lives, or until the expected pharmacodynamic effect has returned to baseline, whichever is longer.
15. History of malignancy of any organ system (other than basal cell carcinoma, in situ squamous cell carcinoma of skin, or in situ carcinoma of cervix or the uterus that have been radically treated e.g. completely excised with clear margins), within the past 5 years, regardless of whether or not there is evidence of local recurrence or metastases.
16. Any of the following conditions or treatments that may impact the safety of the participant:
    - History of, or current, significant cardiac disease including cardiac failure (NYHA functional class II-IV), myocardial infarction (within 6 months), unstable angina (within 6 months), transient ischemic attack (within 6 months), stroke, cardiac arrhythmias requiring treatment or uncontrolled arterial hypertension
    - Concomitant clinically significant cardiac arrhythmias, e.g. sustained ventricular tachycardia and clinically significant second or third degree AV block without a pacemaker on screening electrocardiogram (ECG)
    - History of or active severe respiratory disease, including Chronic Obstructive Pulmonary Disease, interstitial lung disease or pulmonary fibrosis
    - Participants with asthma requiring regular treatment with oral steroids
    - Severe hepatic impairment (Child-Pugh class C) or any chronic liver or biliary disease
    - Participants with severe renal impairment (Glomerular Filtration Rate < 30 ml/min/1.73 m2)
    - Any medically unstable condition as determined by the Investigator
17. Any of the following abnormal laboratory values as confirmed by the central laboratory prior to first study drug administration:
    - Total or conjugated bilirubin (BIL) greater than 3 times upper limit of normal (ULN) range, unless in the context of Gilbert’s syndrome
    - Alkaline phosphatase (ALP) greater than 5 times the ULN range
    - ALT between 1.5 and 5 times the ULN range and an active infection with hepatotropic viruses (Herpes simplex virus, Cytomegalovirus and Epstein-Barr Virus)
    - Serum IgG < 300mg/dL (according to central laboratory range)

[redacted]

- Any other clinically significant laboratory assessment as determined by the Investigator (e.g. significant anemia, neutropenia, thrombocytopenia, signs of impaired bone marrow function)

18. Participants with severe hypoproteinaemia e.g. in nephrotic syndrome.
19. Participants with any of the following neurologic/psychiatric disorders prior to first study drug administration:
    - Score “yes” on item 4 or item 5 of the Suicidal Ideation section of the Columbia-Suicide Severity Rating Scale (C-SSRS), if this ideation occurred in the past 6 months, or “yes” on any item of the Suicidal Behavior section, except for the “Non-Suicidal Self-Injurious Behavior” (item also included in the Suicidal Behavior section), if this behavior occurred in the past 2 years.
20. Participants unable or unwilling to undergo MRI scans with gadolinium.

6 Treatment

6.1 Study treatment

6.1.1 Investigational drug

Investigational drug will be provided in an autoinjector for subcutaneous administration containing 20 mg ofatumumab (20 mg/0.4 ml). Study drug will be supplied by Novartis.

6.1.1.1 Decentralized Clinical Trial Model

The study medication and all required clinical study supplies could possibly be distributed via direct-to-patient shipment utilizing an extension of the IND for compliance purposes in cases of a pandemic or a national and/or global emergency. Remote study visit options may be available for participants unable to travel to the clinic for their study visit only under these circumstances.

6.1.2 Additional study treatments

No other treatment beyond investigational drug are included in this trial.

6.1.3 Treatment arms/group

This is open label treatment study with one arm.

6.1.4 Treatment duration

The planned duration of treatment is 12 months. Participants may be discontinued from treatment earlier due to unacceptable adverse events, disease progression and/or at the discretion of the Investigator or the participant.

6.1.5 Post Trial Continuity

Participants who have completed the treatment period successfully, will be given the option to terminate from the study or will be referred to the ofatumumab commercial patient services hub for post-trial continuity of treatment programs, where the benefit risk is acceptable and

[redacted]

discussed with the participant and investigator. Participants who terminate prior to the end of the treatment period or who withdraw permanently due to ofatumumab related AE or SAE should follow up with their primary neurologist for continued treatment options as quickly as possible.

6.2 Other treatment(s)

6.2.1 Concomitant therapy

The Investigator should instruct the participant to notify the study site about any new medications he/she takes after study enrollment. All medications, procedures, and significant non-drug therapies (including physical therapy and blood transfusions) administered after the participant was enrolled into the study must be recorded on the appropriate Case Report Forms.

Each concomitant drug must be individually assessed against all exclusion criteria/prohibited medication. If in doubt, the Investigator should contact the Novartis medical monitor before enrolling a participant or allowing a new medication to be started. If the participant is already enrolled, contact Novartis to determine if the participant should continue participation in the study.

If a participant is receiving dalfampridine (Ampyra®/Fampyra®) concomitantly with study treatment, the participant should have been on a stable dose at least 30 days prior to first study drug administration and remain on that dose throughout the study wherever possible.

Premedication prior to subcutaneous injection

Premedication is not required. Premedication with acetaminophen and/or antihistamines (or equivalent) is optional and may be administered at the discretion of the Investigator. For the first injection of treatment period (Day 1), the addition of premedication with steroids (methylprednisolone 100 mg iv or equivalent) is an additional consideration. If Investigators choose to administer premedication, it should be administered 30 to 60 minutes prior to study drug injection.

Any administrations of premedication must be recorded in the appropriate CRF.

After the initial dose of study medication, participants may inject study medication at home at Day 7 and onwards if they have demonstrated the ability to self-inject (refer to Section 6.7.2). Based on the experience with the injection(s) administered at the site and with the use of any premedication, the Investigator will evaluate whether or not premedication (such as acetaminophen and/or antihistamines) should be used before injections administered at home. If premedication is prescribed, the study staff must ensure that the participant will receive a sufficient supply of premedication for home-use (i.e. to last at least until the next study office visit) and clear instructions (oral and written) about type and dose of premedication, and when to take it (i.e. 30-60 min before the injection). The Investigator will evaluate the need for premedication, or a change in the prescription, at each visit (scheduled and unscheduled) including at the monthly participant contacts during the study (contact should include specific questions about any injection-related symptoms and the use of premedication). If, based on the remote contacts, a change in pre-medication may be needed, the participant should be asked to return to the site for an unscheduled visit.

[redacted]

The participants must be informed (through the participant information and consent process) about the possibility that injection related reactions might occur despite use of premedications and about the possible symptoms of a systemic injection reaction and their management. Furthermore, participants must be informed that the use of steroid premedication may increase the occurrence of premedication related adverse events i.e. flushing, chest discomfort, hypertension, tachycardia, and abdominal pain. Participants must be reminded to always carry their Participant Card, which includes the Investigator and site telephone contact numbers in case of an emergency.

6.2.2 Prohibited medication

Use of the treatments displayed in Table 6-1 is NOT allowed in combination with study drug, due to increased risk of immunosuppression and confounding of efficacy evaluations.

Exclusionary medications for study eligibility are listed in the exclusion criteria (Section 5.2). Use of excluded medications is not allowed while the participant is on study medication.

Table 6-1 Prohibited medication

Medication	Action taken
Immunosuppressive/chemotherapeutic medications (including herbal) or procedures, including but not limited to cyclosporine, azathioprine, leflunomide, methotrexate, cyclophosphamide, mitoxantrone, lymphoid irradiation and hematopoietic stem cell transplantation	Discontinue study drug, increase vigilance regarding infections.<br>NOTE: Restarting study drug in participants exposed to these medications is not permitted.
Monoclonal antibodies targeting the immune system, including but not limited to natalizumab, alemtuzumab, daclizumab and B-cell depleting agents under investigation, such as but not limited to ublituximab and obinutuzumab	Discontinue study drug, increase vigilance regarding infections.<br>NOTE: Restarting study drug after exposure to B-cell depleting agents is not permitted. For others only after consultation with the Sponsor.
Any other immunomodulatory or disease-modifying MS treatment, including but not limited to fingolimod, interferon beta, glatiramer acetate, dimethyl fumarate, intravenous immunoglobulin, plasmapheresis or systemic corticosteroids (except for when given for MS relapse treatment as defined in Section 6.2.3)	Interrupt or discontinue study drug, increase vigilance regarding infections.<br>NOTE: Restarting study drug in participants exposed to these medications is not permitted.
Administration of any live or live-attenuated vaccine (including for measles) is prohibited while participants are exposed to study drug (long lasting effects of the study drugs should be taken into consideration)	They may be administered when participants are no longer exposed to study drug. Consider risk/benefit and follow local labels.

[redacted]

6.2.3 Recommended treatment of MS Relapse

The decision to treat MS relapses should be based on the Investigator's judgement and/or local clinical practice. If MS relapses require treatment, the standard treatment should consist of a short course of corticosteroids of 3-5 days and up to 1000mg methylprednisolone/day or equivalent on an inpatient or outpatient basis. Standard of care will be followed during treatment as per local clinical practice.

Taper with oral steroids is not permitted. Plasmapheresis may be used only if participant does not respond to standard treatment with corticosteroids.

Investigators should consider the added immunosuppressive effects of corticosteroid therapy and increase vigilance regarding infections during such treatment and in the weeks following administration.

Use of steroids for treatment of MS attack/relapse must be recorded on the Concomitant Medications eCRF. Please refer to restrictions for MRI in Section 8.3.1 concerning the use of steroids and performing the MRI.

6.3 Participant numbering, treatment assignment, randomization

6.3.1 Participant numbering

Each participant is identified in the study by a participant Number (Participant No.), that is assigned when the participant is first enrolled for screening and is retained as the primary identifier for the participant throughout his/her entire participation in the trial. The Participant No. consists of the Center Number (Center No.) (as assigned by Novartis to the investigative site) with a sequential participant number suffixed to it, so that each participant is numbered uniquely across the entire database. Upon signing the informed consent form, the Participant is assigned to the next sequential participant No. available by the Investigator. The Investigator or his/her staff will contact the IRT and provide the assigned participant study identification number along with the requested identifying information to register the participant into IRT. The site must use the eCRF with the matching participant number from the electronic data capture (EDC) system to enter data.

If an enrolled participant fails to be assigned to treatment for any reason, the IRT must be notified within 2 days that the participant was not assigned to treatment. The reason for not being assigned to treatment will be entered into the appropriate eCRFs. If the participant is re-screened at a later date, a new Participant number will be assigned.

6.3.2 Treatment assignment, randomization

No randomization will be performed in this study. All participants will receive a loading dose regimen of ofatumumab (OMB157) 20 mg subcutaneous on Day 1, Day 7 and Day 14 with maintenance doses once monthly starting at Month 1 for study duration of one year.

6.4 Treatment blinding

Not applicable.

[redacted]

6.5 Dose escalation and dose modification

Dose escalation and modifications are not permitted in this study.

6.5.1 Dose modifications

Not applicable.

6.5.2 Follow-up for toxicities

Not applicable.

6.5.3 Drug Interruptions

Conditions/events that may lead to study drug interruptions based on Investigator judgment and overall clinical assessment include:

- reported serious adverse event;
- emergency medical condition, unplanned hospitalization involving use of excluded concomitant medications;
- abnormal laboratory value(s) or abnormal test or examination result(s)

Should the participant interrupt the study drug for whatever reason, the re-start decision should be made on a case-by-case basis (refer to Section 6.7.2). Should the Investigator decide, after informing the Sponsor, to re-initiate treatment with study drug, depending on the duration of the interruption, the first dose at re-start may need to take place at the study site to ensure observation in a similar manner as on Day 1.

The reason for interruption of treatment and date of interruption should be appropriately documented in the source documents as well as in the appropriate eCRF.

6.6 Additional treatment guidance

6.6.1 Transition period from previous aCD20 DMT

Evidence-based recommendations on transitioning between DMTs are lacking. Hence, only general recommendations apply for sequential administration of immunosuppressive therapies. The risk of additive immune system effects when switching from drugs with immune effects such as rituximab or ocrelizumab must be considered and balanced with the time to transition to ofatumumab.

For the purpose of this study, we define the transitioning period as the time between the last aCD20 mAb therapy infusion and starting ofatumumab treatment. Participants should initiate ofatumumab within 9 months of the last aCD20 infusion but no sooner than 4 months after the last aCD20 infusion.

During this transition period, the participant may not receive any other DMT for the treatment of MS.

The transition period should be documented in the appropriate eCRF and any AEs reported during the procedure must be recorded on the AE eCRF.

[redacted]

6.6.2 Treatment compliance

The Investigator must promote compliance by instructing the participant to take the study drug exactly as prescribed and by stating that compliance is necessary for the participant's safety and the validity of the study. The participant must also be instructed to contact the Investigator if he/she is unable for any reason to take the study drug as prescribed. Compliance will be assessed by the Investigator and/or study personnel at each visit. Participant compliance should be at least 80%. The Investigator and/or study personnel will counsel the participant if compliance is below 80%. Study drug accountability will also be determined by the site monitor while performing routine site visits and at the completion of the study. This information should be captured in the source document at each visit. All study drug dispensed and returned must be recorded in the Drug Accountability Log.

6.6.3 Emergency breaking of assigned treatment code

Emergency breaking of assigned treatment code is not applicable to this study as this is an open-label study.

6.7 Preparation and dispensation

Each study site will be supplied with study drug in packaging as described under investigational drug section.

A unique medication number is printed on the study medication label. Investigator staff will identify the study medication kits to dispense to the participant by contacting the IRT and obtaining the medication number(s). The study medication has a 2-part label (base plus tear-off label). Immediately before dispensing the medication kit to the participant, site personnel will detach the outer part of the label from the packaging and affix it to the source document.

6.7.1.1 Handling of study treatment

Study drug must be received by a designated person at the study site, handled and stored safely and properly and kept in a secured location to which only the Investigator and designated site personnel have access. Upon receipt, all study drug must be stored according to the instructions specified on the labels and in the Investigator's Brochure. Clinical supplies are to be dispensed only in accordance with the protocol. Technical complaints are to be reported to the respective Novartis CO Quality Assurance.

The Investigator must maintain an accurate record of the shipment and dispensing of study drug in a drug accountability log. Monitoring of drug accountability will be performed by field monitors during site visits or remotely and at the completion of the trial. Participants will be asked to return all unused study drug and packaging at the end of the study or at the time of discontinuation of treatment.

At the conclusion of the study, and as appropriate during the course of the study, the Investigator will return all unused study drug, packaging, drug labels, and a copy of the completed drug accountability log according to the instructions provided by Novartis or its agents.

[redacted]

6.7.2 Instruction for prescribing and taking study treatment

All medication for the duration of the study will be provided by Novartis. All eligible Participants will receive ofatumumab open label treatment. The study drug (ofatumumab injection) will be administered starting at "Visit 1 (Baseline/Day 1)". Drug will then be dispensed at scheduled visits throughout the treatment period. Starting at Month 1, the subcutaneous injections should be administered at monthly intervals (+/- 3 days).

At Visit 1/BSL, participants will receive the subcutaneous injection at the study site. Site personnel will provide training to the participants and view training video on the correct procedure for self- administration of the subcutaneous injections. The participant or a caregiver may inject the study medication under supervision of the study staff. Documentation of injection procedure understanding by the participant and/or the caregiver must be documented in the source document.

Site staff should remove the study treatment from the refrigerator and allow the AI pen to reach room temperature in their unopened box (approximately 15-30 minutes) before self-injection by the participant. Used syringes should be disposed of immediately after use in a sharps container.

A different body site (front of thighs, lower abdomen) should be chosen each time a dose is administered to reduce the risk of an injection-site reaction; investigator/qualified site staff/caregiver can also inject the study treatment in the outer upper arms. Each new injection should be given at least one inch from the previously used site. If administration is in the abdomen, the 2-inch area around navel should be avoided. Study treatment should also not be injected into areas where the skin is tender, bruised, red, or hard, or where the participant has scars or stretch marks.

Following Visit 1, sufficient study drug will be supplied to the participant to cover all home administrations. Participants may inject the study medication at home, if they demonstrated ability to self-administer injections. If a participant is unable/unwilling to self-administer injections, another individual (e.g. partner, caregiver, relative or a healthcare professional) may perform home-administration who has accompanied the participant to the site and has been trained on and demonstrated ability to correctly administer the subcutaneous injections. The participant may also continue to have injections at the site if this is the participant's preference. Participants with severe neurological deficits should not self-administer injections.

If a participant is unable to travel to the investigative site for their study visit, shipment of study treatment from the site to the participant's home may be available in cases of a pandemic or a national and/or global emergency.

Doses on day 7 (dose #2) and day 14 (dose #3) should be administered +/- 1 day. If the dose on day 7 (dose #2) is missed, the dose due on day 14 (dose #3) should be adjusted to 7 days after dose #2 was given.

If a monthly injection of ofatumumab is missed, it should be administered as soon as possible without waiting until the next scheduled dose. Subsequent doses should be administered at the recommended intervals.

Participants will be instructed to contact the investigator/site staff prior to self-administration at home if they are experiencing any AE/SAEs or have any concerns. Participants will record

[redacted]

the dates and time of all in office and at-home study treatment doses using the participant smart device through the Engage app (Clinical Ink). Provisional devices are available for participants that do not have a smart device and/or refuses to use their own.

Participants will be provided with the following items to facilitate the at-home injections of study treatment: an insulated bag with cool gel packs to transport the study treatment from the site to the participant's home; alcohol swabs and gauze pads; a sharps container for immediate disposal of the used syringes; monthly home urine pregnancy test and a copy of the instructions for home administration use leaflet will be provided which includes detailed information, precautions and instructions for administering subcutaneous injections using the AI pen. This information should be reviewed with the participant (and his/her partner/relative as applicable) to ensure that they understand the correct procedure for self/home administration.

All kits of study medication assigned by the IRT will be databased in the IRT system.

7 Informed consent procedures

Eligible participants may only be included in the study after providing (witnessed, where required by law or regulation), IRB/IEC-approved informed consent.

If applicable, in cases where the participant's representative(s) gives consent (if allowed according to local requirements), the participant must be informed about the study to the extent possible given his/her understanding. If the participant is capable of doing so, he/she must indicate agreement by personally signing and dating the written informed consent document.

Informed consent must be obtained before conducting any study-specific procedures (e.g. all of the procedures described in the protocol). The process of obtaining informed consent must be documented in the participant source documents.

Novartis will provide to Investigators in a separate document a proposed informed consent form that complies with the ICH GCP guidelines and regulatory requirements and is considered appropriate for this study. Any changes to the proposed consent form suggested by the Investigator must be agreed by Novartis before submission to the IRB/IEC.

Information about common side effects already known about the investigational drug can be found in the Investigator's Brochure (IB). This information will be included in the participant informed consent and should be discussed with the participant during the study as needed. Any new information regarding the safety profile of the investigational drug that is identified between IB updates will be communicated as appropriate, for example, via an Investigator notification or an aggregate safety finding. New information might require an update to the informed consent and then must be discussed with the participant.

Women of child-bearing potential must be informed that taking the study treatment may involve unknown risks to the fetus if pregnancy were to occur during the study and agree that in order to participate in the study they must adhere to the contraception requirements.

A copy of the approved version of all consent forms must be provided to Novartis after IRB/IEC approval.

[redacted]

8 Visit schedule and assessments

Assessment schedule (Table 8-1) lists all of the assessments and indicates with an "X" when they are performed. Assessments that will only be reported in the source documentation are marked with an “S”. All data obtained from these assessments must be supported in the participant’s source documentation.

Participants should be seen for all visits/assessments as outlined in the assessment schedule (Table 8-1) or as close to the designated day/time as possible, with an allowed visit window of ±1 day for Days 7, 14 and an allowed visit window of ± 3 days for Months 1, 2, 3, 4, 5, 6 7, 8, 9, 10, 11, 12 and an allowed visit window of ±7 days for 30 day telephone safety follow-up. Missed or rescheduled visits should not lead to automatic discontinuation. In case a visit is performed outside of the schedule, subsequent visits shall be performed in keeping with the original visit schedule. In addition to the scheduled visits, participants may have unscheduled visits due to a MS relapse, an acute illness of undetermined cause, for other reasons, or at the discretion of the Investigator. Data collected during unscheduled visits will be recorded in the unscheduled visits CRF. Eligible participants may start study treatment once it is confirmed that they meet all inclusion criteria and none of the exclusion criteria.

Participants who prematurely discontinue the study for any reason should be scheduled for the EOS visit within 7 days and then enter the Safety Follow-up period according to the schedule in Table 8-2. At this final visit, all dispensed investigational product should be reconciled, and the adverse event and concomitant medications should be recorded on the CRF.

[redacted]

Table 8-1 Assessment Schedule

Period[1]	Screening / BSL		Treatment Period													End of Treatment /Study	Follow-up	Unplanned
**Visit Name**	**Screening**	**BSL V1**	**V2**	**V3**	**V4**	**V5**	**V6**	**V7**	**V8**	**V9**	**V10**	**V11**	**V12**	**V13**	**V14**	**EOT/EOS V15[5]**	**Safety**	**Unscheduled Visit**
Visit Day (D)/Month (M)	-28 Days	D1	D7	D14	M1	M2	M3	M4	M5	M6	M7	M8	M9	M10	M11	M12	30-Day Tel.	UPV
Visit Window Days (d)			±1d	±1d	±3d	±3d	±3d	±3d	±3d	±3d	±3d	±3d	±3d	±3d	±3d	±3d	±7d	
Obtain Informed Consent	X																	
Inclusion/ exclusion criteria	X	X[12]																
Demography	X																	
Medical History	X																	
MS disease/ Treatment History[6]	X																	
Historical IgM and IgG	X[25]																	
Physical Exam	S	S[12]			S					S						S		S
Vital Signs	X	X[12,13]			X[13]					X[13]						X[13]		X
Height	X																	
Weight	X	X[12]			X					X						X		
ECG	X	X[3]														X		X[24]
MRI[9]	X[10]									X						X[11]		
[redacted]																		
Chemistry[12]	X[16]	X[12]			X					X						X		X

[redacted]

Period[1]	Screening / BSL		Treatment Period													End of Treatment /Study	Follow-up	Unplanned
**Visit Name**	**Screening**	**BSL V1**	**V2**	**V3**	**V4**	**V5**	**V6**	**V7**	**V8**	**V9**	**V10**	**V11**	**V12**	**V13**	**V14**	**EOT/EOS V15[5]**	**Safety**	**Unscheduled Visit**
Visit Day (D)/Month (M)	-28 Days	D1	D7	D14	M1	M2	M3	M4	M5	M6	M7	M8	M9	M10	M11	M12	30-Day Tel.	UPV
Visit Window Days (d)			±1d	±1d	±3d	±3d	±3d	±3d	±3d	±3d	±3d	±3d	±3d	±3d	±3d	±3d	±7d	
Hematology	X[16]	X[12]			X					X						X		X
Sample for CD19+ B-cells and CD3+CD20+ T-cells	X[16]	X[12]			X					X						X		X
Total IgG and IgM levels	X[16]	X			X					X						X		X
[redacted]																		
Hepatitis A, B, C and E	S[16]																	
Syphilis	S[16]																	
Tuberculosis	S[16]																	
HIV	S[16]																	
Pregnancy Test (serum)	S[16]				S					S						S		S
Pregnancy Test (urine)[17]		S[12]				S	S	S	S		S	S	S	S	S			S
Dipstick urinalysis[21]	X	X			X					X						X		X
IRT Call	S	S[12]			S					S						S		

[redacted]

**Period**[1]	**Screening / BSL**		**Treatment Period**													**End of Treatment /Study**	**Follow-up**	**Unplanned**
**Visit Name**	**Screening**	**BSL V1**	**V2**	**V3**	**V4**	**V5**	**V6**	**V7**	**V8**	**V9**	**V10**	**V11**	**V12**	**V13**	**V14**	**EOT/EOS V15**[5]	**Safety**	**Unscheduled Visit**
Visit Day (D)/Month (M)	-28 Days	D1	D7	D14	M1	M2	M3	M4	M5	M6	M7	M8	M9	M10	M11	M12	30-Day Tel.	UPV
Visit Window Days (d)			±1d	±1d	±3d	±3d	±3d	±3d	±3d	±3d	±3d	±3d	±3d	±3d	±3d	±3d	±7d	
Self-injection Training (Participant and/or caregiver)		S																
Study drug self-injection (at Home)[8]			X	X		X	X	X	X		X	X	X	X	X			
Administration of study treatment at site[22]		X			X					X						X		
Drug Accountability		S			S					S						S		
Dispensation of study drug [2,4,7,12,13,14]		S [2,4,7 12,13]			S[14]					S[14]						S[14]		S[14]
Dosage Administration Record		X			X					X						X		
Concomitant Medications[8]	X	X[12]	X	X	X	X	X	X	X	X	X	X	X	X	X	X	X	X
Adverse Events/ Serious Adverse Events[8]	X	X[12]	X	X	X	X	X	X	X	X	X	X	X	X	X	X	X	X
[redacted]																		
C-SSRS	X[12]				X					X						X		X

[redacted]

Period[1]	Screening / BSL		Treatment Period													End of Treatment /Study	Follow-up	Unplanned
**Visit Name**	**Screening**	**BSL V1**	**V2**	**V3**	**V4**	**V5**	**V6**	**V7**	**V8**	**V9**	**V10**	**V11**	**V12**	**V13**	**V14**	**EOT/EOS V15[5]**	**Safety**	**Unscheduled Visit**
Visit Day (D)/Month (M)	-28 Days	D1	D7	D14	M1	M2	M3	M4	M5	M6	M7	M8	M9	M10	M11	M12	30-Day Tel.	UPV
Visit Window Days (d)			±1d	±1d	±3d	±3d	±3d	±3d	±3d	±3d	±3d	±3d	±3d	±3d	±3d	±3d	±7d	
TSQM-9[13]		X[12]			X					X						X		X
[redacted]																		
Thank You Letter		S														S		
[redacted]																		
Health Status Telephone Call[8]			S[8]	S[8]		S[8]	S[8]	S[8]	S[8]		S[8]	S[8]	S[8]	S[8]	S[8]		S[8]	

X = assessment to be recorded in the clinical database or received electronically from a vendor

S = assessment to be recorded in the source documentation only

1. Visit structure given for internal programming purpose only.
2. All baseline assessments are to be performed prior to dispensing/dosing study drug.
3. ECG to be performed pre-dose on BSL (Day 1).
4. Enrollment (Day1/BSL) first dose (usually expected on the same day). If first dose occurs on a different day after enrollment, the day of the first dose should be considered as Day 1.
5. EOS visit will be required for all participants (participants who permanently discontinue study drug and participants who complete the study).
6. MS history including number of relapses in previous year and number of relapses in previous two years, all infusion reactions on previous therapy and the reason for switching from previous intravenous aCD20 must be captured.
7. Participant training on study requirements including study drug administration using the autoinjector, ePRO, functionality and use.
8. Remote contact with participants by site staff around the time of administration of ofatumumab self/home-injection. The contact should query about any new or worsening symptoms warranting an unscheduled visit, MS relapse compliance with study treatment, injection reactions, compliance with contraception requirements (when applicable). Method of contact can be via telephone, email or text message depending on the preference of each participant.

[redacted]

Period[1]	Screening / BSL		Treatment Period													End of Treatment /Study	Follow-up	Unplanned
Visit Name	Screening	BSL V1	V2	V3	V4	V5	V6	V7	V8	V9	V10	V11	V12	V13	V14	EOT/EOS V15[5]	Safety	Unscheduled Visit
Visit Day (D)/Month (M)	-28 Days	D1	D7	D14	M1	M2	M3	M4	M5	M6	M7	M8	M9	M10	M11	M12	30-Day Tel.	UPV
Visit Window Days (d)			±1d	±1d	±3d	±3d	±3d	±3d	±3d	±3d	±3d	±3d	±3d	±3d	±3d	±3d	±7d	

9. Restrictions may apply to MRI concerning steroid use, please refer to Section 8.3.4. Scans of the brain and cervical spine are needed.

10. Screening MRI should be completed once all other screening eligibility assessments have been completed and confirmed to meet eligibility criteria.

11. MRI scan at the EOS/EOT visit is required.

12. All assessments to be completed prior to baseline visit/first study drug administration.

13. Vital signs should be obtained 30-60(+/- 10min) minutes before the subcutaneous injection (study drug). If premedication is required, the vital signs should be taken prior to premedication administration. At the baseline visit only, systolic and diastolic blood pressure should be measured 3 times.

14. If the scheduled visit coincides with the day that study drug administration is scheduled, the participant should be instructed not to administer the injection before coming to the site so that the safety assessments can be completed prior to study drug administration unless the scheduled visit is outside of the 3 day window for drug administration.

[redacted]

16. Labs must be drawn to allow adequate time for results to be obtained before first study drug administration to ensure participant's eligibility.

17. Participant's contraception status must be reviewed and documented to ensure method of contraception continues to be appropriate per protocol requirement for highly effective contraception. Pregnancy test is conducted on female participants only. Urine pregnancy tests will be conducted at all scheduled visits as indicated in Table 8-1 . The participant must contact the Investigator immediately in the case of a positive test for confirmatory testing at the Investigator's discretion.

[redacted]

21. Urine dipstick: If positive send full urinalysis to central laboratory.

22. Site staff must observe the participant /caregiver administer study drug at Mo. 1 and Mo. 6 after all assessments have been completed with the exception of the MRI at Mo. 6.

24. Additional unplanned ECGs may be performed at the Investigator's discretion if clinically indicated.

25. Historical IgM and IgG results if available from pre-baseline of IV anti-CD20 treatment (one result pre-infusion) and then any testing done following IV anti-CD20 initiation. This should be recorded once for all participants enrolled or completed and may be collected at any visit.

[redacted]

Table 8-2 Assessment Schedule, Safety Follow-up

Period[1]	Post-Treatment Follow-Up			
Visit Name	Safety Follow-up Visit 1	Safety Follow-up Visit 2		Additional Follow-up Visits[2]
Visit (Month)	EOS + 3 Months	EOS + 6 Months		Every 3 months
Visit Window (Day)	±14	±14		±14
[redacted]				
Physical Exam[,5]	S	S		S
Vital Signs (including weight)	X	X		X
Routine lab samples including urine	X	X		X
Sample for CD19+ B-cells and CD3+CD20+ T-cells	X	X		X
Total IgG, IgM levels	X	X		X
[redacted]				
Urine Pregnancy Test[3,5]	S	S		S
Contraception Status[4,5]	S	S		S
Concomitant medications[6]	X	X		X
Adverse Events	X	X		X

[X] Assessment to be recorded in the clinical database or received electronically from a vendor
[1] Visit structure given for internal programming purpose only
[2] As needed for the Participants requiring prolonged B-cell plasma level monitoring
[3] Pregnancy test is conducted on female Participants only. Urine pregnancy tests will be conducted at all scheduled visits as indicated in Table 8-2. The Participant must contact the Investigator immediately in the case of a positive test for confirmatory testing at the Investigator's discretion.
[4] Participant's contraception status must be reviewed and documented to ensure method of contraception continues to be appropriate per protocol requirement for highly effective contraception.
[5] Assessments captured as source data
[6] Including corticosteroids used to treat MS relapse; any newly started MS treatment as applicable.

[redacted]

8.1 Screening

A participant who enters screening but is determined not to be eligible will be considered a screen failure. The Investigator may consider re-screening the participant at a later time if he/she believes that the participant's condition has changed and they may potentially be eligible. In this case, a new participant number will be allocated to the participant and he/she will need to re-perform all screening procedures with the exception of MRI. The original MRI may be used during rescreening if the rescreening occurs within 3 months of the original MRI date. A participant may be re-screened only once. A minimum of 24 hours must elapse between screen failure and re-screening. If a participant is re-screened, a new ICF must be obtained prior to re-screening the participant and re-screening must be documented in the participant's source documents.

8.1.1 Eligibility screening

8.1.1.1 Hepatitis screen, HIV screen

All participants will be screened for Hepatitis B surface antigen (HBsAg) and, if standard local practice, Hepatitis B core antigen (HBcAg). Screening for Hepatitis C will be based in HCV antibodies and if positive, HCV RNA levels should be determined.

Evaluation for HIV seropositivity will be performed, and, if positive, confirmation by a second technique available at the laboratory site e.g. Western blot. Appropriate counseling will be made available by the Investigator in the event of a positive confirmatory test. Notification of state and federal authorities, as required by law, will be the responsibility of the Investigator.

8.1.2 Site contact with participants during self/home drug administration

Site personnel will make remote contact with participants starting at Day 7 and Day 14 and then on a monthly basis (months 2, 3, 4, 5, 7, 8, 9, 10, and 11) in between scheduled site visits starting at Month 1 and continuing for the remainder of the study. The contact should take place around time of the monthly subcutaneous self/home-administration to query about any new or worsening symptoms warranting an unscheduled visit, compliance with study treatment, injection reactions, and compliance with contraception requirements when applicable. The method of contact with each participant can be the personal preference of the individual between telephone contacts, email contact or text messages, however, the site staff must be able to provide suitable source documentation of each contact regardless of the method of contact.

8.1.3 Information to be collected on screening failures

Participants who sign an informed consent form and are subsequently found to be ineligible prior to treatment or fail to start the treatment for any reason will be considered a screen failure. The reason for screen failure should be recorded on the appropriate Case Report Form. The demographic information, informed consent, and Inclusion/Exclusion pages must also be completed for screen failure participants. No other data will be entered into the clinical database for participants who are screen failures, unless the participant experienced a serious adverse event during the screening phase (Section 10.1.3).

.

[redacted]

8.2 Participant demographics/other baseline characteristics

Participant demographic data and baseline characteristics to be collected on the participants include, age, sex, race and ethnicity. Relevant medical history/current medical condition data includes data collected up to the point in which informed consent is signed. Where possible, diagnoses, and not symptoms will be recorded. Investigators will have the discretion to record abnormal test findings on the CRF capturing medical history whenever in their judgment, the test abnormality occurred prior to the informed consent signature.

MS disease history (including date of onset and diagnosis, number of previous MS relapses), previous MS treatment and employment status will also be collected on the corresponding CRFs.

Reason for switching from previous intravenous aCD20 must be captured on the CRF i.e. Participant convenience, insurance issues, mild-moderate infusion reactions, etc.

8.3 Efficacy

The primary objective of this study is to evaluate the maintenance of efficacy of ofatumumab in participants with RMS transitioning from intravenous aCD20 mAb therapy, as assessed by change or reduction in the number of gadolinium enhancing lesions observed by MRI at 12 months of ofatumumab treatment versus study screening. The secondary objective of this study is to characterize participant retention, immune biomarkers, treatment satisfaction, as well as safety and tolerability at 6 and 12 months after transitioning to ofatumumab. The study includes the following efficacy assessments conducted at visits as shown in Table 8-1:

Imaging

- Magnetic Resonance Imaging

Clinical

[redacted]

Laboratory

[redacted]

Patient Reported Outcomes

- Treatment Satisfaction Questionnaire for Medication - 9 (TSQM-9)

[redacted]

An overview of each of these assessments is provided in the sections below and the details of these assessments will be provided in the site manuals.

8.3.1 Magnetic Resonance Imaging (MRI)

All participants will undergo MRI scanning of the brain and cervical spine according to the schedules in Table 8-1.

[redacted]

MRI scans will be read by the central MRI reading center. Prior to the start of the study, MRI technician from each center will receive an MRI Manual, outlining technical implementation, image quality requirements and MRI administrative procedures.

The site will be asked to program the MRI scanner that is designated for evaluation of the study participants. Site will be asked to perform and submit a dummy scan (so called "dummy or dry run") to the central MRI reading center to assess the image quality and to evaluate the compatibility of the electronic data carrier. Once the dummy run has been accepted, all the parameter settings for the study specific MRI sequences must remain unchanged for the duration of the study. Finally, if the site is already pre-approved for performing conventional MRI scan sequences by the imaging vendor the dummy or dry run may be skipped.

Each MRI scan performed for the study needs to be previewed by a local neuro radiologist. During the study, the quality of each scan performed will be assessed by the central MRI reading center. The MRI scan should be sent to the central MRI reading center upon completion of each individual scan. As soon as the scan is received by the central MRI reading center, it will be evaluated for quality, completeness and adherence to the protocol. Confirmation of MRI quality or a description of the quality problems, if detected, will be communicated to the site. If a scan is incomplete or incorrectly performed, the study center will be asked to repeat it as soon as possible. After completion of the quality check, all scans will be analyzed according to the MRI protocol.

Restrictions to MRI schedule

To avoid interferences caused by steroids (in regards to Gd+-enhancing lesions) for the treatment of MS relapse, the following restrictions apply for this study:

- In case of relapse, if an MRI has been scheduled within 30 days of the initiation of steroid treatment, MRI (with Gd+ enhancement) should be performed **before** steroid treatment is initiated.
- No MRI should be performed while a participant is on steroid therapy and within the following 30 days upon termination of steroid treatment.

Because of these restrictions, MRI scheduling can be adjusted accordingly. In case a visit is performed outside the visit window, any subsequent visits should be performed according to the original visit schedule.

Scanning

All sequences/scans will be performed according to the study MRI manual.

MRI scan sequences, such as but not limited to, will include conventional MRI measures of T1 hypointense images (with and without contrast medium, i.e., gadolinium-DTPA), and [redacted]

The gadolinium contrast medium may occasionally cause nausea and vomiting. Allergic reactions may also occur very rarely and, in extremely rare instances, can be potentially serious and require immediate anti anaphylactic treatment. Any AE experience due to the contrast medium should be recorded on the AE eCRF.

[redacted]

[redacted]

[redacted]

[redacted] [redacted]

[redacted]

[redacted] [redacted]

[redacted]

[redacted]

8.3.5 Treatment Satisfaction Questionnaire for Medication TSQM- 9

The Treatment Satisfaction Questionnaire for Medication (TSQM-9) will be used to evaluate the participants' satisfaction with Ofatumumab. The TSQM-9 is a psychometrically sound and valid participant reported outcome to measure participants' satisfaction with medication and a good predictor of adherence across different types of medication and participant population. The TSQM-9 is a 9 item questionnaire having a global satisfaction score ranging from 0-100. The questionnaire consists of 3 domains: satisfaction, convenience and effectiveness (3 items each). The domain scores range from 0 to 100 with higher scores representing higher satisfaction on that domain. (Bharmal et al. 2009)

[redacted]

[redacted]

8.3.8 Appropriateness of efficacy assessments

The [redacted] MRI assessments to be performed in this study are standard and widely accepted efficacy assessments used in clinical MS studies to monitor disease activity and to evaluate treatment effects. They also serve to characterize the participant population in terms of their MS disease status.

[redacted]

The patient reported outcomes will assess important outcomes that impact people living with MS, but are otherwise not captured with efficacy assessments. The TSQM-9, [redacted] in participants with RMS. These assessments have been used in RMS studies.

[redacted]

8.4 Safety

Safety assessments are specified below with the assessment schedule (Table 8-1 and Table 8-2) detailing when each assessment is to be performed.

Safety assessments will include:

- Physical examination (including skin)
- ECG
- Vital signs
- Height and Weight
- Laboratory evaluations
- Columbia Suicide Severity Rating Scale (C-SSRS) (Section 10.2.3)
- Pregnancy testing (women of childbearing potential only)
- Adverse events

Additional safety assessments may be conducted should these be requested by the local regulatory authority. Any new or worsening clinically relevant findings from such additional assessments meeting definition of an adverse event (AE) or serious AE should be recorded as AE/SAE. For details on AE collection and reporting, refer to Section 10.1.1.

8.4.1 Physical Examination

A complete physical examination will be performed at the visits indicated in

Table 8-1 and Table 8-2 and will include an assessment of skin, head and neck, lymph nodes, heart, lungs, abdomen, back, neurological function and comments on general appearance. A complete neurological examination will be part of the initial physical examination at Screening.

Information for all physical examinations (including skin examination) must be included in the source documentation at the study site. Clinically relevant findings that are present prior to signing informed consent must be recorded on the appropriate CRF that captures medical

[redacted]

history. Significant findings made after signing informed consent, which meet the definition of an Adverse Event must be recorded on the adverse events eCRF.

8.4.2 Electrocardiogram (ECG)

All ECGs will be locally collected and evaluated for the eligibility assessment at the Screening visit. Another ECG will be performed at the baseline and EOS visit. Additional unplanned ECGs may be performed at the Investigator's discretion if clinically indicated. Interpretation of the tracing must be made by a qualified physician and documented on the appropriate CRF. Each ECG tracing should be labeled with the study number, participant initials (where regulations permit), participant number, date, and kept in the source documents at the study site. Clinically significant abnormalities present at screening should be reported on the appropriate CRF. Clinically significant findings must be discussed with Novartis prior to enrolling the participant in the study. New or worsened clinically significant findings occurring after informed consent must be recorded as adverse events.

Single 12 lead ECGs are collected. The ECG should be recorded (after 10 minutes rest in the supine position to ensure a stable reading) according to the local site practice. Clinically significant ECG findings at Screening must be discussed with the Novartis Medical Advisor before administration of study drug.

The preferred sequence of cardiovascular data collection during study visits are ECG collection first, followed by vital signs and blood sampling. The Fridericia's QT correction formula (QTcF) should be used for clinical decisions.

The original ECGs on non-heat-sensitive paper (or a certified copy on non-heat-sensitive paper), appropriately signed must be archived at the study site.

Findings and clinically significant abnormalities must be recorded on the relevant section of the medical history form and AE eCRF page as appropriate.

Table 8-4 Local ECG collection plan

Visit	Day	Time	Number of ECG Replicates
Screening	0	Within the 28 day window	12 Lead Single
Baseline/V1	1	Pre-dose	12 Lead Single
V15/EOT/EOS	Month 12	Pre-dose	12 Lead Single
Unscheduled	Unplanned	Anytime	12 Lead Single

8.4.3 Vital Signs

Vital signs will include siting pulse rate (measured as radial pulse for 60 seconds), sitting systolic and diastolic blood pressure and body temperature (oral, or per local practice and should be recorded in the relevant CRF page in Celsius) which will be assessed at the visits indicated in Table 8-1 and Table 8-2.

[redacted]

After the participant has been sitting for five minutes, with back supported and both feet placed on the floor, systolic and diastolic blood pressure will be measured three times using an automated validated device, e.g. OMRON, with an appropriately sized cuff. At the baseline visit only, systolic and diastolic blood pressure should be measured 3 times. The repeat sitting measurements will be made at 1 - 2 minute intervals and the mean of the three measurements will be used. In case the cuff sizes available are not large enough for the participant's arm circumference, a sphygmomanometer with an appropriately sized cuff may be used.

Vital signs should be obtained 30-60 (+/- 10min) minutes before the subcutaneous injection (study drug). If premedication is required, the vital signs should be taken prior to premedication administration.

8.4.4 Height and Weight

Height in centimeters (cm) and body weight (to the nearest 0.1 kilogram (kg) in indoor clothing, but without shoes) will be measured.

8.4.5 Laboratory evaluations

If available, historical IgM and IgG values that have been collected prior to the screening visit, including one pre-anti-CD20 infusion value, should be recorded including the collection date. This should be recorded for all new, existing, and completed participants, and may be entered at any time-point during the study.

A central laboratory will be used for analysis of all specimens collected. Details on the collections, shipment of samples and reporting of results by the central laboratory are provided to Investigators in the laboratory manual.

Abnormal laboratory parameters, inconsistent with clinical presentation of MS, or which cause suspicion of an underlying medical condition should be repeated for confirmation. All abnormal lab results must be evaluated for criteria defining an adverse event and reported as such if the criteria are met. For those lab adverse events, repeated evaluations are mandatory until normalization of the result(s) or until the result is no longer considered clinically significant.

Table 8-5 Laboratory Assessments

Test Category	Test Name
Hematology	Blood samples will be collected according to the schedule in Table 8-1 and Table 8-2. Hematocrit, Hemoglobin, Platelets, Red blood cell (RBC) count, Total WBC count and WBC differential counts (Basophils, Eosinophils, Lymphocytes, Monocytes, Neutrophils).
Chemistry	Blood samples will be collected according to the schedule in Table 8-1 and Table 8-2.

	Electrolytes (Sodium, potassium, chlorine, bicarbonate, calcium, magnesium, phosphorus), random glucose, total protein, urea nitrogen, albumin, alkaline phosphatase, ALT, AST, y-GT, total bilirubin, conjugated bilirubin, creatinine, amylase, total cholesterol, triglycerides, high density lipoprotein and low density lipoprotein, C-Reactive protein.
B-cell/T-cell sampling	$CD19^+$ B-cell counts and $CD3^+CD20^+$ T-cell counts. Samples will be collected according to the schedule in Table 8-1 and Table 8-2.
Total IgG and IgM levels	Samples will be collected according to the schedule in Table 8-1 and Table 8-2.
Urinalysis	Urine will be collected at the scheduled visits indicated in Table 8-1 and Table 8-2. The dipstick parameters assessed will include pH, blood, glucose, specific gravity, WBC, Nitrite, Bilirubin, Ketones and protein. In case of an abnormal dipstick test, a urine sample will be sent to the central laboratory for testing including additional parameters such as microscopy and white blood cell and red blood cell sediments.
Additional tests	Testing of lab samples will be conducted at screening to determine the participant's eligibility for inclusion in the study with respect to hepatitis viruses. Testing for syphilis and tuberculosis at Screening is also needed.<br>A positive result for any of the following serological markers for hepatitis A, B, C and E as below is an exclusion criterion:<br>• anti-HA Immunoglobulin M (IgM)<br>• HBs Ag and/or anti-HBc IgM and/or HB virus deoxyribonucleic acid (DNA)<br>• anti-HBs negative and anti-HBc positive<br>• anti-HC IgG (if positive IgG, HCV RNA PCR will be performed and if negative, participant can be enrolled)

[redacted]

	• anti-HE IgM (if positive IgG and/or IgM, perform HE-RNA PCR and if negative, participant can be enrolled)<br>NOTE: If the Investigator suspects false positive hepatitis serology results, such as an antibody pattern indicating acute hepatitis infection but no corresponding elevated liver enzymes and no signs or symptoms of liver disease, an infectious disease expert may be consulted. If the infectious disease expert finds no evidence of acute or chronic hepatitis infection and considers the serology results false positive and not clinically relevant, the Investigator must document (in source data) that the serology results are considered false positive and may then enroll the participant.
Pregnancy Test	Serum / Urine pregnancy test (refer to Section 8.4.7).

8.4.6 Pregnancy and assessments of fertility

Serum pregnancy tests will be conducted for all women of childbearing potential at each clinic visit including the EOS visit. Urine pregnancy tests will be conducted for all women who are of child bearing potential at all other planned clinic visits as indicated in Table 8-1 and Table 8-2 prior to study drug administration. In addition, the participants will be provided with urinary pregnancy test kits for monthly home pregnancy testing required between the scheduled clinic visits prior to study drug administration. The participants will document the date and result of each home pregnancy test. In case of a positive test result, the participant must contact the Investigator immediately for confirmatory testing at the Investigator's discretion.

In addition, the Investigator will review the contraception status with the participant at each visit to ascertain that the participant continues to comply with protocol requirements for highly effective contraception as applicable.

All pre-menopausal women who are not surgically sterile will have pregnancy testing. Additional pregnancy testing might be performed if required by local requirements.

8.4.7 Appropriateness of safety measurements

The safety assessments included in this study (Table 8-1 and Table 8-2) are standard for the MS indication and study participant population and appropriate based on the current safety profile of ofatumumab as seen in both Phase 2 and Phase 3 studies, see Investigator's Brochure (IB) for more information.

The use of C-SSRS to detect suicidal ideation behavior is currently mandated in studies of CNS active drugs, for further details, refer to Section 10.2.3.

[redacted]

9 Discontinuation and completion

9.1 Discontinuation from study treatment and study

9.1.1 Discontinuation of study treatment

Discontinuation of study drug for a participant occurs when study drug is permanently stopped for any reason (prior to the planned completion of study drug administration, if any), and can be initiated by either the participant or the Investigator.

The Investigator must discontinue study treatment for a given participant if he/she believes that continuation would negatively affect the participant's well-being.

Discontinuation from study treatment is required under the following circumstances:

- Participant/guardian decision (Section 9.2)
- Pregnancy (Section 10.1.6 and Section 10.1.7)
- Use of prohibited treatment (Section 6.2.2)
- Diagnosis of PML
- Participants with active serious infections or reactivation (e.g. tuberculosis, hepatitis B or C)
- Skin and/or mucosal reactions which raise the suspicion of severe generalized major skin reactions (Stevens-Johnson syndrome, or toxic epidermal necrolysis-Lyell's syndrome)
- Hypersensitivity to the study medication
- Any situation in which continued study participation might result in a safety risk to the participant
- Protocol violation that results in a significant risk to the participant's safety
- Emergence of certain adverse events, such as malignancy (except successfully treated basal cell carcinoma, *in situ* squamous cell carcinoma and *in situ* carcinoma of cervix of uterus), liver failure or serious chronic infection (such as human immunodeficiency virus (HIV))
- Laboratory abnormalities (e.g. liver functions tests (LFTs) and abnormal test procedure defined in Appendix 1 (Section 16.1)
- Severe hypoproteinemia
- Interstitial lung disease or new onset or worsening of pulmonary symptoms, such as persistent cough and dyspnea, with or without associated fever, suspicious of interstitial lung disease
- Non-compliance with study drug or study procedures

Study treatment discontinuation should be considered under the following circumstances:

- For participants who meet the criterion for 6-month confirmed disability worsening on EDSS (see Section 10.1.5) during the study, the Investigator must reassess the benefits and risks of continuing study treatment. The Investigator will also discuss the further treatment with the participant, including any other MS treatment options that may be available to the participant. The outcome of the discussion with the participant must be documented in the participant's source information

[redacted]

- In case the Investigator deems the benefit-risk of continuing study treatment to be unfavorable or if the participant does not wish to continue study treatment and the participant does not initiate a commercial MS disease modifying therapy, the participant will be discontinued from the study and will enter the Safety Follow-up period (Table 8-2).

If discontinuation from study treatment occurs, the Investigator should make a reasonable effort to understand the primary reason for the participant's discontinuation from study treatment and record this information.

Participants who discontinue from study treatment agree to return for the end of treatment and follow-up visits indicated in the Assessment Schedule (see Table 8-1). Participants that discontinue treatment and do not initiate a commercial MS disease modifying therapy should complete the EOS visit and move to the Safety Follow-up as per Table 8-2. If they fail to return for these assessments for unknown reasons, every effort (e.g. telephone, e-mail, letter) should be made to contact the participant/pre-designated contact as specified in Section 9.1.3. This contact should preferably be done according to the study visit schedule.

If the participant cannot or is unwilling to attend any visit(s), the site staff should maintain regular contact with the participant, or with a person pre-designated by the participant. This contact should preferably be done according to the study visit schedule for up to 6 months post last study drug administration.

After discontinuation from study treatment at a minimum, in abbreviated visits, the following data should be collected at clinic visits or via telephone/email contact:

- New / concomitant treatments
- Adverse Events / Serious Adverse Events

The Investigator must also contact the IRT to register the participant's discontinuation from study drug.

9.1.2 Discontinuation from study

Discontinuation from study is when the participant permanently stops receiving the study treatment, and further protocol-required assessments or follow-up, for any reason. If the participant agrees, a final evaluation at the time of the participant's study discontinuation should be made as detailed in the assessment table (refer to Section 8).

9.1.3 Lost to follow-up

For participants whose status is unclear because they fail to appear for study visits without stating an intention to discontinue from study treatment or discontinue from study or withdraw consent/oppose to the use of their data/biological samples, the Investigator must show "due diligence" by documenting in the source documents steps taken to contact the participant, e.g. dates of telephone calls, registered letters, etc. A participant should not be considered as lost to follow-up until due diligence has been completed or until the end of the study.

[redacted]

9.2 Withdrawal of informed consent/Opposition to use

Participants may voluntarily withdraw consent to participate in the study for any reason at any time. Withdrawal of consent occurs only when a participant:

- Does not want to participate in the study anymore, and
- Does not allow further visits or assessments, and
- Does not want any further study related contacts

This request should be in writing (depending on local regulations) and recorded in the source documentation.

In this situation, the Investigator should make a reasonable effort (e.g. telephone, e-mail, letter) to understand the primary reason for the Participant's decision to withdraw his/her consent/opposition to use data/biological samples and record this information.

Where consent to the use of personal and coded data is not required, participant therefore cannot withdraw consent. However, they still retain the right to object to the further collection or use of personal data.

Study treatment must be discontinued and no further assessments conducted, and the data that would have been collected at subsequent visits will be considered missing.

Further attempts to contact the participant are not allowed unless safety findings require communicating or follow-up.

All efforts should be made to complete the assessments prior to study withdrawal. If the participant agrees, a final evaluation at the time of the participant's withdrawal of consent/opposition to use data/biological samples should be made as detailed in the assessment table (section 8)

Novartis will continue to retain and use all research results (data) that have already been collected for the study evaluation including processing of biological samples that has already started at the time of consent withdrawal/opposition. No new Personal Data (including biological samples) will be collected following withdrawal of consent/opposition according to applicable law.

For US: All biological samples not yet analyzed at the time of withdrawal may still be used for further testing/analysis in accordance with the terms of this protocol and of the informed consent form.

9.3 Study completion and post-study treatment

Study completion is defined as when the last participant finishes their Study Completion visit and any repeat assessments associated with this visit have been documented and followed-up appropriately by the Investigator or, in the event of an early study termination decision, the date of that decision.

A participant is considered to have completed the study when they fulfill the following criteria:

- Participant has completed the study in its entirety according to the approved duration of the study within that country

[redacted]

- The participant has **not** stopped the study due to one or more of the following reasons:
  - Discontinuation of study treatment (Section 9.1.1)
  - Withdrawal of informed consent (Section 9.2)
  - Lost to follow-up (Section 9.1.3)

End of Study (EOS) visit is mandatory for all participants. For participants that complete the study, the next (and last) scheduled visit is the EOS visit and should align with the overall visit schedule. Participants will then enter the Safety Follow-up according to the assessment schedule in Table 8-2 if they do not initiate a commercial MS disease modifying therapy. Participants who prematurely discontinue study treatment will have their EOS visit as soon as possible and continue into the Safety Follow-up for 6 months according to the assessment schedule in Table 8-2 if they do not initiate a commercial MS disease modifying therapy.

The Investigator must provide follow-up medical care for all Participants who are prematurely withdrawn from the study, or must refer them for appropriate ongoing care.

9.4 Early study termination by the sponsor

The study can be terminated by Novartis at any time for any reason. This may include reasons related to the benefit/ risk assessment of participating in the study, practical reasons (including slow enrollment), or for regulatory or medical reasons. In taking the decision to terminate, Novartis will always consider the participant welfare and safety. Should early termination be necessary, participants must be seen as soon as possible and treated as a prematurely withdrawn participant.

The Investigator may be informed of additional procedures to be followed in order to ensure that adequate consideration is given to the protection of the participant's interests. The Investigator or sponsor depending on the local regulation will be responsible for informing IRBs/IECs of the early termination of the trial

10 Safety monitoring and reporting

10.1 Definition of adverse events and reporting requirements

10.1.1 Adverse events

An adverse event (AE) is any untoward medical occurrence (e.g. any unfavorable and unintended sign [including abnormal laboratory findings], symptom or disease) in a participant or clinical investigation participant after providing written informed consent for participation in the study. Therefore, an AE may or may not be temporally or causally associated with the use of a medicinal (investigational) product.

In addition, all reports of intentional misuse and abuse of the product are also considered an adverse event irrespective of if a clinical event has occurred.

Novartis qualified medical personnel will be readily available to advise on trial related medical questions or problems.

The occurrence of adverse events must be sought by non-directive questioning of the participant at each visit during the study. Adverse events also may be detected when they are volunteered

[redacted]

by the participant during or between visits or through physical examination findings, laboratory test findings, or other assessments.

Abnormal laboratory values or test results constitute adverse event only if they fulfill at least one of the following criteria:

- they induce clinical signs or symptoms
- they are considered clinically significant
- they require therapy

Clinically significant abnormal laboratory values or test results must be identified through a review of values outside of normal ranges/clinically notable ranges, significant changes from baseline or the previous visit, or values, which are considered non-typical in participants with the underlying disease. Investigators have the responsibility for managing the safety of individual participant and identifying adverse events. Clinically notable laboratory findings are defined according to the Common Terminology Criteria for Adverse Events (CTCAE) (the most current version will be used and can be found on the following web-site: http://ctep.cancer.gov).

Adverse events must be recorded under the signs, symptoms, or diagnosis associated with them, accompanied by the following information (as far as possible) (if the event is serious refer to Section 10.1.2):

- The Common Toxicity Criteria (CTC) AE grade (1-4)

If CTCAE grading does not exist for an adverse event, use:

1 = mild: usually transient in nature and generally not interfering with normal activities

2 = moderate: sufficiently discomforting to interfere with normal activities

3 = severe: prevents normal activities

4 = life-threatening

- Its relationship to the study treatment.
- Its duration (start and end dates) or if the event is ongoing, an outcome of not recovered/not resolved must be reported
- Whether it constitutes a SAE (see Section 10.1.2 for definition of SAE) and which seriousness criteria have been met
- Action taken regarding with study treatment

All adverse events must be treated appropriately. Treatment may include one or more of the following:

- No action taken (e.g. further observation only)
- Drug interrupted/withdrawn
- Concomitant medication or non-drug therapy given
- Participant hospitalization/participant's hospitalization prolonged (see Section 10.1.2 for definition of SAE)
- Its outcome (not recovered/not resolved; recovered/resolved; recovered/resolved with sequelae; fatal; or unknown).

[redacted]

Conditions that were already present at the time of informed consent should be recorded in medical history of the participant.

Adverse events (including lab abnormalities that constitute AEs) should be described using a diagnosis whenever possible, rather than individual underlying signs and symptoms.

Adverse event monitoring should be continued following the last dose of study treatment until the participant starts a commercially available MS disease modifying therapy or until their B cells are repleted. Repletion is defined as a concentration > the participant's baseline value prior to starting the IV anti-CD20 mAb or > the lower limit of normal, whichever is observed first.

Once an adverse event is detected, it must be followed until its resolution or until it is judged to be permanent (e.g. continuing at the end of the study), and assessment must be made at each visit (or more frequently, if necessary) of any changes in severity, the suspected relationship to the interventions required to treat it, and the outcome.

Information about adverse drug reactions for the investigational drug (ofatumumab) can be found in the Investigator's Brochure (IB). This information will be included in the participant informed consent and should be discussed with the participant during the study as needed. Any new information regarding the safety profile of ofatumumab that is identified between IB updates will be communicated as appropriate, for example, via an Investigator Notification or an Aggregate Safety Finding. New information might require an update to the informed consent and has then to be discussed with the participant.

The Investigator must also instruct each participant to report any new adverse event (beyond the protocol observation period) that the participant, or the participant's personal physician, believes might reasonably be related to study treatment. This information must be recorded in the Investigator's source documents; however, if the AE meets the criteria of an SAE, it must be reported to Novartis.

10.1.2 Serious adverse events

An SAE is defined as any adverse event [appearance of (or worsening of any pre-existing)] undesirable sign(s), symptom(s), or medical conditions(s) which meets any one of the following criteria:

- fatal
- life-threatening

Life-threatening in the context of a SAE refers to a reaction in which the participant was at risk of death at the time of the reaction; it does not refer to a reaction that hypothetically might have caused death if it were more severe (please refer to the ICH-E2D Guidelines).

- results in persistent or significant disability/incapacity
- constitutes a congenital anomaly/birth defect
- requires inpatient hospitalization or prolongation of existing hospitalization, unless hospitalization is for:
    - routine treatment or monitoring of the studied indication, not associated with any deterioration in condition (e.g. hospitalization for MS relapse treatment)

[redacted]

  - elective or pre-planned treatment for a pre-existing condition that is unrelated to the indication under study and has not worsened since signing the informed consent
  - social reasons and respite care in the absence of any deterioration in the participant's general condition
  - treatment on an emergency outpatient basis for an event not fulfilling any of the definitions of a SAE given above and not resulting in hospital admission
- is medically significant, e.g. defined as an event that jeopardizes the participant or may require medical or surgical intervention to prevent one of the outcomes listed above

Medical and scientific judgment should be exercised in deciding whether other situations should be considered serious reactions, such as important medical events that might not be immediately life threatening or result in death or hospitalization but might jeopardize the participant or might require intervention to prevent one of the other outcomes listed above. Such events should be considered as "medically significant." Examples of such events are intensive treatment in an emergency room or at home for allergic bronchospasm, blood dyscrasias, or convulsions that do not result in hospitalization or development of dependency or abuse (please refer to the ICH-E2D Guidelines).

All malignant neoplasms will be assessed as serious under "medically significant" if other seriousness criteria are not met.

Any suspected transmission via a medicinal product of an infectious agent is also considered a serious adverse reaction.

All reports of intentional misuse and abuse of the product are also considered serious adverse event irrespective if a clinical event has occurred, and are to be reported as appropriate according to Section 10.1.8.

10.1.3 SAE reporting

To ensure participant safety, every SAE, regardless of causality, occurring after the participant has provided informed consent and until B cells are repleted after the final study drug administration for study completers or until they initiate a commercially available MS disease modifying therapy or until end of Safety Follow-up for premature withdrawals must be reported to Novartis safety immediately, without undue delay, under no circumstances later than within 24 hours of learning of its occurrence. Detailed instructions regarding the submission process and requirements are to be found in the Investigator folder provided to each site.

All follow-up information for the SAE including information on complications, progression of the initial SAE and recurrent episodes must be reported as follow-up to the original episode within 24 hours of the Investigator receiving the follow-up information. An SAE occurring at a different time interval or otherwise considered completely unrelated to a previously reported one must be reported separately as a new event.

Information about all SAEs is collected and recorded on the Serious Adverse Event Report Form; all applicable sections of the form must be completed in order to provide a clinically thorough report. The Investigator must assess the relationship of each SAE to each specific component of study treatment (if study treatment consists of several components) complete the SAE Report Form in English, and submit the completed form within 24 hours to Novartis.

[redacted]

Detailed instructions regarding the submission process and requirements for signature are to be found in the Investigator folder provided to each site.

Follow-up information is submitted as instructed in the Investigator folder. Each re-occurrence, complication, or progression of the original event must be reported as a follow-up to that event regardless of when it occurs. The follow-up information should describe whether the event has resolved or continues, if and how it was treated and whether the participant continued or withdrew from study participation.

If the SAE is not previously documented in the Investigator's Brochure or Package Insert (new occurrence) and is thought to be related to the study treatment, a CMO & PS Department associate may urgently require further information from the Investigator for health authority reporting. Novartis may need to issue an Investigator Notification (IN) to inform all Investigators involved in any study with the same study treatment that this SAE has been reported.

Suspected Unexpected Serious Adverse Reactions (SUSARs) will be collected and reported to the competent authorities and relevant ethics committees in accordance with EU Guidance 2011/C 172/01 or as per national regulatory requirements in participating countries.

Any SAEs experienced after the B cells are repleted following final study drug administration or after the end of Safety Follow-up should only be reported to Novartis Safety if the Investigator suspects a causal relationship to study treatment unless otherwise specified by local law/regulations.

10.1.4 Reporting MS relapse as a SAE

MS relapses are one of the efficacy endpoints in this study; hence, they are exempt from SAE reporting although they may meet the SAE definition on the basis that they are considered medically significant and are frequently associated with hospitalization. These events will therefore be reported on the MS relapse eCRF instead of the SAE form. However, if, in the judgment of the Investigator, a MS relapse is unusually severe or medically unexpected and warrants specific notification, then an SAE form must be completed and submitted according to SAE reporting procedures outlined above.

10.1.5 Reporting of Disability Worsening as a SAE

Disability worsening is not one of the efficacy endpoints in this study and therefore should be reported as an AE. If, in the judgment of the Investigator the disability worsening is unusually severe or medically unexpected and warrants specific notification, then a SAE form must be completed and submitted according to SAE reporting procedures outlined above.

A 6-month confirmed disability worsening is defined as an increase from baseline in EDSS sustained for at least 6 months. This means that after a scheduled or unscheduled visit at which the patient fulfills the disability worsening criterion, all EDSS assessments (scheduled or unscheduled) need to also fulfill the worsening criteria until the worsening ("the event") can be confirmed at the first scheduled visit that occurs 6 months after the onset of the worsening, or later.

[redacted]

Table 10-1 Criterion for disability worsening based on change in EDSS score

Total EDSS at Baseline	“Disability worsening” criterion
0<br>1 to 5<br>≥5.5	≥ +1.5<br>≥ +1<br>≥ +0.5

EDSS=Expanded Disability Status Scale
A 6-month confirmed disability worsening (6-mCDW) can have an onset at any scheduled unscheduled visit if the disability worsening criterion is met.
A disability worsening event can only be confirmed at a scheduled visit if, over a period of 6 months (≥ 166 days=6*30-14) time interval, all assessments meet the worsening criterion.
If a patients dies due to MS (EDSS=10 at any time), it will be considered a confirmed disability worsening regardless of the baseline EDSS or the change in EDSS.
* Baseline EDSS is defined as the last EDSS assessment prior to the first dose of study medication (protocol inclusion criterion is EDSS 0-5.5)

10.1.6 Pregnancy reporting

To ensure participant safety, each pregnancy occurring after signing the informed consent must be reported to Novartis within 24 hours of learning of its occurrence. The pregnancy should be followed up until after the Expected Delivery Date (EDD) to determine outcome, including spontaneous or voluntary termination, details of the birth, and the presence or absence of any birth defects, congenital abnormalities, or maternal and/or newborn complications. Additional follow up will be completed at EDD +1 month, EDD +2 months (in case no answer is received after request at EDD +1 month) and at EDD +3 months. Information on the status of the baby after delivery and information on any development issue or abnormality that would not be seen at birth must be collected.

10.1.7 Pregnancies

If a female trial participant becomes pregnant, the study treatment should be stopped, and the pregnancy consent form should be presented to the trial participant. The participant must be given adequate time to read, review and sign the pregnancy consent form. This consent form is necessary to allow the investigator to collect and report information regarding the pregnancy. To ensure participant safety, each pregnancy occurring after signing the informed consent must be reported to Novartis, within 24 hours of learning of its occurrence. The pregnancy should be followed up to determine outcome, including spontaneous or voluntary termination details of the birth, and the presence or absence of any birth defects, congenital abnormalities, or maternal and/or newborn complications.

Pregnancy should be recorded on the Pharmacovigilance Pregnancy Form and reported by the Investigator to the Novartis Chief Medical Office and Patient Safety (CMO&PS). Pregnancy follow-up should be recorded on the same form and should include an assessment of the possible relationship to the study treatment any pregnancy outcome. Any SAE experienced during pregnancy must be reported on a SAE form.

[redacted]

10.1.8 Reporting of study treatment errors including misuse/abuse

Medication errors are unintentional errors in the prescribing, dispensing, administration or monitoring of a medicine while under the control of a healthcare professional, patient or consumer (EMA) definition).

Misuse refers to situations where the medicinal product is intentionally and inappropriately used not in accordance with the protocol.

Abuse corresponds to the persistent or sporadic, intentional excessive use of a medicinal product, which is accompanied by harmful physical or psychological effects.

Study treatment errors and uses outside of what is foreseen in the protocol will be recorded on the appropriate CRF irrespective of whether or not associated with an AE/SAE and reported to Safety only if associated with an SAE. Misuse or abuse will be collected and reported in the safety database irrespective of it being associated with an AE/SAE within 24 hours of Investigator's awareness.

Table 10-2 Guidance for capturing the study treatment errors including misuse/abuse

Treatment error type	Document in Dosing CRF (Yes/No)	Document in AE eCRF	Complete SAE form
Unintentional study treatment error	Yes	Only if associated with an AE	Only if associated with an SAE
Misuse/Abuse	Yes	Yes	Yes, even if not associated with a SAE

For more information on AE and SAE definition and reporting requirements, please see the respective sections.

10.2 Additional Safety Monitoring

10.2.1 Liver safety monitoring

To ensure participant safety and enhance reliability in determining the hepatotoxic potential of an investigational drug, a standardized process for identification, monitoring and evaluation of liver events has to be followed.

The following two categories of abnormalities / adverse events have to be considered during the course of the study (irrespective of whether classified/reported as AE/SAE):

- Liver laboratory triggers, which will require repeated assessments of the abnormal laboratory parameter
- Liver events, which will require close observation, follow-up monitoring and contributing factors are recorded on the appropriate CRFs

Please refer to Table 16-1 in Appendix 1 for complete definitions of liver laboratory triggers and liver events.

[redacted]

Every liver event defined in Table 16-1 should be followed up by the Investigator or designated personnel at the trial site, as summarized below. Additional details on actions required in case of liver events are outlined in Table 16-2.

For all liver event triggers, liver chemistry tests (i.e. ALT, AST, TBL, PT/INR, ALP and y-GT) must be repeated within the next week to confirm elevation.

- These liver chemistry repeats will be performed using the central laboratory. If results will not be available from the central laboratory, then the repeats can also be performed at a local laboratory to monitor the safety of the participant.
- If a liver event is subsequently reported, any local liver chemistry tests previously conducted that are associated with this event should have results recorded on the appropriate CRF. Repeat laboratory examinations must then be performed at central laboratory as soon as possible.
- If the initial elevation is confirmed, close observation of the participant will be initiated, including consideration of treatment interruption if deemed appropriate.
- Discontinuation of the investigational drug (refer to Section 9.1.1), if appropriate
- Hospitalization of the participant if appropriate
- Causality assessment of the liver event
- Thorough follow-up of the liver event should include
    - These investigations can include based on Investigator's discretion: serology tests, imaging and pathology assessments, hepatologist's consultancy; obtaining more detailed history of symptoms and prior or concurrent diseases, history of concomitant drug use, exclusion of underlying liver disease

All follow-up information and procedures performed must be recorded as appropriate in the CRF.

10.2.2 Renal safety monitoring

The following two categories of abnormal renal laboratory values have to be considered during the course of the study:

- Serum creatinine (sCR) increase ≥ 25% compared to baseline during normal hydration status, confirmed after 24 hours
- new onset (≥ 1+) proteinuria; confirmed by doubling in the urinary albumin/creatinine ratio (ACR) or urinary protein-creatinine ratio (PCR) (if applicable)
- new onset (≥ 1+), hematuria or glycosuria

Abnormal renal event findings must be confirmed after ≥24 hours but ≤ 5 days after first assessment.

Every renal laboratory trigger or renal event as defined in Table 16-3 should be followed up by the Investigator or designated personnel at the trial site as summarized in Table 16-4.

[redacted]

10.2.3 Prospective suicidality assessment

The C-SSRS is a paper questionnaire that prospectively assesses suicidal ideation and suicidal behavior. The C-SSRS can be administered at each visit (as per Table 8-1), including unplanned visits.

At the screening visit, the "baseline/screening" version of the C-SSRS will be administered. This version assesses suicidal ideation and suicidal behavior during the participant's lifetime and during a predefined period. At subsequent visits, the "since last visit" version will be administered.

If, at any time after screening, the score is "yes" on item 4 or item 5 of the suicidal ideation section of the C-SSRS or "yes" on any item of the suicidal behavior section, the participant must be referred to a mental health care professional for further assessment and/or treatment. The decision on whether the study drug should be discontinued is to be taken by the Investigator in consultation with the mental health professional to whom the participant is referred.

In addition, all life-threatening events must be reported as SAEs. For example, if a participant answers "yes" to one of the questions in the suicidal behavior section, an SAE must be reported if the event was life-threatening. All events of "Non-Suicidal Self-Injurious Behavior" (question also included in the suicidal behavior section) should be reported as AEs and assigned the appropriate severity grade.

10.2.4 Data Monitoring Committee

No data monitoring committee will be implemented given that the study is open label. Data will be reviewed on an ongoing basis by the medical monitor and the clinical study team including safety data scientist and statistician.

10.2.5 Steering Committee

The Steering Committee (SC) will be established comprising Investigators participating in the trial, i.e. not being members of the Novartis/sponsor representative from the Clinical Trial Team.

The SC will ensure transparent management of the study according to the protocol through recommending and approving modifications as circumstances require. The SC will review protocol amendments as appropriate. Together with the clinical trial team, the SC will also develop recommendations for publications of study results including authorship rules. The details of the role of the steering committee will be defined in the "Steering Committee Charter". A study steering committee will be appointed prior to first patient first visit (FPFV).

11 Data Collection and Database management

11.1 Data collection

Designated Investigator staff will enter the data required by the protocol into the Electronic Case Report Forms (eCRF). The eCRFs have been built using fully validated secure web-enabled software that conforms to 21 CFR Part 11 requirements, Investigator site staff will not be given access to the EDC system until they have been trained. Automatic validation programs

[redacted]

check for data discrepancies in the eCRFs, allow modification and/or verification of the entered data by the Investigator staff.

The Investigator/designee is responsible for assuring that the data (recorded on CRFs) (entered into eCRF) is complete, accurate, and that entry and updates are performed in a timely manner. The Investigator must certify that the data entered are complete and accurate.

All other data captured for this study will have either the eCRF as source or an external originating source (either written or electronic) with the CRF not being considered as source. In cases where electronic source data capture devices experience faults, for example, digital device used for ePROs, paper back-up source will be permitted.

After final database lock, the Investigator will receive copies of the participant data for archiving at the investigational site.

All data should be recorded, handled, and stored in a way that allows its accurate reporting, interpretation, and verification.

11.2 Database management and quality control

Novartis personnel (or designated CRO) will review the data entered by investigational staff for completeness and accuracy. Electronic data queries stating the nature of the problem and requesting clarification will be created for discrepancies and missing values and sent to the investigational site via the EDC system. Designated Investigator site staff are required to respond promptly to queries and to make any necessary changes to the data.

Concomitant treatments and prior medications entered into the database will be coded using the World Health Organization (WHO) Drug Reference List, which employs the Anatomical Therapeutic Chemical classification system. Medical history/current medical conditions and adverse events will be coded using the Medical Dictionary for Regulatory Activities (MedDRA) terminology.

Laboratory samples will be processed centrally and the results will be sent electronically to Sponsor. MRI scans will be analyzed centrally the derived results will also be sent electronically to Sponsor.

Enrollment codes and data about all study treatment(s) dispensed to the participant will be tracked using an Interactive Response Technology (IRT). The system will be supplied by a vendor, who will also manage the database.

For electronic COAs, devices will be supplied by a vendor, data will be processed centrally, and both raw and processed data will be sent electronically to Novartis (or a designated CRO).

Once all the necessary actions have been completed, including determining the occurrence of relevant protocol deviations, and the database has been declared to be complete and accurate, it will be locked and made available for data analysis. Any changes to the database after that time can only be made after written agreement by Novartis development management.

11.3 Site monitoring

Before study initiation, at a site initiation visit or at an Investigator's meeting, a Novartis representative will review the protocol and data capture requirements (i.e. eSource

[redacted]

DDE or eCRFs) with the Investigators and their staff. During the study, Novartis employs several methods of ensuring protocol and GCP compliance and the quality/integrity of the sites' data. The field monitor will visit the site to check the completeness of participant records, the accuracy of data capture / data entry, the adherence to the protocol and to Good Clinical Practice, the progress of enrollment, and to ensure that study treatment is being stored, dispensed, and accounted for according to specifications. Key study personnel must be available to assist the field monitor during these visits. Continuous remote monitoring of each site's data may be performed by a centralized Novartis CRA organization. Additionally, a central analytics organization may analyze data & identify risks & trends for site operational parameters, and provide reports to Novartis clinical teams to assist with trial oversight.

The Investigator must maintain source documents for each participant in the study, consisting of case and visit notes (hospital or clinic medical records) containing demographic and medical information, laboratory data, electrocardiograms, and the results of any other tests or assessments. All information on CRFs must be traceable to these source documents in the participant's file. The Investigator must also keep the original informed consent form signed by the participant (a signed copy is given to the participant).

The Investigator must give the monitor access to all relevant source documents to confirm their consistency with the data capture and/or data entry. Novartis monitoring standards require full verification for the presence of informed consent, adherence to the inclusion/exclusion criteria, documentation of SAEs, and of data that will be used for all primary variables. Additional checks of the consistency of the source data with the CRFs are performed according to the study-specific monitoring plan. No information in source documents about the identity of the participants will be disclosed.

11.4 Potential Remote Study Visits

If a pandemic or a national and/or global emergency were to occur, remote study visits may be available for participants unable to travel to the clinic for their study visit. This would allow the opportunity for participants, if desired, to continue their participation in the study.

12 Data analysis and statistical methods

The analysis will be conducted on all participant data at the time the trial ends. Any data analysis carried out independently by the Investigator should be submitted to Novartis before publication or presentation.

Categorical variables will be presented as counts and percentages. For continuous variable, mean, standard deviation, minimum, 25th percentile, median, 75th percentile, and maximum will be presented.

12.1 Analysis sets

The Full Analysis Set (FAS) comprises all participants who received at least one dose of study drug.

The FAS will be used for the summary of demography and baseline characteristics as well as for all efficacy analyses.

[redacted]

The Safety Set is identical to FAS in this study.

12.2 Participant demographics and other baseline characteristics

Demographics, MS disease history, MRI baseline characteristics and MS medication history will be summarized for the FAS.

Relevant medical histories and current medical conditions at baseline will be summarized by system organ class and preferred term.

12.3 Treatments

The duration of exposure in (days) to study medication will be summarized descriptively.

Concomitant medications and significant non-drug therapies prior to and after the start of the study treatment will be listed and summarized according to the Anatomical Therapeutic Chemical (ATC) classification system and preferred term.

12.4 Analysis of the primary endpoint(s)

The primary aim of this study is to explore maintenance of efficacy evaluated by MRI lesions at twelve months after initiating ofatumumab therapy in RMS participants transitioning to ofatumumab from aCD20 therapy (for reasons other than suboptimal response or the occurrence of certain treatment-emergent adverse events).

12.4.1 Definition of primary endpoint(s)

The primary endpoint will be no change or a reduction from Baseline (assessed at Screening Visit) in the number of gadolinium enhancing lesions observed by MRI at Month 12, a binary outcome (yes, no).

12.4.2 Statistical model, hypothesis, and method of analysis

The number and percentage of participants with no change or a reduction in number of gadolinium enhancing lesions at Month 12 will be presented. The 95% confidence interval for the proportion of participants with no change or a reduction will be calculated by using normal approximation method.

Both non-response imputation for missing data and observed data approaches will be applied.

The above analyses will be conducted using the Full-Analysis-Set (FAS).

12.4.3 Handling of intercurrent events of primary estimand

Missing data will be handled using non-response imputation regardless of intercurrent event.

12.4.4 Handling of missing values/censoring/discontinuations

For participants with missing number of gadolinium enhancing lesions at Month 6 and Month 12, non-response imputation method will be used.

12.4.5 Sensitivity and Supportive analyses

Neither sensitivity nor supportive analysis is planned.

[redacted]

12.5 Analysis of secondary endpoints

12.5.1 Efficacy endpoint(s)

Retention on ofatumumab treatment from baseline to Month 6, and to Month 12 (yes, no) will be summarized using frequency count and percentage for the FAS.

12.5.2 Safety endpoints

For all safety analyses, the safety set will be used. All listings and tables will be presented by treatment. Unless explicitly otherwise stated, only data up to and until B cells are repleted after the final study drug administration for study completers or until the participant initiates a commercial MS disease modifying therapy or until the end of Safety Follow-up for premature withdrawals will be included in the analysis and data beyond this time point for a participant will be excluded from safety analysis. In addition, a separate summary for death including on treatment and post treatment deaths will be provided. In particular, summary tables for adverse events (AEs) will summarize only on-treatment events, which started or worsened during the on-treatment period (treatment-emergent AEs).

The on-treatment period for safety analysis lasts from the date of first administration of study drug until B cells are repleted after the date of the last actual administration of study drug for study completers or until the participant initiates a commercial MS disease modifying therapy or until the end of Safety Follow-up.

Adverse events

All information obtained on adverse events will be displayed by treatment and participant. The number and percentage of participants with AEs of special interest/related to identified and potential risks will be summarized in the following ways:

- By treatment, primary system organ class and preferred term.
- By treatment, primary system organ class, preferred term and maximum severity.
- By treatment, pre-defined topic of interest, primary system organ class and preferred term.

Separate summaries will be provided for study medication related adverse events, serious adverse events, and other significant adverse events leading to discontinuation.

The number and percentage of participants with adverse events of special interest will be summarized by treatment, primary system organ class and preferred term. A participant with multiple AEs within a category is only counted once towards the total of that category.

Vital signs

All vital signs data may be listed by participant, and visit. Abnormalities will be flagged. Descriptive statistics for change from baseline will be provided by visit.

[redacted]

Clinical laboratory evaluations

All abnormal laboratory values will be listed participant and visit, if normal ranges are available. Summary statistics for change from baseline will be provided by visit. Shift tables using the low/normal/high/ (low and high) classification will be used to compare baseline to the worst on-treatment value. In addition, historical change of IgM and IgG will be summarized.

Other safety evaluations

All clinically significant safety findings based on additional safety evaluations (e.g. physical examination including assessments of skin, lymph nodes, lung etc.) must be reported as adverse events on the AE CRF. The statistical analysis of these findings will be done in the analysis of adverse events. Other safety data will be summarized or listed as appropriate.

In addition, change from baseline in lymphocytes, including total CD19+ B cell counts and CD3+CD20+ T cell counts, obtained by fluorescence activated cell sorting (FACS) will be summarized descriptively.

Suicidality evaluations

The C-SSRS data (safety data cutoff applies) will be mapped to Columbia Classification Algorithm for Suicide assessment (C-CASA) as per Food and Drug Administration (FDA) guidance on suicidality. Descriptive statistics will be provided for change from baseline to Months 6 and 12.

Patient reported outcomes

Treatment Satisfaction Questionnaire for Medication (TSQM-9) scores (including domains) at Baseline vs Month 6 and vs Month 12 will be summarized.

12.5.3 Biomarkers

No secondary biomarker analysis is planned.

[redacted]

[redacted]

[redacted]

[redacted]

12.7 Interim analyses

For the purpose of earlier dissemination of results, an interim analysis will be performed after all enrolled participants have either meet the criteria of at least 6 months treatment or discontinued treatment prematurely. For this analysis, only primary variables, demographics, baseline characteristics, adverse events and serious adverse events will be summarized.

Since there will be no hypothesis testing involved, statistical adjustment for the interim analysis will not be made at the stage of final analyses of efficacy and safety.

12.8 Sample size calculation

12.8.1 Primary endpoint(s)

Sample size calculations were based on the proportion of participants with no change or a reduction from Baseline (assessed at Screening Visit) in the number of gadolinium-enhancing (GdE) MRI lesions 12 months after initiating ofatumumab therapy. Three precision estimates were considered for this study:

The sample size of 100 participants is selected based on budget and early availability of interim analysis results. This sample size of 100 participants will provide us with an 8.5% precision

[redacted]

(half-width of 95% confidence interval), a 7.8% precision, or a 7.0% precision corresponding to estimated proportions of 75%, 80%, and 85% of participants achieving the primary endpoint.

13 Ethical considerations and administrative procedures

13.1 Regulatory and ethical compliance

This clinical study was designed and shall be implemented, executed and reported in accordance with the International Conference on Harmonisation (ICH) Harmonized Tripartite Guidelines for Good Clinical Practice, with applicable local regulations (including European Directive 2001/20/EC, US CFR 21), and with the ethical principles laid down in the Declaration of Helsinki.

13.2 Responsibilities of the Investigator and IRB/IEC

Before initiating a trial, the Investigator/institution must obtain approval/favorable opinion from the Institutional Review Board/Independent Ethics Committee (IRB/IEC) for the trial protocol, written informed consent form, consent form updates, participant recruitment procedures (e.g. advertisements) and any other written information to be provided to participants. Prior to study start, the Investigator is required to sign a protocol signature page confirming his/her agreement to conduct the study in accordance with these documents and all of the instructions and procedures found in this protocol and to give access to all relevant data and records to Novartis monitors, auditors, Novartis Quality Assurance representatives, designated agents of Novartis, IRBs/IECs, and regulatory authorities as required. If an inspection of the clinical site is requested by a regulatory authority, the Investigator must inform Novartis immediately that this request has been made.

13.3 Publication of study protocol and results

The protocol will be registered in a publicly accessible database such as clinicaltrials.gov and as required in EudraCT. In addition, after study completion (*defined as last patient last visit*) and finalization of the study report the results of this trial will be submitted for publication and posted in a publicly accessible database of clinical trial results, such as the Novartis clinical trial results website and all required Health Authority websites (e.g. Clinicaltrials.gov, EudraCT etc.) .

For details on the Novartis publication policy including authorship criteria, please refer to the Novartis publication policy training materials that were provided to you at the trial Investigator meetings.

13.4 Quality Control and Quality Assurance

Novartis maintains a robust Quality Management System (QMS) that includes all activities involved in quality assurance and quality control, to ensure compliance with written Standard Operating Procedures as well as applicable global/local GCP regulations and ICH Guidelines.

Audits of Investigator sites, vendors, and Novartis systems are performed by auditors, independent from those involved in conducting, monitoring or performing quality control of the clinical trial. The clinical audit process uses a knowledge/risk based approach.

[redacted]

Audits are conducted to assess GCP compliance with global and local regulatory requirements, protocols and internal SOPs, and are performed according to written Novartis processes

14 Protocol adherence

This protocol defines the study objectives, the study procedures and the data to be collected on study participants. Additional assessments required to ensure safety of participants should be administered as deemed necessary on a case by case basis. Under no circumstances including incidental collection is an Investigator allowed to collect additional data or conduct any additional procedures for any purpose involving any investigational drugs under the protocol, other than the purpose of the study. If despite this interdiction prohibition, data, information, observation would be incidentally collected, the Investigator shall immediately disclose it to Novartis and not use it for any purpose other than the study, except for the appropriate monitoring on study participants.

Investigators ascertain they will apply due diligence to avoid protocol deviations. If an Investigator feels a protocol deviation would improve the conduct of the study this must be considered a protocol amendment, and unless such an amendment is agreed upon by Novartis and approved by the IRB/IEC and Health Authorities, where required, it cannot be implemented.

14.1 Protocol amendments

Any change or addition to the protocol can only be made in a written protocol amendment that must be approved by Novartis, health authorities where required, and the IRB/IEC prior to implementation.

Only amendments that are required for participant safety may be implemented immediately provided the health authorities are subsequently notified by protocol amendment and the reviewing IRB/IEC is notified.

Notwithstanding the need for approval of formal protocol amendments, the Investigator is expected to take any immediate action required for the safety of any included in this study, even if this action represents a deviation from the protocol. In such cases, Novartis should be notified of this action and the IRB/IEC at the study site should be informed according to local regulations.

[redacted]

15 References

[redacted]

Agarwal S and Cunningham-Rundles C (2007) Assessment and clinical interpretation of reduced IgG values. Annals of allergy, asthma & immunology. 99(3): 281-283.

Archelos JJ, Storch MK, and Hartung H-P (2000). The role of B cells and autoantibodies in multiple sclerosis. Ann Neurol 47: 694–706.

Bar-Or A, Fox E, Goodyear A, et al. (2020) Onset of B-cell Depletion with Subcutaneous Administration of Ofatumumab in Relapsing Multiple Sclerosis: Results from the APLIOS Bioequivalence Study. Presented at ACTRIMS; West Palm Beach, FL. P#LB300.

Bar-Or A, Grove RA, Austin DJ, et al (2018) Subcutaneous ofatumumab in patients with relapsing-remitting multiple sclerosis: The MIRROR study. Neurology p. e1805-e1814.

[redacted]

Bharmal M, Payne K, Atkinson MJ, et al. (2009) Validation of an abbreviated Treatment Satisfaction Questionnaire for Medication (TSQM-9) among patients on antihypertensive medications. Health Qual Life Outcomes; 7:36.

Bouaziz JD, Yanaba K, Venturi GM, et al. (2007) Therapeutic B cell depletion impairs adaptive and autoreactive CD4+ T cell activation in mice. PNAS 104 (52) 20878-20883

[redacted]

Frohman EM, Racke MK, Raine CS. (2006) Multiple Sclerosis - the Plaque and Its Pathogenesis N. Engl. J. Med., 354 pp. 942-955Genain CP, Hauser SL. (1996) Allergic Encephalomyelitis in Common Marmosets: Pathogenesis of a Multiple Sclerosis-like Lesion. Methods. 10(3):420-34.

Hauser SL, Bar-Or A, Cohen J et al. (2019) Efficacy and safety of ofatumumab versus teriflunomide in relapsing multiple sclerosis: results of the phase 3 ASCLEPIOS I and II trials.

[redacted]

Hauser SL, Bar-Or A, Comi G, et al (2017) Ocrelizumab versus Interferon Beta-1a in Relapsing Multiple Sclerosis. N. Engl. J. Med. p. 221-234.

Hauser SL, Waubant E, Arnold DL, et al (2008) B-cell depletion with rituximab in relapsing-remitting multiple sclerosis. N Engl J Med. 358(7):676-88.

Kappos L, Li D, Calabresi PA, et al (2011) Ocrelizumab in relapsing-remitting multiple sclerosis: a phase 2, randomised, placebo-controlled, multicentre trial. Lancet p. 1779-87.

[redacted]

Lehmann-Horn K, Kronsbein HC, and Weber MS (2013) Targeting B cells in the treatment of multiple sclerosis: recent advances and remaining challenges. Ther Adv Neurol Disord. 6(3): 161–173.

Lublin FD, Reingold SC, Cohen JA, et al (2014) Defining the clinical course of multiple sclerosis: the 2013 revisions. Neurology p. 278-86.

Lund, FE. Cytokine-producing B lymphocytes - key regulators of immunity. Curr. Opin. Immunol. 20: 332–338 (2008).

McFarland H. (2008) The B Cell - Old Player, New Position on the Team. N Engl J Med 2008; 358:664-665.

[redacted]

Pacheco-Fernandez T, et al. (2018) Anti-CD20 Antibodies Ofatumumab and Ocrelizumab Have Distinct Effects on Human B-cell Survival. AAN S52.003.

Pekny, M, Wilhelmsson, U. (2006). Intermediate filaments in astrocytes in health and disease. In Intermediate Filaments (ed. J. Paramio), pp. 10-26. Texas: Landes.

[redacted]

Polman CH, Reingold SC, Banwell B, et al (2011) Diagnostic criteria for multiple sclerosis: 2010 revisions to the McDonald criteria. Ann. Neurol. p. 292-302.

Salzer J, Svenningsson R, Alping P, Novakova L, et al (2016) Rituximab in Multiple Sclerosis: A Retrospective Observational Study on Safety and Efficacy. Neurology p. 2074-2081.

[redacted]

Sorensen PS, Lisby S, Grove R, et al (2014) Safety and efficacy of ofatumumab in relapsing-remitting multiple sclerosis: a phase 2 study. Neurology p. 573-81.

[redacted]

Teeling JL, Mackus WJ, Wiegman LJ, et al (2006) The biological activity of human CD20 monoclonal antibodies is linked to unique epitopes on CD20. J. Immunol. p. 362-71.

Theil D, Smith P, Huck C, et al. (2019) The Biological Activity of Human CD20 Monoclonal Antibodies Is Linked to Unique Epitopes on CD20. J Immunol. 177: (1) 362-37.

Thompson AJ, Banwell BL, Barkhof F, et al (2018) Diagnosis of multiple sclerosis: 2017 revisions of the McDonald criteria. Lancet Neurol p. 162-173.

Torres JB, Sealey M, Kneuer R, et al. (2019) Distribution and Efficacy of Ofatumumab and Ocrelizumab in Humanized-CD20 Mice following Subcutaneous or Intravenous Administration. Neurol. 92 (15 Supplement)

[redacted]

Wingerchuk DM, Banwell B, Bennett JL, et al. (2015) International consensus diagnostic criteria for neuromyelitis optica spectrum disorders. Neurol. 85:177-189.

[redacted]

16 Appendices

16.1 Appendix 1: Liver event and Laboratory trigger Definitions and Follow-up Requirements

Table 16-1 Liver event and laboratory trigger definitions

	Definition/ threshold
LIVER LABORATORY TRIGGERS	• 3 x ULN < ALT / AST ≤ 5 x ULN<br>• 1.5 x ULN < TBL ≤ 2 x ULN
LIVER EVENTS	• ALT or AST > 5 × ULN<br>• ALP > 2 × ULN (in the absence of known bone pathology)<br>• TBL > 2 × ULN (in the absence of known Gilbert syndrome)<br>• ALT or AST > 3 × ULN and INR > 1.5<br>• Potential Hy's Law cases (defined as ALT or AST > 3 × ULN and TBL > 2 × ULN [mainly conjugated fraction] without notable increase in ALP to > 2 × ULN)<br>• Any clinical event of jaundice (or equivalent term)<br>• ALT or AST > 3 × ULN accompanied by (general) malaise, fatigue, abdominal pain, nausea, or vomiting, or rash with eosinophilia<br>• Any adverse event potentially indicative of a liver toxicity*

*These events cover the following: Hepatic failure, fibrosis and cirrhosis, and other liver damage-related conditions; the non-infectious hepatitis; the benign, malignant and unspecified liver neoplasms TBL: total bilirubin; ULN: upper limit of normal.

Table 16-2 Follow up requirements for liver events and laboratory triggers

Criteria	Actions required	Follow-up monitoring
Potential Hy's Law case[a]	• Discontinue the study drug immediately<br>• Hospitalize, if clinically appropriate<br>• Establish causality<br>• Record the AE and contributing factors (e.g. conmeds, med hx, lab) in the appropriate CRF	ALT, AST, TBL, Alb, PT/INR, ALP and y-GT until resolution[c] (frequency at Investigator discretion)

[redacted]

Criteria	Actions required	Follow-up monitoring
**ALT or AST**		
> 8 × ULN	• Discontinue the study drug immediately<br>• Hospitalize if clinically appropriate<br>• Establish causality<br>• Record the AE and contributing factors (e.g. conmeds, med hx, lab) in the appropriate CRF	ALT, AST, TBL, Alb, PT/INR, ALP and y-GT until resolution[c] (frequency at Investigator discretion)
> 3 × ULN and INR > 1.5	• Discontinue the study drug immediately<br>• Hospitalize, if clinically appropriate<br>• Establish causality<br>• Record the AE and contributing factors (e.g. conmeds, med hx, lab) in the appropriate CRF	ALT, AST, TBL, Alb, PT/INR, ALP and y-GT until resolution[c] (frequency at Investigator discretion)
> 5 to ≤ 8 × ULN	• Repeat LFT within 48 hours<br>• If elevation persists, continue follow-up monitoring<br>• If elevation persists for more than 2 weeks, discontinue the study drug<br>• Establish causality<br>• Record the AE and contributing factors (e.g. conmeds, med hx, lab) in the appropriate CRF	ALT, AST, TBL, Alb, PT/INR, ALP and y-GT until resolution[c] (frequency at Investigator discretion)
> 3 × ULN accompanied by symptoms[b]	• Discontinue the study drug immediately<br>• Hospitalize if clinically appropriate<br>• Establish causality<br>• Record the AE and contributing factors (e.g. conmeds, med hx, lab) in the appropriate CRF	ALT, AST, TBL, Alb, PT/INR, ALP and y-GT until resolution[c] (frequency at Investigator discretion)
> 3 to ≤ 5 × ULN (participant is asymptomatic)	• Repeat LFT within the next week	Investigator discretion<br>Monitor LFT within 1 to 4 weeks

[redacted]

Criteria	Actions required	Follow-up monitoring
	• If elevation is confirmed, initiate close observation of the participant	
**ALP (isolated)**		
> 2 × ULN (in the absence of known bone pathology)	• Repeat LFT within 48 hours<br>• If elevation persists, establish causality<br>• Record the AE and contributing factors (e.g. conmeds, med hx, lab) in the appropriate CRF	Investigator discretion<br>Monitor LFT within 1 to 4 weeks or at next visit
**TBL (isolated)**		
> 2 × ULN (in the absence of known Gilbert syndrome)	• Repeat LFT within 48 hours<br>• If elevation persists, discontinue the study drug immediately<br>• Hospitalize if clinically appropriate<br>• Establish causality<br>• Record the AE and contributing factors (e.g. conmeds, med hx, lab) in the appropriate CRF	ALT, AST, TBL, Alb, PT/INR, ALP and y-GT until resolution[c] (frequency at Investigator discretion)<br>Test for hemolysis (e.g. reticulocytes, haptoglobin, unconjugated [indirect] bilirubin)
> 1.5 to ≤ 2 × ULN (participant is asymptomatic)	• Repeat LFT within the next week<br>• If elevation is confirmed, initiate close observation of the participant	Investigator discretion<br>Monitor LFT within 1 to 4 weeks or at next visit
Jaundice	• Discontinue the study drug immediately<br>• Hospitalize the participant<br>• Establish causality<br>• Record the AE and contributing factors (e.g. conmeds, med hx, lab) in the appropriate CRF	ALT, AST, TBL, Alb, PT/INR, ALP and y-GT until resolution[c] (frequency at Investigator discretion)
Any AE potentially indicative of a liver toxicity*	• Consider study drug interruption or discontinuation<br>• Hospitalization if clinically appropriate<br>• Establish causality	Investigator discretion

[redacted]

Criteria	Actions required	Follow-up monitoring
	• Record the AE and contributing factors (e.g. conmeds, med hx, lab) in the appropriate CRF	

[a]Elevated ALT/AST > 3 × ULN and TBL > 2 × ULN but without notable increase in ALP to > 2 × ULN [b](General) malaise, fatigue, abdominal pain, nausea, or vomiting, or rash with eosinophilia [c]Resolution is defined as an outcome of one of the following: (1) return to baseline values, (2) stable values at three subsequent monitoring visits at least 2 weeks apart, (3) remain at elevated level after a maximum of 6 months, (4) liver transplantation, and (5) death.

Based on Investigator's discretion investigation(s) for contributing factors for the liver event can include: Serology tests, imaging and pathology assessments, hepatologist's consultancy; obtaining more detailed history of symptoms and prior or concurrent diseases, history of concomitant drug use, exclusion of underlying liver disease.

[redacted]

16.2 Appendix 2: Specific Renal Alert Criteria and Actions and Event Follow-up

Table 16-3 Specific Renal Alert Criteria and Actions

**Serum Event**	**Actions**
Serum creatinine increase 25-49% compared to baseline	Confirm 25% increase after 24-48h<br>Follow up within 2-5 days
Acute Kidney Injury: Serum creatinine increase ≥ 50 % compared to baseline	Follow up within 24-48h if possible<br>Consider study treatment interruption<br>Consider participant hospitalization and specialized treatment
**Urine Event**	
New dipstick proteinuria ≥ 1+<br>Albumin- or Protein-creatinine ratio increase ≥ 2-fold<br>Albumin-creatinine ratio (ACR) ≥ 30 mg/g or ≥ 3 mg/mmol;<br>Protein-creatinine ratio (PCR) ≥ 150 mg/g or > 15 mg/mmol	Confirm value after 24-48h<br>Perform urine microscopy<br>Consider study treatment interruption/discontinuation
New dipstick glycosuria ≥ 1+ not due to diabetes	Blood glucose (fasting)<br>Perform serum creatinine, ACR
New dipstick hematuria ≥ 1+ not due to trauma or menstruation	Urine sediment microscopy<br>Perform serum creatinine, ACR

Additional specialized assessments are available to assess renal function or renal pathology. *(Note: In exceptional cases, when a nephrologist considers a renal biopsy, it is recommended to make slide specimen available for evaluation by the RSG to potentially identify project-wide patterns of nephrotoxicity.)*

Whenever a renal event is identified, a detailed participant history and examination are indicated to identify and potentially eliminate risk factors that may have initiated or contributed to the event:

- Blood pressure assessment (after 5-minute rest, with an appropriate cuff size)
- Signs and symptoms like fever, headache, shortness of breath, back or abdominal pain, dysuria or hematuria, dependent or periorbital edema
- Changes in blood pressure, body weight, fluid intake, voiding pattern or urine output
- Concomitant events or procedures such as trauma, surgical procedures, cardiac or hepatic failure, contrast media or other known nephroptoxin administration, or other diseases or causes, e.g. dehydration due to delirium, tumor lysis

[redacted]

Table 16-4 Renal Event Follow up

Follow-up of Renal Events
Assess, Document & Record in eCRF<br>• Urine dipstick and sediment microscopy evidence of DIN: crystals, red blood cells (dysmorphic/glomerular vs. non dysmorphic/non-glomerular), white blood cells, tubular epithelial cells<br>• Blood pressure and body weight<br>• Serum creatinine, BUN, electrolytes (sodium, potassium, phosphate, calcium), bicarbonate and uric acid<br>• Urine output<br>Review and record possible contributing factors to the renal event (co-medications, other co-morbid conditions) and additional diagnostic procedures (MRI etc.) in the eCRF<br>Monitor participant regularly (frequency at the Investigator's discretion) until:<br>• Event resolution: (sCR within 10% of baseline or PCT < 1 g/g Cr, or ACR <300mg/g Cr)<br>or<br>• Event stabilization: sCR within +/- 10% variability over last 6 months or PCR stabilization at a new level with +/- 50% variability over last 6 months<br>• Analysis of urine markers collected over the course of the DIN event

16.3 Appendix 3: Safety Monitoring Guidance

16.3.1 Guidance on monitoring of Participants with symptoms of neurological deterioration suggestive of PML

Should a participant develop any unexpected neurological or psychiatric symptom/signs in the opinion of Investigator (e.g. cognitive deficit, behavioral changes, cortical visual disturbances or any other neurological cortical symptoms/signs any symptom/sign suggestive of an increase of intracranial pressure) or accelerated neurological deterioration, the Investigator should schedule a complete physical and neurological examination and an MRI as soon as possible before beginning any steroid treatment. Conventional MRI as defined in the protocol as well as additional scanning such as FLAIR and Diffusion-weighted imaging (DWI) sequences are recommended to aid in differential diagnosis. The local neuro-radiologist must evaluate the MRI. The Investigator will contact the Medical Advisor at Novartis and a clinical trial team (CTT) member to discuss findings and diagnostic possibilities as soon as possible. A copy of the unscheduled MRI should be sent to the MRI Evaluation Center designated by Novartis as soon as possible. AE/SAEs need to be filed as appropriate.

If the MRI shows new MS lesions consistent with an MS relapse, assessment and treatment of the relapse will be performed as described in the protocol (Section 6.2.3 and Section 8.3.1). In case of new findings in the MRI images in comparison with the previous available MRI, which are not compatible with MS lesions, the study drug will be discontinued and other diagnostic evaluations need to be performed at the discretion of the Investigator. If new lesions are detected on the MRI, which may be infectious in origin, it is recommended to collect a cerebrospinal fluid sample if indicated. Analysis of the Cerebrospinal Fluid (CSF) sample including cellular, biochemical, Polymerase Chain Reaction, and microbiological analysis (e.g. herpes virus, JC virus) to confirm/exclude an infection should be performed. In the event of suspected CNS

[redacted]

infection (PML), a CSF aliquot should be sent to a central laboratory (designated by Novartis) for confirmatory testing.

Only after the evaluations have excluded diagnoses other than MS and after discussion with the Medical Advisor at Novartis and a CTT member, the study drug may be restarted.

16.3.2 Guidance on monitoring Participants with infections

All infections that develop during the study will be reported as AEs on the respective AE eCRF pages. Treatment and additional evaluations will be performed at discretion of the Investigator.

The Investigator should remind the participant of the risk of infections and instruct them to report any symptoms of infections promptly to the Investigator. The participants must also be reminded to always carry their participant Information Card (with site contact information and which identifies them as participants in a clinical study with investigational and control agents with potential immunosuppressive effects) and to show this to any local healthcare provider they may consult and ask that the Investigator be contacted.

In the case of suspected or confirmed serious (CTCAE, Grade 3-4) or atypical infection, study drug interruption should be considered. The Investigator should inform the Novartis Medical Advisor and/or a CTT member of any such cases.

When evaluating a participant with a suspected infection, the most sensitive tests available should be used (i.e. that directly detect the pathogen, as with polymerase chain reaction).

The Investigator should consider early treatment with specific antimicrobial therapy based on clinical diagnosis or suspicion thereof in consultation with infectious disease experts, as appropriate. The Investigator should inform the Novartis Medical Advisor and CTT of any such cases.

Investigators should consider the added immunosuppressive effects of corticosteroid therapy for treatment of MS attack/relapse and increase vigilance regarding infections during such therapy and in the weeks following administration.

In participants with known malignancies treated with ofatumumab, cases of fatal hepatitis B virus (HBV) reactivation and fatal infection due to hepatitis B in participants who have not been previously infected have occurred (refer to local Arzerra® prescribing information). The MS participants enrolled in the study who are at potential risk of HBV reactivation (e.g. Participants with evidence of prior HBV infection (antibody to hepatitis B surface antigen (anti-HBc) positive and Hepatitis B surface antigen (HBsAg) negative) and whose HBV deoxyribonucleic acid (DNA) test is negative at Screening, should be closely monitored for signs of active HBV infection or reactivation during the study. In participants with suspicion of HBV infection (active/reactivation), laboratory testing for HBV should be done. For participants who show evidence of hepatitis B infection (HBsAg positive [regardless of antibody status] or HBsAg negative but anti-HBc positive), the Investigator is advised to consult with physicians with expertise in managing hepatitis B regarding monitoring and consideration for HBV antiviral therapy.

In participants who develop reactivation of HBV while receiving study drug, immediately discontinue study drug and institute appropriate treatment and follow-up.

[redacted]

16.3.3 Guidance on immunization

The safety of and ability to generate a primary or anamnestic (recall) response to immunization with live, live-attenuated or inactivated vaccines during ofatumumab treatment has not been investigated. The response to vaccination could be impaired when B-cells are depleted.

It is recommended that the Investigator review the participant's immunization history as part of the initial Screening procedure for a participant being considered for treatment with ofatumumab.

Administration of live or live-attenuated vaccines must be avoided during and after treatment with ofatumumab and until B-cell counts are normalized.

There is presently no contraindication for the use of an inactivated, viral-vector-, or mRNA based Sars-CoV-2 vaccine in patients who are immunocompromised. However, different Sars-CoV-2 vaccines may have various mechanisms of action and different associated potential risks. Please review local prescribing information of any specific Sars-CoV-2 vaccine and comply with local prescribing information requirements for specific contraindications and special warnings and precautions for use. Vaccination against Sars-CoV-2 should be considered on a case-by-case basis at the discretion of the treating physician taking into account the individual benefit-risk assessment and local vaccination recommendations

16.3.4 Guidance on monitoring of Participants with low immunoglobulin levels

During the study, the immunoglobulin levels (IgG and IgM) will be measured according to the schedule in Table 8-1 and Table 8-2. If IgG levels were to drop below 300mg/dL (Agarwal and Cunningham-Rundles 2007), study drug treatment should be interrupted and the Investigator should evaluate participant for any potential infections and monitor on a regular basis. Immunoglobulin substitution therapy as per local medical practice is allowed. In case of treatment interruption, re-initiation of the study drug can only be considered once the immunoglobulin levels are back within normal limits.

[redacted]
